# Polyadenylation of insulin mRNA by Tent5a regulates pancreatic beta cells

**DOI:** 10.1038/s41467-026-72905-8

**Published:** 2026-05-20

**Authors:** Pamuditha N. Silva, Johanna E. Mayrhofer, Pavlo Potalitsyn, Geraldine O. Trüllinger, Svenja Godbersen, Nic Amstutz, Sebastian M. Siegner, Ben Kodiyan, Alexandra C. Title, Burcak Yesildag, Jacob E. Corn, Marc W. Schmid, Markus Stoffel

**Affiliations:** 1 Laboratory of Metabolic Diseases, Institute of Molecular Health Sciences, ETH Zürich, Zürich, Switzerland; 2 Laboratory of Genome Biology, Institute of Molecular Health Sciences, ETH Zürich, Zürich, Switzerland; 3 MWSchmid GmbH, Glarus, Switzerland; 4 Medical Faculty, University of Zürich, Zürich, Switzerland; 5 Present Address: InSphero AG, Schlieren, Switzerland

**Keywords:** Mechanisms of disease, Type 2 diabetes

## Abstract

Pancreatic beta cells can compensate for increased metabolic demand by increasing their function. While many studies have investigated mechanisms of beta cell decompensation during persistent metabolic stress, less is known how beta cells increase insulin synthesis and secretion. Here we identify the non-canonical cytoplasmic terminal nucleotidyltransferase Tent5a in a functional RNAi screen of RNA binding proteins as a positive regulator of insulin production. Tent5a expression positively correlates with obese and normoglycemic mouse models exhibiting compensated beta cell function, while its levels negatively correlate with beta cell failure. Increased Tent5a expression can be triggered by ribosome collisions and is repressed by ER stress and lipotoxic inflammation. Tent5a overexpression extended the poly(A) tails of insulin transcripts, enhanced mRNA stability and increased insulin content in INS-1E cells and human islets microtissues, while Tent5a knockout cells showed shortened Insulin mRNA half-life and reduced insulin content. Cytosolic polyadenylation activity is dependent on Tent5a tethering to the endoplasmic reticulum (ER) via Fndc3 proteins, leading to increased ER function. Our data identify a role of Tent5a in enhancing insulin production, which may be involved in boosting insulin synthesis and maintaining euglycemia in obesity.

## Abstract (web summary)

Here the authors show that Tent5a enhances insulin production in pancreatic beta cells by stabilizing insulin mRNA via cytoplasmic polyadenylation at the endoplasmic reticulum. Its expression rises with metabolic demand and declines in failure, suggesting a role in maintaining insulin output and glucose balance in obesity.

## Introduction

Functional plasticity is a key characteristic of pancreatic beta cells. In response to metabolic stress, these cells undergo dynamic adaptations to meet increased metabolic demands. Initially, beta cells compensate by enhancing insulin production and secretion, expanding beta cell mass, activating the unfolded protein response (UPR), and improving mitochondrial function. However, this compensatory phase is not sustainable over time. Prolonged oxidative and inflammatory stress leads to beta cell dysfunction, particularly affecting the endoplasmic reticulum and mitochondria^1,2^. As dysfunction progresses, beta cells may undergo phenotypic changes, including transdifferentiation and apoptosis^2^. While the molecular pathways driving beta cell failure are well characterized, the mechanisms that support beta cell compensation remain less thoroughly understood.

Rapid upregulation of insulin and proteins associated with hormone production and secretion define early beta cell compensation^3^. Post-transcriptional gene regulation (PTGR) is of paramount importance for the maturation, transport, stability, and translation of mRNAs^4^. RNA-binding proteins (RBPs) play a critical role in coordinating RNA processing and PTGR. Furthermore, RBPs are critical effectors of gene expression, and are therefore candidates regulating increased insulin expression and secretion during the compensatory phase of beta cells exposed to metabolic stress. So far, only few RBP have been linked to beta cell function, including RBFOX2, implicated in alternative splicing of genes required for insulin granule docking and exocytosis^5^, PTBP1 as a factor binding to the 5′ and 3′ end of insulin transcripts thereby regulating mRNA stability^3,6–8^, HUD/ELAVL4 as a negative regulator of insulin translation^9^, and TARDBP/TDP-43 influencing insulin secretion via Ca_V_1.2-mediated exocytosis^10^. Importantly, recent advances in the development of unbiased quantitative methods enabling the analysis of RNA protein interactions and in small molecule screens have revealed a “druggable” potential of RBPs as therapeutic targets^11^.

Nuclear mRNA polyadenylation is an essential PTGR governing the maturation of almost all eukaryotic mRNAs. Polyadenylation activity of the polyadenylation machinery is controlled by polyA binding proteins (PABPs) that associate with the nascent poly(A) tails and instruct termination of tail synthesis, leading to newly synthesized mRNAs of uniform in poly(A) length^12^. In addition to the nuclear polyadenylation pathway, cytoplasmic polyadenylation of mRNAs involves further elongation of the 3′-poly(A) tail after nuclear polyadenylation and export^13,14^. Increased poly(A) tail length correlates with augmented translational activity in embryonic tissue and enhanced mRNA stability, whereas poly(A) shortening sets off mRNA decay^15,16^. Canonical cytoplasmic polyadenylation is a well-characterized PTGR affecting some mRNA transcripts with a *cis-*acting cytoplasmic polyadenylation element (CPE) consensus sequence (5′-UUUUUAU-3′) in the 3′-UTR via the interaction of i. cleavage and polyadenylation specificity factor (CPSF) subunits, ii. CPE-binding protein (CPEB), and iii. the polyadenylation enzyme, GLD-2 or TENT2^16–21^. Within the broader superfamily of mRNA tailing terminal nucleotidyltransferases (TENTs), the TENT5 subfamily is composed of four highly-conserved, exclusively animal-expressed, paralogue enzymes (A to D) that have been implicated in CPE-independent, non-canonical cytoplasmic polyadenylation. While cytoplasmic polyadenylation activity has been reported in certain biological processes (e.g., oocyte maturation) and on a limited set of transcripts, their functions and relevance during conditions of metabolic stress remain unknown.

In this study, we conducted a functional siRNA screen targeting all RNA-binding proteins (RBPs) expressed in pancreatic beta cells, enabling the identification of both positive and negative regulators of insulin secretion. Among these, we identified Tent5a, a cytoplasmic nucleotidyltransferase that is markedly upregulated in compensated beta cells under genetic or metabolic stress. Tent5a promotes insulin production by extending the poly(A) tails of *Ins1* and *Ins2* transcripts, thereby enhancing insulin content and secretion. Loss of Tent5a function produces the opposite effect, leading to reduced insulin levels. Additionally, we demonstrate that Tent5a is anchored to the endoplasmic reticulum (ER) via interaction with Fndc3, a binding partner essential for the non-canonical polyadenylation of insulin mRNAs.

## Results

### RNAi screens reveal novel RBPs affecting beta cell function

**Fig. 1:**
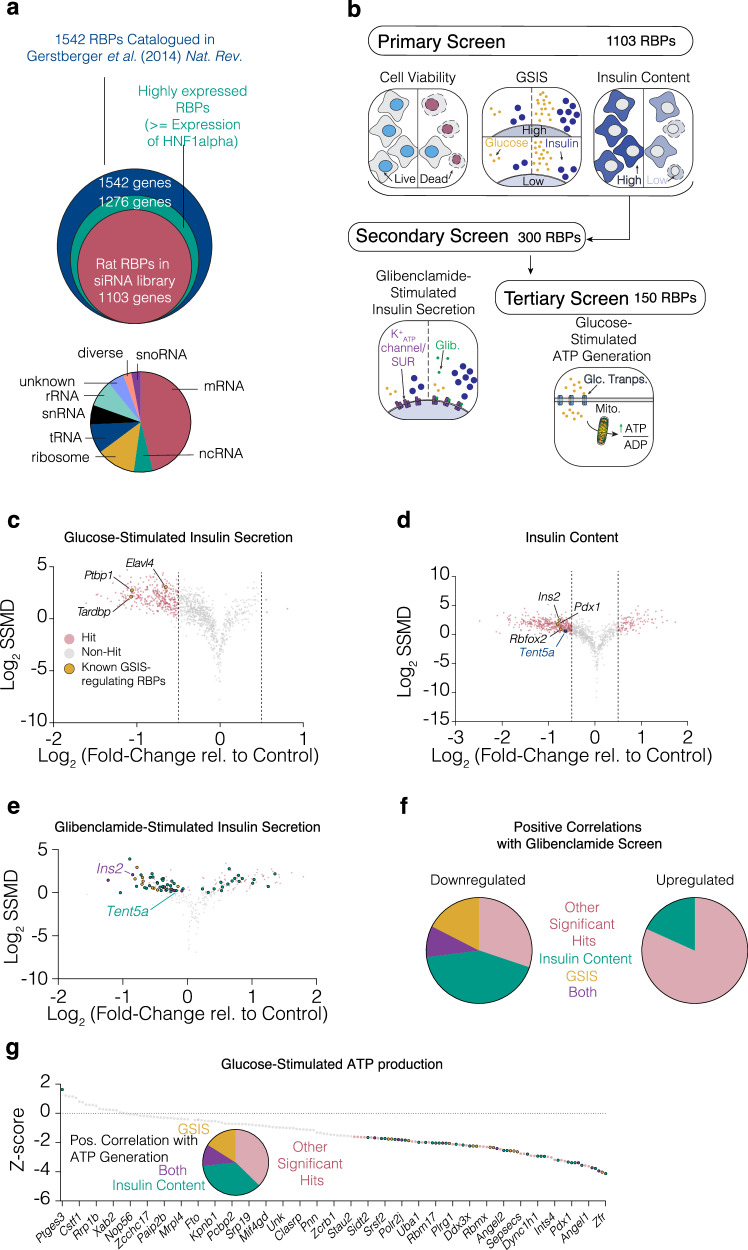
RNAi screen identifies novel RBPs regulating beta cell function. **a** Schematic of the number and composition of RBPs selected for the screen. Distribution of RBP classification provided in pie chart. **b** Schematic workflow of the multiparametric RNAi-based screen. Significant (*red*) and nonsignificant (*gray*) effect of RBP knockdown (KD) on glucose-stimulated insulin secretion (GSIS) (**c**), or cellular insulin content (**d**). Significant response identified as a fold-change of 1 and SSMD score of 1. Known RBPs and positive controls that affect beta cell function are highlighted in *yellow*. **e** Effect of RBP knockdown on glibenclamide-stimulated insulin secretion, color-coded to match positive correlation with GSIS (*yellow*), insulin content (*teal*), or both (*purple*). Significant hits identified as an SSMD of 1. **f** Proportion of RBPs exhibiting significant down- or upregulation in glibenclamide-stimulated insulin secretion sharing positive correlation with GSIS (*yellow*) or insulin content (*teal*) or both (*purple*). **g** Effect of RBP KD on glucose-stimulated ATP generation, color-coded to match positive correlation with GSIS (*yellow*), insulin content (*teal*), or both (*purple*). Inserted pie chart illustrates proportion of significantly regulated RBPs that share positive correlations to other readouts. |Z score| greater than 1.6 used to identify significant hits.

Although functionally diverse RNA-binding proteins (RBPs) are commonly involved in regulating both coding and non-coding RNAs across various molecular processes, their specific roles in pancreatic beta cell function remain largely unclear. To address this knowledge gap, we performed multi-parametric screens using RNAi to knockdown 1103 RBP transcripts from a consensus set of 1542 RBPs, based on meaningful expression levels in human pancreatic islets^4^. Genes selected included all RBP classes, with mRNA-binding proteins representing the majority (Fig. 1a and Supplementary Data 1). Cell viability, response to glucose-stimulated insulin secretion (GSIS), and insulin content were assessed in a primary screen, where selected transcripts with a standardized mean difference (SSMD) score above 1 in either GSIS or insulin content were targeted in secondary and tertiary screens to assess glucose-independent, glibenclamide-stimulated insulin secretion and ATP production (Fig. 1b)^22,23^. Interestingly, we found that silencing of 348 RBPs resulted in decreased GSIS (Log_2_ FC 0.5), while knockdown of only 3 transcripts resulted in increased insulin release (Fig. 1c and Supplementary Data 2). We also identified 415 RBPs that, upon silencing, reduced insulin content (Log_2_ FC 0.5) (Fig. 1d and Supplementary Data 3). Of all RBPs implicated in reduced GSIS or insulin content, 39% affected both outcomes. Importantly, silencing of *Tardbp*, *Ptbp1,* and *Elavl4*, all known to affect beta cell function, reduced insulin secretion, thereby validating our screen (Fig. 1c)^6,9,10^. Interestingly, we found reduced levels of total insulin and glibenclamide-stimulated insulin secretion upon silencing of terminal nucleotidyltransferase 5A (Tent5a), a cytoplasmic noncanonical poly(A) RNA polymerase that participates in the cytoplasmic polyadenylation (Fig. 1d, e)^21^. No enrichment was observed of any specific RBP class (i.e., mRNA, tRNA, rRNA, or ribosome-associated proteins) among those that influence beta cell function. Our data also showed that 22% of RBPs induced over 50% cell death upon knockdown, with some, such as Srsf10, reaching as high as 80% (Fig. S1a, b, and Supplementary Data 4). For glibenclamide-stimulated insulin secretion, a greater proportion of RBPs were associated with insulin content than with glucose-stimulated insulin secretion (19.3% vs. 5.3%, respectively) (Fig. 1e, f, and Supplementary Data 5). Lastly, when we assessed glucose-stimulated ATP generation in high (16.7 mM) glucose upon RBP silencing, 55.7% of tested RBPs exhibited a Z-score < −1.6 (Supplementary Data 6). Furthermore, 38.4% showed a positive correlation between GSIS and ATP production (Fig. 1g). Together, the data identified distinct and overlapping RBPs from diverse subfamilies as positive regulators of glucose- and sulphonylurea-stimulated insulin secretion, insulin content, and beta cell viability.

### Tent5a expression is induced with compensated beta cell stress

**Fig. 2:**
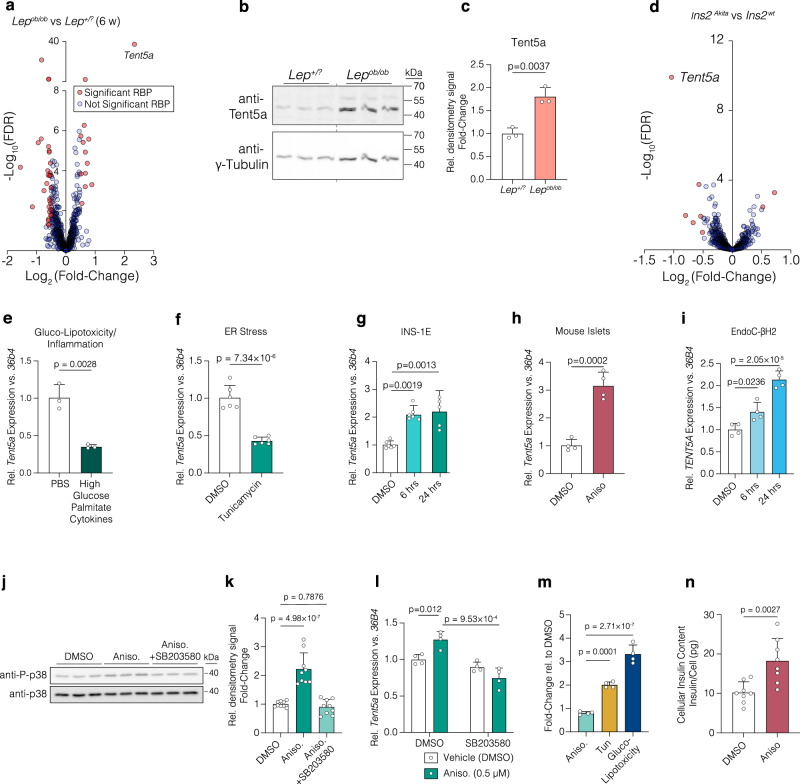
Tent5a is a highly regulated RBP gene induced during beta cell compensation and translational stress. **a** Differential expression analysis (DEA) of RBP transcripts of pancreatic islets from 6-week-old *Lep*^*ob/ob*^ compared to *Lep* ^*+/?*^ control mice. Significant (*red*) or nonsignificant (*blue*) transcripts shown based on a log_2_ (fold-change) cut-off of 0.5 and FDR value of 0.05. **b** Western blotting of Tent5a and γ-tubulin, and **c** densitometry from 7-week-old *Lep*^*ob/ob*^ islets and *Lep*^*+/?*^ controls (*n* = 3 for each group). **d** DEA of RBPs in 3-week-old *Ins2*^*Akita*^ mouse islets compared to WT controls. *Tent5a* expression following 24 h treatment with **e** a cocktail of 25 mM glucose, 0.4 mM palmitate, 100 ng/mL TNFɑ, 10 ng/mL IL1β, and 5 ng/mL IFNγ (glucolipotoxicity) compared to PBS (*n* = 3 each), or **f** tunicamycin (5 µg/mL) or DMSO (*n* = 6 each). *Tent5a* expression after anisomycin (0.5 µM) treatment with indicated concentrations and durations in **g**, INS-1E cells (*n* = 5 for 24 h, *n* = 6 for DMSO and 6 h), **h** mouse islets (*n* = 4 each) or **i** human EndoC-βH2 cells (*n* = 4 each). **j** Western blotting of phospho-p38 vs. total-p38 in INS-1E cells and **k** densitometry after a 15 min stimulus of 0.5 µM anisomycin or DMSO in the presence or absence of p38 inhibitor SB 203580 (40 µM). **l** *Tent5a* expression in INS-1E cells treated for 24 h with anisomyin (0.5 µM) or DMSO following a 1 h pre-incubation with DMSO (*left*) or of SB 203580 (40 µM). *n* = 4 for each condition. **m** Fold-change in proportion of apoptotic INS-1E cells after 24 h treatment with anisomycin, tunicamycin, or pro-inflammatory cytokine cocktail compared to DMSO controls. *n* = 4 for each condition. **n** Cellular insulin content normalized against islet cell numbers after 24 h treatment with either DMSO or anisomycin (0.5 µM). *n* = 8 sets of 20 size-matched islets for each condition. Data are presented as mean ± s.d. in (**c**, **e**–**i**, **k**–**n**). Two-sided unpaired t-test used to analyze data in (**c**, **e**, **f**, **h**, **n**). One-way ANOVA followed by Dunnett’s multiple comparison testing used to analyze data in (**g**, **k**–**m**). Two-way ANOVA with Šídák multiple comparison testing used to analyze data in (**l**).

The above RNAi screens identified diverse RBPs that are linked to beta cell function. In search of disease-relevant RBPs, we intersected RBPs that we identified in the screen as regulators of insulin secretion or production with RNA-seq expression data of pancreatic islets isolated from mice exposed to environmental or genetic stress. We first studied B6.Cg-Lep^*ob*^/J (*Lep*^*ob/ob*^) and high-fat, diet-induced obese mice (B6N-HFD) as preclinical models of beta cell compensation that, due to enhanced insulin secretion, maintain normoglycemia or mildly elevated glucose levels in ad libitum fed conditions, despite a progressive increase in obesity and insulin resistance (Fig. S2a, b)^24–26^. Analysis of islets derived from 6- to 12-week-old *Lep*^*ob/ob*^ mice revealed *Tent5a* to be the most up-regulated RBP transcript (Figs. 2a and S2c). Islets isolated from 28-week-old mice fed a high-fat diet (B6N, 45% fat, 17% sucrose) also exhibited enhanced *Tent5a* expression (Fig. S2d). Of note, *Tent5a* and *5c* were the most abundantly expressed Tent5a family members in pancreatic islets (Fig. S2e), and *Tent5a* transcript variant 2 (*TV2*) was the most regulated transcript (Fig. S2c, d). Tent5a upregulation was also confirmed in *Lep*^*ob/ob*^ mice at the protein level (Fig. 2b, c). In the C57BL/6-*Ins2*^*Akita*^/J (*Ins2*^*Akita*^) model, which harbors a point mutation in the *Ins2* gene causing beta cell decompensation and hyperglycemia due to ER-stress, Tent5a expression is reduced both at the RNA and protein levels^27–29^ (Figs. 2d and S2f, g). *Tent5a* is the only significantly regulated RBP transcript we identified with a direct correlation between compensating and decompensating islets (Fig. S2h). Importantly, we also found a correlation between the expression of INS and TENT5C, a close homolog of TENT5A, in pancreatic islets of human donors with type 2 diabetes (T2D) and impaired glucose tolerance, but not in nondiabetic individuals^30^ (Fig. S2i–k). Similarly, *Tent5c* and *Ins2* expression, as well as ER chaperone proteins, were also upregulated in *Lep*^*ob/ob*^ islets (Fig. S2l). These data indicate that in compensatory stress conditions, beta cells induce the expression of members of the TENT5 family across species.

We next investigated which pathophysiologically relevant stresses can influence the expression of *Tent5a* in beta cells. INS-1E cells treated for 24 h with a cocktail of high glucose, palmitate, and proinflammatory cytokines to mimic a glucolipotoxic environment, characteristic of later stages of beta cell failure, exhibited a > 50% reduction in *Tent5a* expression (Fig. 2e)^31^. Similarly, *Tent5a* expression was reduced when INS-1E cells were exposed to ER-stress via tunicamycin (Fig. 2f). Cells with dominant transcripts that are highly translated have been shown to be susceptible to ribosome stalling and collisions during translation^32^. To mimic these conditions, we treated INS-1E cells with anisomycin, a compound that occupies the peptidyl transfer center, retaining ribosomes to both nascent polypeptides and mRNAs to simulate ribosome collisions^33^. We observed strong induction of *Tent5a* expression in cells that were treated for 6 and 24 h with anisomycin (Fig. 2g). Anisomycin also activated *Tent5a* expression in primary mouse islets and human beta cells (Fig. 2h, i). Furthermore, anisomycin increased phosphorylation of p38 MAPK at Thr180/Tyr182, which is consistent with activation of p38 MAPK during ribosome collision (Fig. 2j, k)^33^, while inhibition of p38 prevented the anisomycin-induced upregulation of *Tent5a* (Fig. 2j–l). We next compared the impact of tunicamycin and anisomycin treatment on ER stress, cell survival, and function. While tunicamycin had an immediate effect on Eif2α phosphorylation and activation (P-EIF2α), anisomycin only induced P-Eif2α after prolonged treatment, consistent with the stronger activation of apoptosis by tunicamycin or glucolipotoxicity/inflammation compared to anisomycin (Figs. S2m and 2m). In line with anisomycin inducing increased *Tent5a* expression, we observed increased insulin content in islets treated with anisomycin (Fig. 2n). Together, these data reveal that Tent5a is induced in conditions of beta cell compensation linked to augmented insulin synthesis in a p38 MAPK-dependent manner, while its expression is reduced during severe ER stress and glucolipotoxicity, which are often associated with later stages of beta cell failure.

### Tent5a expression elongates mRNA poly(A) tails of insulin and increases mRNA stability

Intersection of the RNAi screen and transcriptomic analyses identified Tent5a as a potential positive regulator of beta cell function. To explore the role of increased Tent5a expression in pancreatic beta cells, we generated stable INS-1E cell lines expressing the tet repressor protein (TetR) with the mouse *Tent5a* transcript variant 2 isoform downstream of two *TetO* elements enabling doxycycline-inducible expression (Fig. S3a). Catalytic activity of Tent5a was predicted by sequence homology to be conferred by residues D125, D127, and E201 a finding supported by Alpha Fold 3 predictions situating the residues D125, D127 within the catalytic pocket close to the ligand ATP and the 3′ end of the *Ins2* poly(A) tail (Fig. S3b)^34^. To investigate the effect of Tent5a activity, we first compared expression of N-terminal HA-tagged wild-type Tent5a following doxycycline induction (referred to as “Tent5a WT”) against the catalytically inactive mutant bearing D125G and D127G substitutions (referred to as “Tent5a M1”) or uninduced control cells (referred to as “control”). Both lines increased *Tent5a* expression to approximately 5-fold after doxycycline treatment, with protein levels detectable as early as 6 h after doxycycline treatment and persisting as long as 48 h after induction (Fig. S3c, d). Subcellular localization of Tent5a WT and M1 by cellular fractionation and immunofluorescence microscopy revealed both forms to be predominantly cytosolic (Fig. S3e, f).

**Fig. 3:**
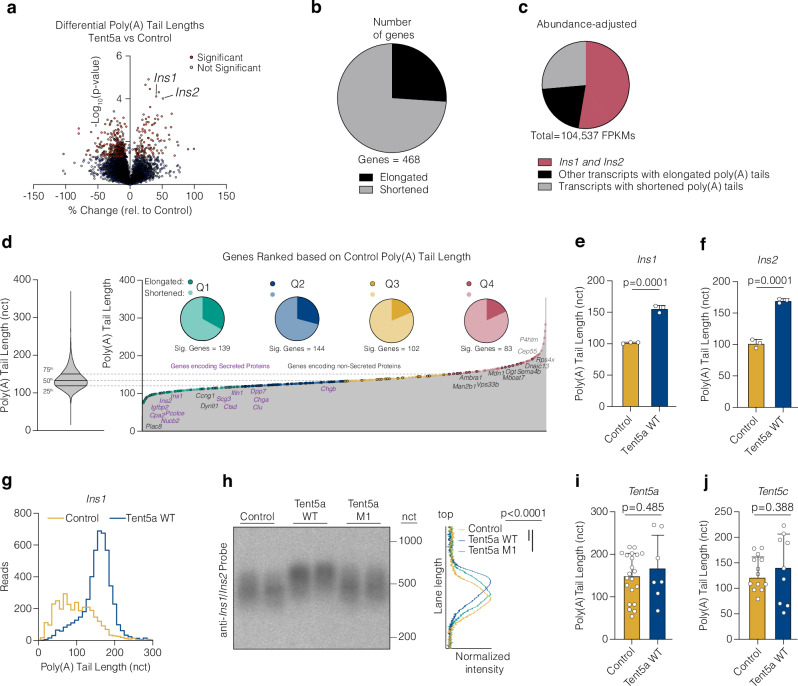
Tent5a activity elongates poly(A) tails of *Ins* mRNA. **a** Differential percentage change in poly(A) tail length between Tent5a WT overexpression versus controls. Significant (*red*) or nonsignificant (*blue*) transcripts were identified based on a percentage change cut-off of 5% and a -Log_10_(*p*-value of 0.05). **b** Fraction of genes exhibiting significantly elongated (*black*) and shortened (*gray*) poly(A) tail lengths. **c** Abundance-adjusted transcripts exhibiting significantly elongated (*black*), including *Ins1*, *Ins2* (*red*), and shortened (*gray*) poly(A) tail lengths. **d** Distribution of average poly(A) tail length per gene in uninduced control cells, indicating the boundaries between quartiles Q1–4 at the 25th, 50th, and 75th percentile. Genes positioned from shortest (*left*) to longest poly(A) tail length in uninduced controls, highlighting transcripts exhibiting significantly elongated (*dark, bound*) or shortened (*light*) poly(A) tail lengths upon Tent5a WT induction. Significantly regulated genes classified by basal poly(A) tail length from short (*teal* and *blue*) to long (*yellow* and *red*). Pie chart shading indicates the fraction of significantly elongated (*darker*) compared to shortened (*lighter*) transcripts. Average poly(A) tail length measured by FLAM-seq in uninduced control and Tent5a WT induced cells for **e** *Ins1* and **f** *Ins2*. *n* = 3 per condition for each gene. **g** Histogram of *Ins1* transcript poly(A) tail lengths measured in the control (*yellow*) and Tent5a WT (*blue*) induced cell conditions. **h** Northern blot autoradiograph generated by a ^32^P-labeled *Ins1*/*2* probe. Molecular weight marker represents nucleotide length (nct). *n* = 2 per condition. Lane profiles represent normalized signal intensity (x-axis) from top to bottom of uninduced control (*yellow*), Tent5a WT (*blue*), and Tent5a M1 (*teal*) lanes. *p*-values (based on F-test) derived from pair-wise comparison testing of means from approximated Gaussian distribution curves. Poly(A) tail lengths from FLAM-seq analysis of uninduced control (*yellow*) and Tent5a WT induced (*blue*) INS-1E cells of individual transcripts assigned to **i** *Tent5a* (*n* = 21 and 7 transcripts) and **j** *Tent5c* (*n* = 15 and 9 transcripts). Data are presented as mean ± s.d. in (**e**, **f**, **i**, **j**). FLAM-seq analysis with one-way ANOVA used in (**a**, **e**, **f**). Two-sided unpaired t-test used to analyze data in (**i**, **j**).

To explore the effect of *Tent5a* induction on poly(A) tail length control at the genome-wide level, we employed FLAM-Seq, which allows the determination of full-length sequences of endogenous mRNAs including their poly(A) tails^35^. We identified 468 transcripts, most notably *Ins1* and *Ins2*, that exhibited changes in poly(A) tail length when Tent5a WT overexpression was induced compared to controls (Fig. 3a). Elongated tails consisted exclusively of adenosine residues. Although only 26% of genes encode transcripts with elongated poly(A) tails, these transcripts make up 74% of the total abundance of transcripts that undergo changes in poly(A) tailing (Fig. 3b, c). Importantly, more than 50% of Tent5a-induced polyadenylated transcripts encoded for insulin. We also probed for preferences in Tent5a-induced poly(A) tail elongation. Genes were first ranked according to the average basal poly(A) tail length from shortest to longest and assigned to quartiles 1–4, respectively (Figs. 3d and S3f). Transcripts with shorter poly(A) tails were disproportionally more likely to be elongated with Tent5a expression compared to those already bearing longer poly(A) tails (Figs. 3d and S3g). Highly abundant transcripts and those encoding signal peptide-containing proteins, such as insulin, Nucb2, CgA, and CgB, were disproportionately overrepresented among Tent5a-induced elongated poly(A) tail transcripts (Fig. S3h, i). Notably, Tent5a expression increased the average poly(A) tail length of *Ins1* and *Ins2* from approximately 100 nt to 150 nt (Fig. 3e, f). Poly(A) tail elongation of *Ins1* transcripts resulted in a marked narrowing of the poly(A) length distribution (Fig. 3g). The average length increases of *Ins1* and *Ins2* were similar to other secreted proteins like *Pcolce*, *Cstd*, and *Igfbp2* (Fig. S3j–l). We also confirmed the Tent5a-dependent poly(A) tail elongation of *Ins* transcripts by Northern blotting where we noted increased *Ins1/2* transcript sizes in Tent5a WT-induced cells but not in catalytically inactive Tent5a M1 or uninduced control cells (Fig. 3h). Furthermore, we verified that overexpression of TENT5C, the isoform that correlated with insulin mRNA levels in human pre/diabetic islets (Fig. S2j, k), also leads to poly(A) elongation of insulin transcripts (Fig. S3m). Lastly, FLAM-Seq analysis showed that Tent5a overexpression does not change Tent5a and Tent5c poly(A) tail length, implying that Tent5a does not autoregulate itself (Fig. 3i, j). This finding is also consistent with the SignalP-noTM enrichment analysis showing that Tent5a preferentially target transcripts encoding secreted/ER-targeted proteins (Fig. S3i).

**Fig. 4:**
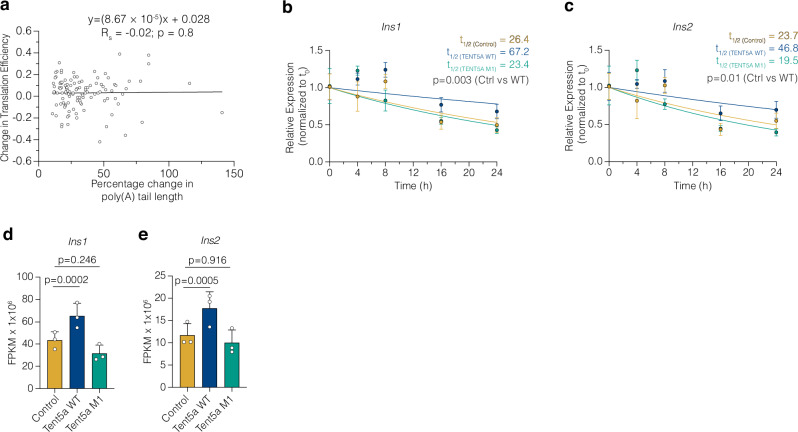
Tent5a activity extends the half-life of *Ins* mRNA. **a** Relationship between the percentage poly(A) tail length increase in significantly elongated genes due to Tent5a WT induction compared to uninduced control cells and accompanying fold-change in translation efficiency. Equation for the line of best fit, Spearman correlation coefficient (*R*_s_), and accompanied *p*-value provided. Relative expression of **b** *Ins1* and **c** *Ins2* over the course of treatment with actinomycin D (5 µg/mL) of uninduced control (*yellow*), Tent5a WT-(*blue*), and Tent5a M1-(*teal*) induced cells. *p*-values (based on F-test) indicate comparison of half-life (*t*_1/2_) between exponential decay curves generated for control and Tent5a WT data points. *n* = 6 replicates per time point per condition. Expression levels of **d** *Ins1* and **e** *Ins2* in uninduced control (*yellow*) cells compared to cells expressing Tent5a WT (*blue*) or Tent5a M1 (*teal*). *n* = 3 replicates per group per gene. Data are presented as mean ± s.d. in (**b**–**e**). DESeq2 analysis with Wald test and adjusted with Benjamini–Hochberg method used in (**d**, **e**).

We next investigated if poly(A) elongation influences translation by employing polysome profiling and sequencing, and mRNA stability assays by transcriptional inhibition using actinomycin D. We found that Tent5a-induced poly(A) elongation did not increase translation efficiency, as no overall increase in polysome fraction abundance of Tent5a target transcripts with elongated poly(A) tails were measured (Figs. 4a and S4a). However, we determined remarkably longer half-life (*t*_1/2_) of several mRNAs with increased poly(A) tail length, most notably, for *Ins1* and *Ins2* transcripts (Figs. 4b, c, and S4b, c). Half-life of *Ins1* and *Ins2* increased from 26.4 and 23.7 to 67.2 and 46.8 h, respectively, in Tent5a WT-induced vs uninduced cells, while no changes in *t*_1/2_ were measured in Tent5a M1 induced cells (Fig. 4b, c). Consistent with increased mRNA half-life, we observed increased *Ins1* and *Ins2* levels with Tent5a WT, but not M1 overexpression (Fig. 4d, e). In contrast, no changes in *t*_1/2_ were measured for transcripts with shorter poly(A) tails (Fig. S4d, e). Together, these data identify a subset of abundant transcripts, most notably mRNAs for insulin, with Tent5a-mediated elongated poly(A) tails, leading to profound increases mRNA stability and abundance.

### TENT5A expression regulates insulin production, even in decompensated human islets

**Fig. 5:**
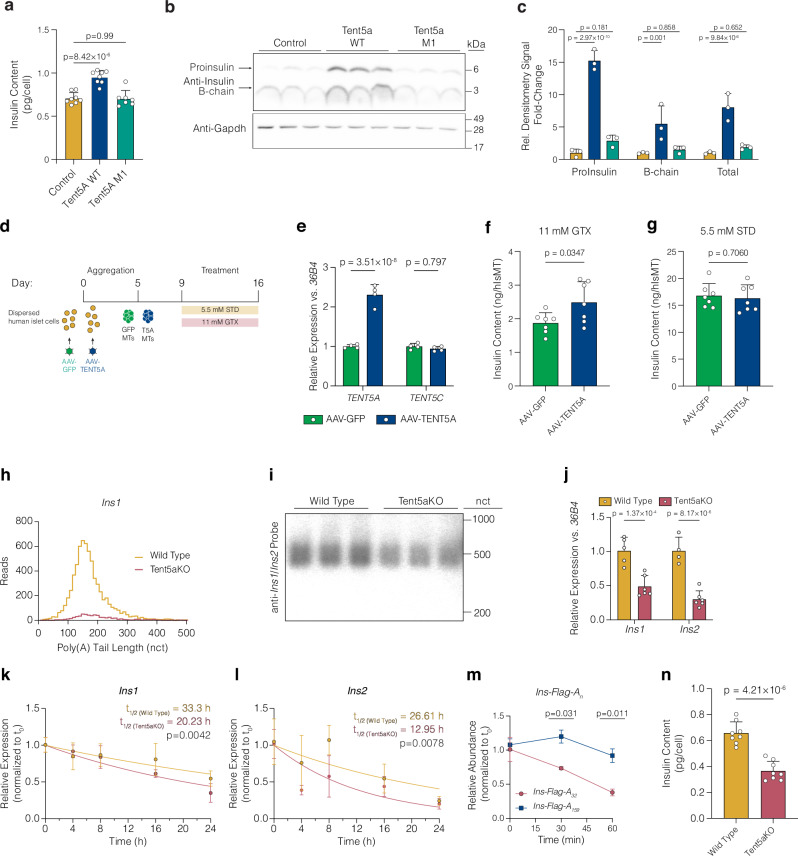
Tent5a expression regulates cellular insulin content. **a** Cellular insulin content of uninduced control (*n* = 8; *yellow*), Tent5a WT (*n* = 8, *blue*) and Tent5a M1 induced (*n* = 7, *green*) cells. **b** Western blot and **c** densitometry of tricine-based PAGE analysis of proinsulin and processed insulin in control, Tent5a WT, and Tent5a M1expressing cells. *n* = 3 for each group. Densitometry for each lane or total insulin signal normalized to GAPDH levels and reported as fold-changes compared to average control values. **d** Schematic of timeline for human primary islet microtissue (hIsMT) aggregation and infection with AAVs for GFP (green) or TENT5A (blue) overexpression, followed by treatment in standard (5.5 mM glucose, “STD”) or decompensation (11 mM glucose, “GTX”) conditions. **e** Relative mRNA expression of TENT5A or TENT5C in GFP- (green) or TENT5A-(blue) overexpression hIsMTs. *n* = 4 pools of 12 hIsMTs per replicate for each group. Cellular insulin content of GFP- (green) or TENT5A-(blue) overexpressing hIsMTs in **f** GTX or **g** STD conditions. =7 hIsMTs for each group. **h** Histogram of reads across distribution of *Ins1* transcript poly(A) tail lengths measured in wildtype (*yellow*) and Tent5a knockout (Tent5aKO, *red*) INS-1E cells. **i** Northern blot autoradiograph generated by a ^32^P-labeled *Ins1*/*2* probe of RNA samples from wild-type or Tent5aKO cells. Molecular weight marker represents nucleotide length (nct). *n* = 3 per condition. **j** Expression of *Ins1* and *Ins2* measured by qPCR in wildtype (*n* = 5) or Tent5aKO cells (*n* = 6). Relative expression of **k** *Ins1* and **l** *Ins2* over the course of treatment with actinomycin D (5 µg/mL) among wildtype (*yellow*) and Tent5aKO (*red*) cells. *p*-values (based on F-test) indicate comparison of half-life (*t*_1/2_) between exponential decay curves generated for wildtype and Tent5aKO data points. *n* = 6 per time point except *t*_0_ for wildtype (*n* = 5). **m** Relative abundance of in vitro*-*transcribed Ins-FLAG constructs with poly(A) tails contain 32 (*red*) or 159 (*blue*) adenosines, incubated for the indicated time points in cytosolic fractions extracted from INS-1E cells. **n** Cellular insulin content of wildtype (*yellow*) or Tent5aKO (*red*) cells. *n* = 8 for each group. Data as presented as mean ± s.d. in (**a**, **c**, **e**–**g**, **j**–**n**). One-way ANOVA followed by Dunnett’s multiple comparison testing used to analyze (**a**). Two-way ANOVA with Šídák multiple comparison testing used to analyze (**c**, **e**, **j**, **m**). Two-sided unpaired t-test used to analyze data in (**f**, **g**, **n**).

We next examined how increased mRNA stability and abundance influenced insulin production in beta cells, particularly within decompensating primary human islets. We measured an increase in cellular insulin content with a proportional increase in both pro- and mature insulin in INS-1E cells with Tent5a WT overexpression compared to both uninduced and Tent5a M1 overexpression (Fig. 5a–c). In addition, Tent5a WT induction also increased glucose-stimulated insulin secretion (Fig. S5a). Using a glucose-sensitive luciferase reporter driven by the human *INS* promoter, we measured no difference in luciferase activity between uninduced cells and those overexpressing wild-type Tent5a (Fig. S5b, c)^36^. Together, these data identify a direct link between Tent5a cytoplasmic polyadenylation activity and increased insulin production.

Impaired insulin production is a hallmark of beta cell exhaustion and decompensation, particularly in clinical conditions, such as type 2 diabetes. To assess whether TENT5A can improve primary human islet function, we generated human islet microtissues (hIsMTs) by reaggregating dissociated islet cells in the presence of adeno-associated virus (AAV) encoding either GFP or human TENT5A for 5 days (Fig. 5d). The resulting aggregates contained ~2000 cells, with ~50% expressing the beta cell marker NKX6.1 and transduction efficiencies exceeding 90% (Fig. S5d–g). Following reaggregation, hIsMTs were cultured for 7 days in either standard (STD; 5.5 mM glucose) or glucotoxic (GTX; 11 mM glucose) conditions. GTX treatment did not significantly affect endogenous *TENT5A* or *TENT5C* expression, while AAV-TENT5A led to a ~2-fold increase in *TENT5A* without altering *TENT5C* levels (Figs. S5h and 5e). TENT5A overexpression significantly increased insulin content in GTX-treated hIsMTs, but had no effect under STD conditions, where baseline insulin content levels were substantially higher (Fig. 5f, g). Similarly, glucose-stimulated insulin secretion was modestly enhanced in GTX-treated hIsMTs expressing TENT5A, with no change in response to low and high glucose stimulation under STD conditions (Fig. S5i, j). Collectively, these findings indicate that modest upregulation of TENT5A in primary human beta cells enhances insulin production under clinically relevant stress conditions.

Our findings so far have relied on overexpression models to uncover a novel role for cytoplasmic polyadenylation in insulin production. To investigate the effects of *Tent5a* loss-of-function, we generated a CRISPR-mediated Tent5a knockout INS-1E cell line (Tent5aKO). While no significant change in *Ins1* poly(A) tail length was detected, FLAM-seq revealed a marked reduction in *Ins1* transcript abundance, which was confirmed by Northern blot and qPCR analysis of *Ins1* and *Ins2* (Fig. 5h–j). Actinomycin D assays further showed a decreased mRNA half-life for both *Ins1* and *Ins2* in Tent5aKO cells compared to wild-type controls (Fig. 5k, l). We hypothesized that chronic loss of Tent5a results in transcripts with shorter poly(A) tails that are less stable at steady state, and therefore not detected in FLAM-seq. To test this, we generated in vitro transcribed *INS*-FLAG mRNAs containing either a short (~32 nt) or long (~159 nt) poly(A) tail (Fig. S5k). When spiked into diluted cytoplasmic extracts from INS-1E cells, the short-tailed *Ins*-FLAG-A_32_ transcript degraded more rapidly than the long-tailed *Ins*-FLAG-A_159_ (Fig. 5m), providing a mechanistic explanation for the unchanged poly(A) tail length distribution in Tent5aKO cells. Despite this, Tent5aKO cells displayed a significant reduction in both insulin content and glucose-stimulated insulin secretion, validating the phenotype originally identified in our RNAi screen (Figs. 5n and S5l). Together, these findings demonstrate that cytoplasmic polyadenylation contributes to the maintenance of basal insulin mRNA stability and expression, and that this process can be enhanced through increased Tent5a expression.

### Tent5a promotes the expression of ER genes, providing beta cells the means to facilitate insulin production

**Fig. 6:**
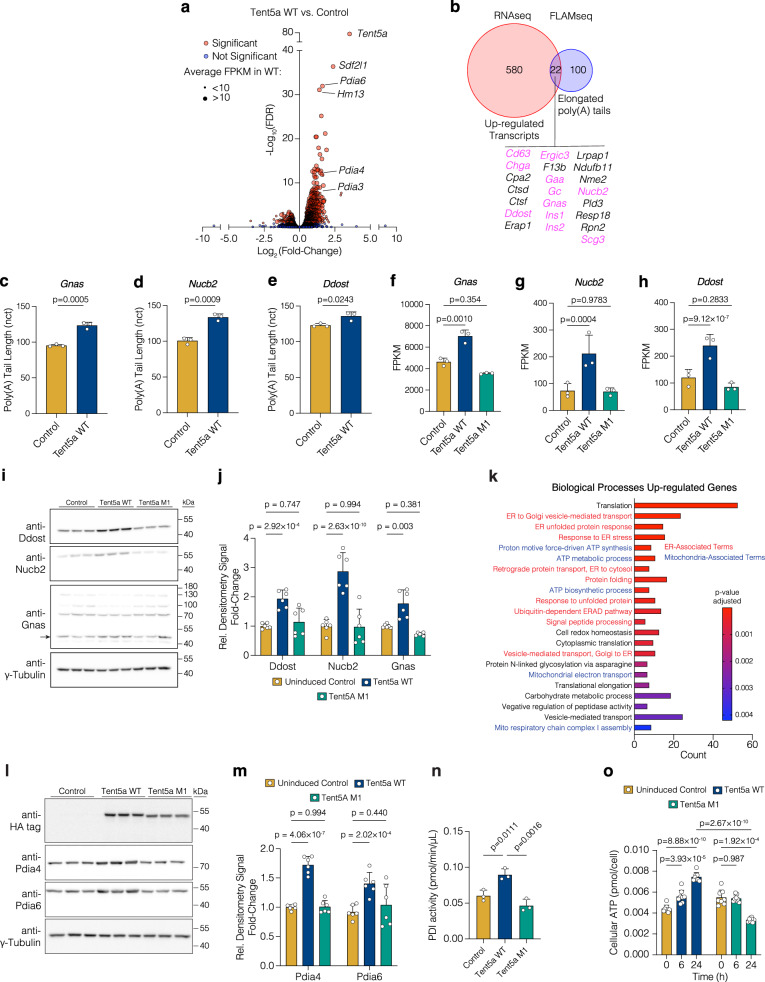
Tent5a activity promotes increased ER function. **a** Differential expression analysis (DEA) of INS-1E cells expressing Tent5a WT compared to uninduced controls. Significant (*red*) or nonsignificant (*blue*) transcripts were defined by an FDR cut-off of 0.01 and log_2_ (fold-change) of ± 0.5. Transcripts with average FPKM > 10 in control cells are represented as larger data points. **b** Overlap between elongated poly(A) tails (*blue*) and increased mRNA abundance (*red*) identified a gene subset with known beta cell functional significance (*pink*). Average FLAM-seq-measured poly(A) tail lengths in uninduced control and Tent5a WT expressing cells for **c** *Gnas*, **d** *Nucb2*, and **e** *Ddost*. *n* = 3. Expression levels of **f** *Gnas*, **g** *Nucb2*, and **h** *Ddost* in uninduced cells (*yellow*) compared to cells expressing Tent5a WT (*blue*) or Tent5a M1 (*teal*). *n* = 3 per group per gene. **i** Western blots (*n* = 3) and **j** Densitometry (*n* = 6) of proteins in uninduced compared Tent5a WT or Tent5a M1 overexpressing cells. **k** Top 22 biological process gene ontologies identified via over-representation analysis of up-regulated genes from Tent5a WT overexpression. Color-coded based on adjusted *p*-values, and text colored based on association with the ER (*red*) or mitochondria (*blue*). **l** Western blots (*n* = 3) and **m** densitometry (*n* = 6) of lysates of uninduced control cells compared to Tent5a WT or Tent5a M1 expressing cells. **n** Disulfide isomerase (PDI) activity from the lysate of either uninduced (*yellow*), Tent5a WT (*blue*), or Tent5a M1 (*teal*) overexpressing cells. *n* = 3. **o** Cellular ATP levels measured from uninduced control and Tent5a WT or Tent5a M1 overexpressing cells following doxycycline induction. *n* = 8 per condition per time point. Data are presented as mean ± s.d. in (**c**–**h**, **j**, **m**–**o**). DESeq2 analysis with Wald test and adjusted with Benjamini–Hochberg method used in (**a**, **f**–**h**). FLAM-seq analysis and one-way ANOVA used in (**c**–**e**). Overrepresentation analysis used with the hypergeometric test, and Benjamini–Hochberg method adjustment used in (**k**). Two-way ANOVA followed by Šídák multiple comparison testing used to analyze (**j**, **m**, **o**). One-way ANOVA followed by Dunnett’s multiple comparison testing used to analyze (**n**).

The increase in insulin abundance due to increased Tent5a expression confirmed the preliminary evidence from our RNAi screen and is functionally consistent with the transcriptional upregulation in obese mouse models of beta cell compensation^24,25^. We next investigated the extent to which induction of Tent5a WT or its inactive mutant M1 influences global beta cell expression by RNA sequencing. Differential expression analysis revealed several upregulated transcripts in response to Tent5a WT activation compared to uninduced cells, while Tent5a M1 induction yielded fewer abundant upregulated transcripts (Figs. 6a and S6a, b). Most Tent5a M1-induced mRNAs that were also regulated by Tent5a WT showed a negative correlation (Fig. S6b, c). These data suggest that the Tent5a WT-induced changes in gene expression are driven by Tent5a enzymatic activity rather than indirect effects of doxycycline treatment. The overlap between the larger set of up-regulated transcripts from RNA-seq and elongated poly(A) tails from FLAMseq identified only 22 common transcripts, suggesting that direct Tent5a-driven polyadenylation and increased mRNA half-life alone are not the only cause for the altered transcriptome when Tent5a is induced (Fig. 6b). However, among the 22 common transcripts to both datasets 11 genes have previously been reported to be positive regulators of insulin function (e.g., insulin secretion, content, granule biogenesis, or trafficking)^37–45^ (Fig. 6b). Similar to the effects observed with *Ins1* and *Ins2*, we examined representative genes, such as *Gnas*, *Nucb2,* and *Ddost*, with increased poly(A) tail length and mRNA abundance, and found that these changes were accompanied by elevated protein levels (Fig. 6c–j). A broader over-representation analysis revealed that ER- and mitochondria-associated gene ontology terms were prominent biological processes identified among upregulated genes (Fig. 6k). Indeed, Tent5a WT, but not Tent5a M1, increased the protein levels of ER chaperone proteins like Pdia4 and 6, despite exhibiting no change in poly(A) tail length (Fig. 6l, m, and S6d, e). *Gnas* encodes for the G-protein alpha subunit G_s_ɑ which is upstream of protein kinase A (PKA) signaling^45^. Interestingly, inhibition of protein kinase A (PKA) signaling using H-89 during Tent5a overexpression blunted the induction of the ER chaperone transcripts *Pdia4* and *Pdia6*, demonstrating that direct targets of Tent5a mediate further changes in gene expression independently of poly(A) tail elongation (Fig. S6f). The protein disulfide isomerases Pdia4 and 6 are also known as ER stress proteins that play a pivotal role in maintaining the redox homeostasis within the ER, which is critical for the proper folding of insulin^46–51^. Tent5a WT overexpression resulted in an increase in cellular protein disulfide isomerase (PDI) activity (Fig. 6n) and in ER volume, a correlation that has previously been reported (Fig. S6g, h)^52^. Of note, Tent5a WT expression, unlike Tent5a M1, induced *Ddit3* expression, a signal of ER stress; however, overexpression of neither WT nor M1 led to increased phosphorylation of Eif2ɑ (Fig. S6i–k). In addition, Tent5a WT induced an increase in cellular ATP levels, which is consistent with enhanced mitochondrial ATP production (Fig. 6o)^53–55^. Together, these results indicate that Tent5a activity directly and indirectly facilitates the orderly enhancement of beta cell function via increased insulin production through the augmentation of ER function.

### Fndc3a/b anchors Tent5a to the ER, serving as a hub for polyadenylation

**Fig. 7:**
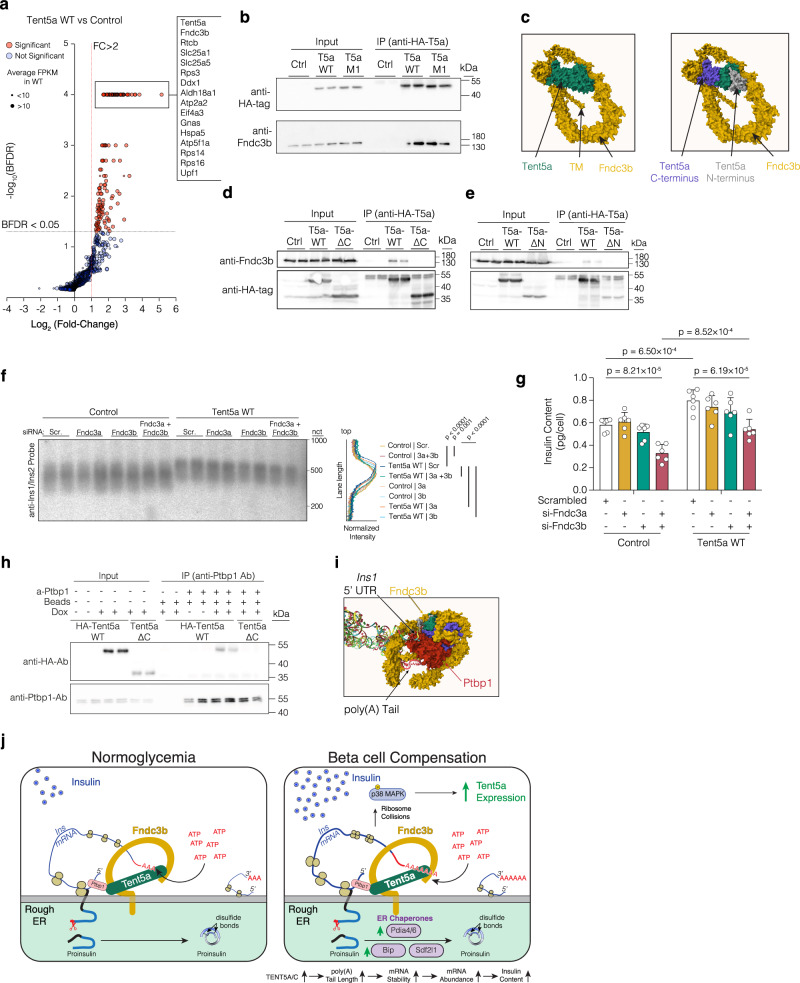
Fndc3a/b-Tent5a association at the ER promotes polyadenylation. **a** Identification of significant (*red*) proteins immunoprecipitated with Tent5a WT compared to lysates from uninduced control cells based on thresholds above a Bayesian FDR (BFDR) >0.05 and fold-change >2. **b** Western blots for HA-tag (of Tent5a) or Fndc3b following immunoprecipitation of HA-Tent5a WT and M1 with an anti-HA tag antibody (*right*) compared to the input (*left*). Uninduced cells were used as a negative control. **c** AlphaFold 3 molecular surface model of Fndc3b (*yellow*) in complex with Tent5a (*green*, *left*). The N- (*gray*) and C-terminus (*blue*) are color-coded in the model on the *right*. ipTM = 0.43 and pTM = 0.41. Western blots for the HA-tag (of Tent5a) or Fndc3b following immunoprecipitation of either HA-Tent5a WT, **d** Tent5a-∆C, or **e** Tent5a-∆N. **f** Northern blot autoradiograph generated by a ^32^P-labeled Ins1/2 probe from either uninduced or Tent5a WT-expressing cells exposed to siRNAs against *Fndc3a*, *3b*, both, or scrambled controls. Molecular weight marker represents nucleotide length (nct). *n* = 2 per condition. Lane profiles represent normalized signal intensity (x-axis) from top to bottom of the indicated conditions. *p*-values (F-test derived) from pair-wise comparison testing of means from approximated Gaussian distribution curves. **g** Cellular insulin content of Tent5a WT cell line in the absence (*left*) or presence (*right*) of doxycycline induction, exposed to siRNAs against Fndc3a (*yellow*) or 3b (*teal*), both (*red*) or scrambled control (*white*). *n* = 6. **h** Western blots for the HA-tag (of Tent5a) and Ptbp1 following immunoprecipitation of endogenous Ptbp1 in uninduced control cells or those induced with doxycycline to express Tent5a WT or Tent5a-∆C. **i** AlphaFold 3 model of Fndc3b (*yellow*) in complex with Tent5a (*green*, N- (*gray*) and C-terminus (*blue*)) and PTBP1 (*red* molecular surface). *Ins1* mRNA with a 50 bp poly(A) tail presented as a ribbon diagram, color-coded by nucleotide (cytosine–*blue*, adenosine –*red*, guanidine–*green*, uracil–*yellow*). ipTM = 0.31 and pTM = 0.32. **j** Graphical illustration of a model showing how TENT5A regulates mRNA polyadenylation, mRNA stability, and insulin production. Data are presented as mean ± s.d. in (**g**). Significance Analysis of INTeractome (SAINT) analysis with Bayesian FDR used in (**a**). Two-way ANOVA followed by Šídák multiple comparison testing used to analyze (**g**).

To gain a mechanistic understanding for the selective polyadenylation of secreted proteins, we performed immunoprecipitation and proteomic analysis of the HA-tagged Tent5a WT and Tent5a M1 constructs versus uninduced controls to identify interacting proteins (Fig. 7a). Notably, Tent5a WT and Tent5a M1 shared 60% of the same interactome, while 56 proteins were identified only in immunoprecipitations of Tent5a WT (Fig. S7a). While proteins with cytoplasmic localization were well represented within the interactome, ribosomal, mitochondrial, and ER-associated proteins were also identified (Fig. S7b). Despite the inclusion of proteins from these organelles in the interactome, we were unable to detect changes in the distance between the ER and mitochondria by FRET imaging as a result of Tent5a induction (Fig. S7c)^56^. However, we identified a highly significant interaction between Tent5a and ER-anchored Fndc3b that was confirmed biochemically by coimmunoprecipitations and agrees with a recent report identifying FNDC3 proteins as TENT5 family interaction partners (Fig. 7b)^57^. To address how Tent5a interacts with Fndc3b, we employed AlphaFold 3 modeling to predict the interaction domain(s) of the two proteins. AlphaFold predicted a 3-dimensional structure in which the N-terminal (1–77) as well as the C-terminal (294–430) end of Tent5a bind to Fndc3b (Fig. 7c). To test this experimentally, we generated HA- tagged C- and N-terminal deletion mutants of Tent5a, expressed them in INS-1E cells, followed by immunoprecipitations. While we were able to pull down Fndc3b with full-length Tent5a, both C- and N-terminal deletion mutants did not, thereby confirming the protein interaction (Fig. 7d, e). Furthermore, knockdown of both Fndc3a and 3b resulted in reduced localization between Tent5a and the ER by immunofluorescence and co-localization analysis (Fig. S7d–f). The shifts in co-localization were more pronounced when both Fndc3a and 3b were knocked down compared to the silencing of a single *Fndc3* family member. To test if poly(A) tail elongation of *Ins1*/*Ins2* is dependent on the interaction of Tent5a and Fndc3, we measured *Ins1/Ins2* transcript length after silencing Fndc3 by Northern blotting. Interestingly, we observed that the Tent5a WT-induced increase in *Ins1/Ins2* poly(A) length was progressively reduced with silencing of *Fndc3a* and *3b* alone or in combination (Fig. 7f). Concomitantly, disruption of this interaction reduced the Tent5a-induced increase in insulin content (Fig. 7g). Furthermore, silencing of *Fndc3a* and *3b* resulted in decreased expression of ER chaperone proteins Pdia4 and Bip despite Tent5a WT overexpression (Fig. S7g, h). At a functional level, deletion of Tent5a’s interaction domains abolished the upregulation of ER chaperone transcripts (Fig. S7i).

Previous reports have shown that the pyrimidine tract binding protein 1 (Ptbp1) binds to pyrimidine-rich segments of the untranslated regions of *Ins* transcripts^3,7^. Ptbp1 was highly enriched in our Tent5a interactome screen (Supplementary Data 7), and co-immunoprecipitation experiments confirmed this interaction (Fig. 7h). Furthermore, Ptbp1 co-immunoprecipitation with Tent5a persists in the absence of Fndc3a/b (Fig. S7j). Although, AlphaFold 3 predictions lag behind in terms of reliability for RNA structures, in particular, protein-RNA complexes, it predicted a close association of the pyrimidine-rich segment (CCC) of the *Ins1* 5′UTR with Ptbp1 (as indicated by a luciferase assay)^3^ and places the 3′ end of the poly(A) tail into the catalytic center of Tent5a in proximity to a free ATP substrate (Figs. 7i and S3b). Nevertheless, while FLAM-seq analysis showed that Ptbp1 silencing reduced *Ins1* transcript abundance, it did not affect the poly(A) tail length distribution during Tent5a WT overexpression—unlike Fndc3a/b silencing, which caused a leftward shift indicating shorter poly(A) tails (Fig. S7k). This indicates that PTBP1 is not required for Tent5a-mediated polyadenylation, and that its stabilizing effect is likely mediated via an alternative mechanism (e.g., 3′ UTR binding and protection from decay, which remains to be fully elucidated. Together, these findings suggest that the physical localization of Tent5a to the rough ER via Fndc3a, rather than its association with Ptbp1, is critical for the polyadenylation of target transcripts—most notably insulin—and for enhancing ER function.

## Discussion

RBPs are rational candidates to mediate processes leading to beta cell compensation during metabolic stress, as they are critical effectors of gene expression, recognize hundreds of transcripts, and form extensive regulatory networks that influence transcription, splicing, intracellular trafficking, protein translation, and decay^4^. Our systematic screens suggest that RBPs are mainly positive regulators of beta cell function and that they influence insulin secretion and cellular content. RBPs affecting these two readouts strongly overlapped, underlining consistent findings of reduced insulin content in islets with impaired insulin secretion^58^. Interestingly, single knockdown of relatively few RBPs resulted in severe cell death phenotypes, which likely is due to incomplete silencing by RNAi but also by genetic compensation through related RBPs. Furthermore, this resource database of RBPs implicated in beta cell function lays the foundation for mechanistic studies and for testing if RBP inhibitors are viable targets for novel antidiabetic therapies.

Intersection of functionally relevant RBPs expressed in beta cells with RNA seq data of hyperinsulinemic obese mice capable of maintaining close to normoglycemic glucose levels revealed a positive correlation between Tent5a expression and beta cell function. We showed that anisomycin stimulation, a model for ribosome collisions and stalling on abundantly expressed mRNAs, even when sustained for as long as 6 or 24 h, was sufficient to trigger a reversible upregulation in *Tent5a* expression. Anisomycin treatment for a short period (1 h) does not trigger phosphorylation of Eif2α compared to an identical treatment period with tunicamycin. Longer exposure (24 h) of beta cells to anisomycin did induce ribosomal stress but resulted in less apoptosis than cells exposed to tunicamycin or glucolipotoxic conditions for the same period. Interestingly, we did not observe increased ER stress with acute overexpression of Tent5a. The discrepancy between anisomycin treatment and selective Tent5a overexpression may be explained by anisomycin causing nonselective ribosome collisions on all translated transcripts, whereas Tent5a activity leads to increased abundance of transcripts encoding secreted proteins at the ER. Furthermore, insulin transcripts, although abundant, are short and therefore less prone to ribosome collisions and stalling, making it less likely to activate ribosomal stress responses. Interestingly, anisomycin treatment of islets and INS-1E cell resulted in increased insulin content and insulin secretion, a finding consistent with the notion that p38-activated ribosomal stress is an early and reversible stress that correlates with increased insulin production. The conserved anisomycin-induced upregulation of Tent5a in mouse, rat and human pancreatic beta cells is in contrast to the downregulation of Tent5a observed upon stresses associated with later-stage beta cell failure, including pharmacologically induced ER stress (tunicamycin) and proinflammatory cytokines, and in the Akita model that expresses a mutant insulin allele in which the C96Y residue can no longer engage in a crucial disulfide bond, leading to the formation of irreversible misfolded high-molecular-weight protein complexes, ER stress and beta cell apoptosis^29,59,60^.

Among all cytosolic polyadenylation-modifying enzymes, Tent5 proteins have been the least studied. Genetic inactivation of Tent5a in mice has revealed that it polyadenylates mRNA encoding extracellular matrix constituents crucial for bone mineralization and during osteoblast mineralization, a finding that is consistent with a report showing that Tent5-mediated polyadenylation of mRNAs is enriched for secreted proteins during gametogenesis in mice^61^. Prior work on cytoplasmic polyadenylation has focused on developmental or embryogenic contexts with the argument that in the absence of more sophisticated or developed transcriptional regulation, polyadenylation serves as a rapid and readily available mechanism to exert translational control^14,15^. Advances in poly(A) tail sequencing however, point to a greater diversity in tail composition and roles for tail length changes in stress conditions^62^. Our data support a specific role for Tent5a in extending the poly(A) tails of insulin transcripts, as the selective overexpression of this terminal adenosine transferase elongated poly(A) tails. Unlike in embryogenesis, we did not observe a strong positive correlation between increased poly(A) tail length and translation efficiency, but rather a more profound impact on mRNA stability, which ultimately exhibited in greater protein, namely insulin, abundance. Our findings that CRISPR-mediated knockout of Tent5a strongly reduces insulin transcript abundance demonstrates that it fulfills a fundamental role in insulin production, not only in stress but also homeostatic conditions. We were unable to implicate the canonical poly(A) pathway in this process, since neither Cpeb, Gld-2, nor most subunits of Cpsf were identified in the RNAi screen or upregulated in genetic or diet-induced obese models with compensated islet function. Furthermore, insulin transcripts do not contain the canonical CPE motif that is typically found in transcripts with canonical Gld-2 polymerase-induced elongated poly(A) tails. Here, we identify noncanonical polyadenylation via Tent5 proteins in the regulation of hormone production in response to increased demand. Future work may reveal that this is a generalizable mechanism by which hormone-producing cells increase their output in pathophysiological conditions.

Beta cells normally respond to alterations in metabolic demands with a proportional synthesis and secretion of insulin. However, increased insulin demands, i.e., during insulin resistance or the loss of β-cell mass, creates an increased synthetic demand on residual β cells, that can trigger ER stress. Surprisingly, the induction of Tent5a and subsequent increase in insulin production did not upregulate Eif2ɑ phosphorylation, a hallmark of the ER stress response. Our data shows that Tent5a expression correlated with increased chaperones (Pdia3,4/6) expression and PDI activity, however, the expression of proinsulin cleavage enzymes was not affected. Increased expression of the former is dependent on Tent5a activity (WT triggers upregulation, M1 mutant does not) despite not being a direct target of Tent5a (no elongated poly(A) tail). These observations indicate that Tent5a activates the expression and activity of proteins that occur co-translationally or shortly after the proinsulin is translocated into the ER^63^, whereas later processing reactions that occur in the Golgi apparatus are not affected^63,64^. Also, PDI can retain proteins within the ER, thereby further accommodating for the increased insulin translation^65^, consistent our data showing no increase in ER stress in response to Tent5a overexpression.

We found that the expression of Fndc3a/b, a family of integral ER-membrane proteins that bind Tent5 proteins^57^ is essential for catalytic Tent5a activity on insulin transcript poly(A) elongation and the secondary effects of increasing ER chaperone proteins. Using colocalization analysis, we demonstrate that Fndc3a/b recruits Tent5a, thereby bringing its terminal nucleotidyltransferase activity in close proximity to the ER, where insulin transcripts are translated. The enhanced ER function resulting from increased Tent5a activity is likely due to both its localization to the organelle, which facilitates polyadenylation of rough-ER targeted transcripts, and the promotion of mRNA stability. A previous study has shown that Ptbp1 expression influences the half-life of insulin mRNA, however the mechanism remained elusive^3^. Here, we identified Ptbp1 as an interaction partner of Tent5a in both an unbiased proteomic pulldown and co-immunoprecipitation experiments. We attempted to use AlphaFold 3 to predict the interaction between Tent5a with both Fndc3b and Ptbp1. We found that Ptbp1 does not mediate poly(A) elongation, suggesting that its effects on insulin transcript stability is independent of Tent5a. Instead, our findings identify Tent5a as a novel mediator to target insulin transcripts to the rough ER, where not only is the hormone translated, but dynamics of mRNA stability via noncanonical cytoplasmic polyadenylation are regulated (Fig. 7l).

We also tested the effect of TENT5A overexpression in human islet micro-tissues (hIsMTs) to explore if enhanced TENT5A activity could translate to improved function of a stress-induced human T2D model^66,67^. With only a modest 2-fold upregulation of TENT5A, we measured a ~30% increase in insulin content, specifically in hIsMTs mimicking a state of decompensation, suggesting that TENT5A may be an interesting therapeutic target. This improvement warrants further studies to probe whether a further increase in TENT5A activity could ameliorate beta cell function. It would also be interesting to explore if the enhancement of the TENT5A–FNDC3 interaction through small molecules could further increase insulin production. Alternatively, silencing of TENT5A or FNDC3 in pancreatic beta cells could be a therapeutic means of reducing insulin production in insulinomas and endogenous hyperinsulinism caused by functional β-cell disorders, such as nesidioblastosis^68^.

In summary, our study demonstrates that induction of Tent5a-mediated poly(A) tail elongation of insulin transcripts is a key determinant of pancreatic beta cell to respond to increased metabolic demand by targeting Tent5a activity to the ER, prolonging insulin transcript stability, enhancing insulin processing in the ER, thereby increasing insulin synthesis and secretion. Pharmacological interventions activating TENT5A activity may result in delaying beta cell decompensation and the development of T2D.

## Methods

### Mouse models

All animal experiments were in accordance with institutional guidelines and approved by the Kantonale Veterinäramt Zürich. C57Bl/6 and *Lep*^*ob/ob*^ (B6.Cg-*Lep*^*ob*^/J) mice were purchased from Charles River. All mice were housed in a pathogen-free animal facility at the Institute of Molecular Health Sciences at ETH Zurich. Mice were maintained on a 12 h light/dark cycle (lights on from 6:00 to 18:00). Mice were fed a standard laboratory chow diet.

### Islet isolation and processing

Mouse pancreatic islet isolation was performed as previously described^69^. After the ampulla of Vater was clamped, 2 mL of Liberase (5 mg/mL; Sigma 05401127001) diluted in RPMI buffer (Thermo Scientific) was injected through the common bile duct. The pancreas was excised and digested for 18 min at 37 °C prior to neutralization with RPMI media containing 10% FBS and mechanical shearing. The digested pancreas was pelleted by centrifugation and resuspended in Histopaque^TM^-1077 (Sigma) and overlayed with an equal volume of FBS-free RPMI media to separate islets from exocrine cells via centrifugation (900 × *g*, 20 min with the lowest acceleration/deceleration speeds). Islets were further purified through a 70 µm cell strainer. Islets were cultured in RPMI 1640 media supplemented with FBS (10%), penicillin/ streptomycin (100 U/mL), HEPES (10 mM; pH 7.4), GlutaMAX (1 mM), and sodium pyruvate (1 mM; Thermo Scientific) for at least 3 h before being treated or processed for RNA or protein harvesting. Islets from three 7-week-old B6-*ob/ob* mice were equally distributed for protein and RNA harvesting, and islets from six 7-week-old control animals were harvested for three RNA and protein samples each. For RNA or protein harvesting, islets were first picked into 1 mL 1× PBS and centrifuged at 129 × *g* for 1 min. The 1× PBS was aspirated and replaced with 1 mL TRIzol for RNA or 50 µL of RIPA buffer for protein extraction.

### INS-1E culture

INS-1E cells used in this study originate from a stock at passage 93 that was expanded from a single clone that exhibited a two-fold increase in GSIS from 3.3 to 16.7 mM glucose. INS-1E cells were maintained on 10-cm culture plates with RPMI 1640 (Thermo Scientific) supplemented with FBS (10%), penicillin/streptomycin (100 U/mL), HEPES (10 mM; pH 7.4), GlutaMAX (1 mM), sodium pyruvate (1 mM; Thermo Scientific), and β-mercaptoethanol (71.5 µM; Sigma) as previously described^69^.

### EndoC-βH2 culture

EndoC-βH2 cells (Human Cell Design) were seeded onto culture plates coated for at least 1 h at 37 °C and 5% CO_2_ with DMEM media (4.5 g/L glucose; Thermo Scientific) containing Matrigel (1%; Corning) and fibronectin (1 µg/mL; Corning) and penicillin/streptomycin (100 U/mL; Thermo Scientific). They were cultured in Optiβ1 media (human cell design) as per the manufacturer’s procedure.

### HEK293T cell culture

HEK293T cells were maintained on 15 cm culture plates with DMEM (4.5 g/L glucose; Thermo Scientific) supplemented with FBS (10%), penicillin/streptomycin (100 U/mL), HEPES (10 mM; pH 7.4), GlutaMAX (1 mM), and sodium pyruvate (1 mM; Thermo Scientific).

### Plasmid generation

The mouse *Tent5a* transcript variant 2 isoform was PCR amplified from islet cDNA of 6-week-old C57Bl/6 mice using primers PS-Tent5a-C-F2 and PS-Tent5a-C-R (sequences provided in Supplementary Data 8). The band was gel extracted and cloned into the pcDNA3.1 vector (Thermo Scientific) via the *EcoRV* and *BstBI* sites to generate the intermediate vector pcDNA3.1-mTent5a WT. This plasmid was used to re-amplify Tent5a, this time with an N-terminal HA-tag sequence and TAA stop codon after the coding sequence in two sequential rounds of PCR, first with the primer PS-C-N-HA-T5A-F1 and then the primer PS-C-N-HA-T5A-F2, both with the primer PS-Tent5a-GA-R4. This PCR fragment was cloned into the pLenti-puro vector (Addgene #39481) between the restriction sites *XhoI* and *BamHI* to generate the vector pLenti-puro-mTent5a WT.

Tent5a M1 mutant was generated by site-directed mutagenesis via PCR amplification using primers PS-mT5Am1-F and PS-mT5Am1-R and Phusion polymerase (New England Biolabs) with the vector pcDNA3.1-mTent5a WT as the template. This PCR reaction was subjected to a combined ligation/digestion treatment with T4 polynucleotide kinase, T4 ligase, and DpnI enzyme (New England Biolabs). The resultant vector was used as a template for subcloning again into the pLenti-puro vector in the method described above to generate the vector pLenti-puro-mTent5a M1.

The truncation mutants of Tent5a were cloned in a similar manner as pLenti-puro-mTent5a WT. The N-terminus removed construct (Tent5a-∆N) was first PCR amplified with primers PS-C-N-HA-T5A-F1 with PS-Ntase-R before being re-amplified with PS-C-N-HA-T5A-F2 and PS-Ntase-R. The C-terminus removed construct (Tent5a-∆C) was PCR amplified once with primers PS-Ntase-F and PS-Tent5a-GA-R4.

Ins1-Flag-A_n_ plasmids were generated by first performing reverse transcription of RNA harvested from INS-1E cells using a self-complementary reverse primer (PSPP-Ad3-BsmI-A14), which would add a *BsmI* restriction site and universal anchor immediately 3′ of the poly(A) tail of mRNAs. *Ins1* cDNAs with variable poly(A) tail lengths were amplified via PCR with an *Ins1*-specific forward primer (PS-rIns1-5-F) and universal anchor-targeting reverse primer (PS-BsmI-Rev), introduced into the pcDNA3.1 vector via TA cloning, and transformed into *E.coli Top10* (Thermo Scientific). Plasmids harboring *Ins1* cDNAs with unique poly(A) tail lengths were isolated from individual bacterial clones and sizes estimated by Sanger sequencing (Microsynth). Two plasmids bearing poly(A) tails estimated at 32- and 159-nt long were selected for site-directed mutagenesis as described above using the primers FLAG-rIns1A-S and -AS to introduce a Flag tag sequence in frame with the C-terminus of the insulin coding domain.

The eroGFP construct was amplified from the parentERroGFP_iE_pCDNA3 vector (Addgene #47954) using primers “RoGFP-F” and “RoGFP-R” and cloned via Gibson assembly into the pLenti-CMV-GFP Hygro (Addgene #17446) plasmid by removing the GFP cassette through restriction digest with *SalI* and *BamHI*. The resultant vector pLenti-ERroGFP was used for lentivirus production.

### Lentivirus production

The vectors pLenti-puro-Tent5a WT, -Tent5a M1,-Tent5a-∆C, Tent5a-∆N GW1-PercevalHR (Addgene #49082), pLenti-ERroGFP, and pLKO-TetR-neo (Addgene #13425) were packaged as lentiviruses via transfection into HEK293T cells as previously described^70^. Briefly, 10 µg of these plasmids were co-transfected with the packaging vector pCMV-dR8.2 dvpr (Addgene #8455) and pCMV-VSV-G (Addgene #8454) in a ratio of 2:2:1 into HEK293T cells with 1:1 polyethylenimine linear MW 25,000 transfection reagent (PolySciences) in OptiMEM media (Thermo Scientific) and incubated for 6 h at 37 °C and 5% CO_2_. Media was replaced with HEK293T cell media and cells were incubated for 48 h additionally before supernatants were clarified through a 45 µm syringe filter and ultracentrifuged at 84,800 × *g* for 6 h, at 4 °C. Viral pellets were resuspended in 1× PBS and stored at −80 °C before being used to infect INS-1E cells.

### Stable cell line generation

INS-1E cells at passage 20 were infected with PercevalHR lentivirus at an MOI of 4 before being sorted for positive fluorescent protein signal by FACS using excitation parameters previously described^23^. Single clones were selected and assessed for a robust glucose-stimulated ATP-generation response.

INS-1E cells at passage 22 were infected with TetR lentivirus at an MOI of 4 before being subjected to G418 selection (Thermo Scientific; 800 µg/mL) 6 days post transduction. Cells were expanded for an additional 4 passages prior to re-infection with the lentiviruses encoding the Tent5a WT, Tent5a M1, Tent5a-∆N, or Tent5a-∆C constructs and subjected to puromycin selection (Thermo Scientific; 1 µg/mL) 6 days post transduction. Cells were expanded and continuously selected with puromycin and G418 during routine maintenance, but removed during experimentation. All experiments were performed between passages 33 and 50.

Stable cells containing both the TetR and HA-Tent5a WT constructs were again infected with lentivirus encoding ERroGFP and used within passages 40 and 50.

### Human islet microtissue culture conditions and transduction

Human islet microtissues (hIsMTs) were generated from ~2000 dispersed and re-aggregated primary human islet cells from a single donor (UNOS ID# AMAX293, 52 y.o., female, white, BMI 28.43, positive serologies: CMV IgG, EBV IgG, HbA1c: 5.2%, cause of death: anoxia, mechanism of death: drug intoxication, normal islet classification; InSphero AG) as previously described^66,67^. hIsMTs were transduced during the first 5 days of reaggregation in Akura hanging drop plates (InSphero) at an MOI of 3 × 10^5^ viral genomes per cell with KP1 serotype AAVs encoding for GFP or TENT5A (RefSeq: BC007351; Vector Biolabs). hIsMTs were then released into Akura 96-well plates before they were cultured in standard human islet maintenance medium (InSphero). Nine days after the start of reaggregation, hIsMTs were cultured in either standard culture media with 5.5 mM glucose (STD) or media with 11 mM glucose for 7 days. hIsMTs were subsequently used for assaying transduction efficiency, glucose-stimulated insulin secretion, and cellular insulin content.

### Tent5aKO cell line generation

Tent5a knockout (Tent5aKO) INS-1E cells were generated with CRISPR-Cas9. sgRNAs were selected from the Vienna BioCenter (VBC) website and in vitro transcribed with T7 RNA polymerase (NEB) using a PCR product generated with the oligos Fam46a_sg3, T7RevLong, T7FwdAmp, and T7RevAmp as template, as previously described^71,72^. Editing of the INS-1E cells was done as described previously^73^. SpCas9-NLS (100 pmol) was mixed with 120 pmol sgRNA in Cas9 buffer, and the mixture was incubated at 37 °C for 30 min. Approximately 2 × 10^5^ cells were resuspended in 20 µL of nucleofection buffer (Lonza). A Lonza 4D Amaxa nucleofector was used with SF solution and program CM-156. Clones were seeded at an approximate density of 1 cell per 100 µL per well in a 96-well plate and screened first by gel electrophoresis of PCR-amplified genomic region bound by primers rT5A-CrisGeno-F and rT5A-CrisGeno-R. Knockout and wildtype clones were then assessed via Sanger Sequencing and ICE analysis (Synthego) before validation by next-generation sequencing and analysis with CRISPResso^74^.

### RNAi-based screen for functional RBPs in beta cells

siRNAs against 1103 rat RBPs were synthesized as a set of four duplexes for each gene target (Horizon Discovery). The primary screen was performed where one RBP was knocked down in four wells of two identical 384-well plates, and 3–4 pairs of plates were processed within a total of five batches. Control samples were treated with scrambled siRNAs, with one set of control samples per batch. Liquid handling was performed in a semi-automated manner using the AssistPlus automated pipetting system equipped with the Voyager 300 (volumes greater than 10 µL) and Voyager 12.5 (volumes less than 10 µL) multichannel pipettes (Integra). First, 10 µL of EndocC-βH2 coating media, described above, were seeded into 384-well plates, and incubated at 37 °C and 5% CO_2_ for at least 1 h. After removing the coating media, a 10 µL mixture of OptiMEM (Thermo Scientific) containing 1 pmol siRNA and 0.3 µL RNAiMax (Thermo Scientific) was seeded into each well. INS-1E cells were overlayed in a final volume of 50 µL and 2 × 10^4^ cells per well. After cell seeding and between media changes in subsequent steps, plates were centrifuged for 1 min at 100 × *g* to reduce loosely adherent cell loss and supernatant contamination.

At least 48 h after reverse transfection, culture media was replaced with 100 µL of glucose sensitization buffer containing no glucose and 0.1% BSA in KRBH buffer (135 mM NaCl, 3.6 mM KCl, 0.5 mM NaH_2_PO_4_, 0.5 mM MgCl_2_, 1.5 mM CaCl_2_, 5 mM NaHCO_3_, 10 mM HEPES, pH 7.4). After incubation in 37 °C and 5% CO_2_ for 2.5 h, sensitization buffer was replaced with 50 µL of KRBH buffer containing either 3.3 mM glucose (low glucose plate) or 16.7 mM glucose (high glucose plate) for a parallel 30 min glucose-stimulated insulin secretion assay. After stimulation, supernatants were transferred first into temporary 384-well plate before being centrifuged and transferred again as a 30 µL volume into a final 384-well plate for freezing at −20 °C.

Meanwhile, 25 µL of cellular insulin content extraction buffer (0.18 M HCl, 95% ethanol, 0.1% Triton-X-100) was added to each well of the low glucose plate and stored overnight at −20 °C. Cells in the high glucose plate were co-stained for 10 min with the Hoechst (Thermo Scientific) and live-or-dye NucFix^TM^ red viability dye (Biotium) before being fixed with 2% paraformaldehyde (Sigma) and imaged within 30 min. Four images (696 × 696 µm) per well were taken using an ImageXpress Micro high content screening widefield microscope (Molecular Devices) with a 4×/0.2 NA Plan Apo air immersion objective (Nikon) through a Zyla sCMOS camera (Andor) through two channels: Hoechst (violet LED light source and 377/50 nm excitation filter and 447/60 nm emission filter), and live-or-dye (teal LED light source, 513/17 excitation filter and 624/40 emission filter). Viability and cell counts were calculated from image analyses performed on FIJI with custom-made macros that used the “find maxima” function to identify distinct signals. Diluted total cellular insulin (1:100 in KRBH) and undiluted secreted insulin were measured via the homogeneous time-resolved fluorescence high-range insulin measurement kit (Revvity) as per the manufacturer’s protocol.

A subset of siRNAs was manually selected during the secondary screen, and cells were reverse-transfected and incubated in a similar manner as the primary screen. Cells were treated with 3.3 mM glucose and subsequently 3.3 mM glucose and 1 µM glibenclamide (Tocris Bioscience) for 30 min at 37 °C and 5% CO_2_. Secreted insulin was collected and measured as described above.

To perform the glucose-stimulated ATP-generation, Ins-1E cells expressing PercevalHR were reverse-transfected with siRNAs as described above and sensitized with 0 mM KRBH for 2 h prior to imaging live on the ImageXpress Micro microscope with a 20×/0.45 NA Plan Fluor ELWD air immersion objective (Nikon) with settings described previously^69^. Cells were stimulated with either 3.3 mM or 16.7 mM glucose and imaged for 6 min with images taken every 15 s. Signal intensities were analyzed over time on a cellular basis with a custom-made macro on FIJI. The ATP response was assessed via the area under the curve in the increase of the ATP:ADP ratio over time.

### Anisomycin treatment

Islets extracted from four mice, aged 7 weeks, were separated in such a way that approximately 60–80 islets from each mouse were picked into 1 mL islet culture media with anisomycin (0.5 µM; Sigma) and another 60–80 islets were picked into 1 mL of islet media with DMSO (volume-matched; Sigma) shortly after isolation (~3 h). After 24 h, islets were processed for RNA extraction. Insulin content measurements were performed first by separated islets into batches of 20 islets each and treating for 24 h in either DMSO or anisomycin (0.5 µM; Sigma). Islets were then kept in 0 mM KRBH for 1 h at 37 °C and 5% CO_2_ before being dispersed into single cells to get a cell count from 10% of the dispersed cell suspensions. The remaining 90% of the islet cells were centrifuged and resuspended in cellular insulin content extraction buffer.

INS-1E cells were treated with DMSO or anisomycin as described above 48 h after seeding cells. EndoC-βH2 cells were treated with DMSO or anisomycin as described above, 1 week after seeding. In both cases, wells were washed once with 1× PBS, which was then aspirated and replaced with TRIzol for RNA extraction.

### SB 203580 (p38-inhibitor) treatment

INS-1E cells were treated 48 h after seeding were first pre-treated with either SB 203580 (40 µM; Sigma) or DMSO for 1 h at 37 °C and 5% CO_2_. Cells were then supplemented with either DMSO or anisomycin (0.5 µM) and incubated for either 15 min for protein harvesting or 24 h for RNA harvesting.

### Tunicamycin treatment

INS-1E cells were treated 48 h after seeding with either DMSO or 5 µg/mL tunicamycin (5 µg/mL; Sigma) in INS-1E media to induce ER stress for 24 h. Non-adherent cells were harvested from the supernatants, while adherent cells were collected via 5 min incubation with Trypsin/EDTA (0.05%; Thermo Scientific) at 37 °C and 5% CO_2_. Cells were pelleted by centrifugation, washed with 1× PBS before being resuspended in TRIzol for RNA extraction.

### Gluco-lipotoxic/inflammation treatment

INS-1E cells were treated 48 h after seeding with INS-1E media containing 25 mM glucose, 0.4 mM palmitate, 100 ng/mL TNFɑ, 10 ng/mL IL1β, and 5 ng/mL IFNγ or volume-matched PBS-spiked INS-1E media as a control. Cells were treated for 24 h before being harvested in the manner described for tunicamycin treatment.

### Full-length mRNA sequencing (FLAM-seq)

#### Sample preparation and sequencing

FLAM-seq was performed as described previously^35^. Briefly, INS-1E-TetR-Tent5a WT cells seeded on 10-cm culture plates were either induced with doxycycline (10 ng/mL) or not (uninduced control) for 24 h before being harvested via trypsinization. In studies where Ptbp1 or Fndc3a/3b were silenced, INS-1E-TetR-Tent5a WT cells were first reverse transfected with 250 pmol of siRNA against Scrambled control, *Ptbp1,* or *Fndc3a* and *Fndc3b* (Horizon Discovery) 24 h prior to doxycycline treatment. RNA from approximately 2.5 × 10^6^ cells per replicate was harvested via TRIzol-Chloroform extraction. Total RNA (10 µg) was subjected to oligo-d(T)-based poly(A)^+^ RNA capture with the Illumina Stranded mRNA Prep, Ligation kit (Illumina 20040532). Guanidine/inosine (G/I) tailing of the poly(A) RNA was performed using reagents from the Poly(A) Tail-Length Assay Kit (Thermo Scientific; 764551KT), followed by RNA purification using RNAClean XP beads (Beckman Coulter; A63987), following the manufacturer’s protocols. First-strand cDNA synthesis was performed using reagents from the SMART-Seq^TM^ v4 Ultra^TM^ Low Input RNA kit for sequencing (Takara; 634896) using the universal reverse transcription primer PS-FSRT-Rev-1 (Microsynth) and template-switching oligo, PS-isoTSO (IDT DNA Technologies), followed by DNA purification using AMPure XP DNA beads (Beckman Coulter; A63880). PCR amplification was performed with reagents from the SMART-Seq^TM^ v4 Ultra^TM^ Low Input RNA kit and Advantage® 2 PCR Kit (Takara; 639207) using the forward primer 5′ PCR Primer II A and the reverse primer PS-Amp-R1. The amplified libraries were again purified with the AMPure XP DNA beads and submitted for further library barcoding and preparation with the SMRT bell library preparation using 700 ng of double-stranded cDNA per sample at the Next Generation Sequencing Platform at the University of Bern. The libraries were sequenced with the PacBio Sequel IIe on one SMRT cell 8 M (for Tent5a WT analysis) or with the PacBio Revio system on one SMRT cell 25 M (for Tent5aKO, *Ptbp1,* and *Fndc3a/b* silencing. Data were processed through the Iso-Seq pipeline.

### Data analysis

Long reads generated in this study were deposited at NCBI Sequence Read Archive (SRA, www.ncbi.nlm.nih.gov/sra) and are accessible through the accession PRJNA1267696. Reads were processed with the FLAM-seq pipeline available on github.com/rajewsky-lab/FLAMAnalysis^35^. As reference genome, the rat genome available on ensembl.org (Ensembl release 109, mRatBN7.2) was used, and the average tail lengths were extracted. Data for genes with less than three reads was set to NA, and only genes with data available for at least two out of three replicates per group were kept for further analyses.

Distances from the polyadenylation sites to the transcriptional termination sites (TTS) were calculated using the canonical Ensembl transcripts (https://www.ensembl.org/info/genome/genebuild/canonical.html). TTS were extracted with the script parseGTF.pl from Homer (version 4.11)^75^. Genomic positions of the reads assigned to genes by the FLAM-seq pipeline (mapQuantGeneDir/sampleID_Aligned.sortedByCoord.out.bam.featureCounts and cleanGenomicDir/sampleID_cleaned.bam) were extracted as well and matched to the transcription termination site (TTS) with bedtools (version 2.26.0, bedtool closest with the option -S to only match opposite strands^76^). Distances were calculated as such that a positive value indicates a transcript longer than annotated. Distances with an absolute value larger than 10 kb were set to NA (about 4% of all distances). Distances were Log_10_(*x* + 1) transformed while preserving the sign and finally averaged for each gene. The number of reads surviving the distance filter were also summed up for each gene. Data for genes with less than three reads was set to NA, and only genes with data available for at least two out of three replicates per group were kept for further analyses. To test for differences in tail length or distance of the polyadenylation site to the TTS, we use a one-way ANOVA that models tail length in response to the experimental group. We first tested for differences in the average tail length and then for each gene individually. To identify genes associated with secreted proteins, a list of rat transcriptome entries with the “SignalP-noTM” designation, based on established hidden Markov model predictions for signal peptides, were exported from Ensembl BioMart and matched to the FLAM-seq dataset^77^.

### RNA sequencing

RNA sequencing was performed as previously described^78^. RNA isolated from islets from each mouse was TRIzol-chloroform extracted. INS-1E cells seeded with 5 × 10^5^ cells on 10-cm culture plates were cultured for five days prior to dox induction for 24 h to express Tent5a WT or Tent5a M1 or left uninduced (as a control) prior to RNA isolation via TRIzol-chloroform extraction. RNA was DNase (Thermo Scientific) treated for 30 min at 37 °C prior to library preparation performed at the Functional Genomics Center Zürich (FGCZ) using the TruSeq Stranded mRNA Library Prep Kit (Illumina). Sequencing was performed on an Illumina Novaseq 6000 with single read 100 bp configuration. Reads generated in this study were deposited at NCBI Sequence Read Archive (SRA, www.ncbi.nlm.nih.gov/sra) and are accessible through the accession number PRJNA1267696. Raw reads were prepared by removing adaptors and trimming ends and low-quality reads (phred quality <20) and then pseudo aligned to the rat reference genome (Ensembl Rnor_6.0) using Kallisto version 46^79^. Differential expression and over-representation analyses were performed with DESeq2, where *p*-values calculated with the Wald test and adjusted for multiple comparison using the Benjamini–Hochberg method^80^.

Previously published differential expression analysis of islets from *Ins2*^*Akita*^ mice were filtered and presented in this study to identify changes to RBPs only^28^.

### Northern blot

Northern blotting was performed as described previously^81^. TRIzol-chloroform-extracted RNA (10 µg) was heated in loading buffer (1× MOPS-EDTA-sodium acetate buffer, 20% glycerol, 6.5% formaldehyde, 50% formamide, and 0.05% bromophenol blue) and run in a 1.5% agarose/6.4% formaldehyde gel in 1× MOPS buffer (20 mM MOPS, 80 mM sodium acetate, and 1 mM EDTA). Separated RNA was transferred overnight onto Hybond N^+^ nylon membranes (Amersham) with 10× SSC. PCR-amplified rat *Ins1/2* coding domain sequence probes (primers PS-rIns-5-F and PS-r-Ins-pA-R4) were labeled with ^32^P dCTP via nick translation (Thermo Scientific) and purified over nucleotide purification G-50 columns (General Electric Healthcare) prior to hybridization. Membranes were prehybridized at 50 °C with rotation for 3 h prior to 16 h of hybridization with the probe in hybridization buffer (50% formamide, 6× SSC, 5× Denhardt’s reagent, 5 mM EDTA, 0.2% SDS, and 100 µg/mL salmon sperm DNA). Membranes were washed twice with buffer A (2× SSC, 0.1% SDS, 1 mM EDTA) at 37 °C for 10 min each, followed by two washes with buffer B (0.2× SSC, 0.1% SDS, 1 mM EDTA) at 60 °C for 20 min each. Blots were developed on phosphorImager screens for 16 h and developed on a Typhoon imager (Cytiva). Lane densitometries were analyzed in FIJI and normalized to the maximal signal per lane.

### Re-analysis of human islet RNA-seq data

Data were obtained from Gene Expression Omnibus under the accession number GSE164416 as published previously^30^. Briefly, RNA-seq data was processed as described above, and the transcript expression between *INS* (ENSG00000254647) and *TENT5C* (ENSG00000183508) expressed as Log_2_(*x* + 1) were plotted for each donor classified as nondiabetic, impaired glucose tolerance, and T2D.

### Gene expression analysis

Gene expression analysis via quantitative PCR (qPCR) was performed as previously described^78^. Briefly, TRIzol-chloroform extracted RNA was normalized as 1–2 µg and then DNase I (Thermo Scientific) treated to remove genomic DNA. cDNA libraries were generated using the High-Capacity cDNA Reverse Transcription Kit (Thermo Scientific) according to the manufacturer’s protocol. qPCR reactions were performed with SYBR green (KAPA Biosystems) as per the manufacturer’s protocol. Relative expression was performed using the ∆∆C_t_ method, where gene expression was normalized to *36b4* (*Rplp0*) or Tata-binding protein (*TBP*) as indicated. Primers used in this study for qPCR are listed in Supplementary Data 8.

### mRNA half-life study with actinomycin D treatment

INS-1E cells were seeded at a density of 1.5 × 10^5^ cells per well. In the case of the inducible lines, cells were either left uninduced or induced with doxycycline (10 ng/mL) to express either Tent5a WT or Tent5a M1 for 48 h before being treated with Actinomycin D (5 µg/mL; Sigma) for 24, 16, 8, or 4 h. Both adherent and nonadherent cells were pooled for RNA extraction via TRIzol-chloroform. RNA was reverse transcribed, and gene expression was assessed by qPCR relative to samples untreated with actinomycin D (i.e., 0 h) as previously described^82^. mRNA half-life was estimated by fitting data to an exponential growth function on Prism (GraphPad).

### Polysome profiling and RNA seq

Cells were seeded 24 h prior to induction (or not) with doxycycline (10 ng/mL) for 24 h. Cells were incubated with INS-1E culture media containing cycloheximide (100 µg/mL; Sigma) for 10 min at 37 °C for 10 min prior to harvesting with trypsin/EDTA solution and washing once with ice-cold 1× PBS, where both solutions contained 100 µg/mL cycloheximide. For each sample, 10% of the harvested cells was TRIzol-chloroform extracted as inputs. The remaining cell pellets, numbering, on average, 2 × 10^7^ cells per sample, were snap frozen. Pellets were disrupted in lysis buffer (20 mM Tris pH 7.4, 140 mM KCl, 5 mM MgCl_2_, 1% Triton-X-100, 25 U/mL Turbo DNAse I (Roche), 1 mg/mL Heparin, SUPERaseIn RNase inhibitor (Ambion), 1 mM DTT, 100 µg/mL cycloheximide, protease inhibitor (Roche)). Lysates were centrifuged at 20,000 × *g* for 20 min at 4 °C. Supernatant containing 180 µg RNA in 120 µL was loaded onto a 20–60% sucrose gradient prepared in buffer (20 mM Tris pH 7.4, 140 mM KCl, 5 mM MgCl_2_, 1 mM DTT, and 100 µg/mL cycloheximide). Gradients were fractionated at 247,600 × *g* with SW 41 Ti (Beckman Coulter) rotor for 3.5 h, at 4 °C. Fractionated ribosomes were monitored and collected using the Density Gradient Fraction System (BioComp) in 700 µL fraction volumes with a scan speed of 0.2 mm/s, 260 nm wavelength excitation, and 500 ms integration time. RNA from 300 µL of fractions 11 to 15 were harvested by TRIzol-chloroform and pooled to represent the polysome fraction. Input RNA and polysome RNA were poly(A) enriched prior to library preparation as described above and sequenced on the NovaSeq X Plus (Illumina) through Novogene as 150 bp pair-end reads. Reads were cleaned from adapters and quality-trimmed with fastp (version 0.23.4^83^). Reads were aligned to the rat transcriptome (Ensembl release 109, mRatBN7.2) with salmon (version 1.4.0^84^). Variation in gene expression was analyzed with a general linear model in R with the package DESeq (version 1.24.0^80^) according to a crossed factorial design with two explanatory factors TYPE (input or polysome) and TREAT (control or Tent5a overexpression). Genes differentially expressed between specific conditions were identified with linear contrasts and reported as log_2_(*x* + 1). Translation efficiency was defined as the ratio of expression in polysomes to input for each gene.

### Fluorescence microscopy

#### Sample preparation

Approximately 1 × 10^5^ INS-1E cells were seeded into 8-well chamber slides (Ibidi) per well 24 h before doxycycline induction to express Tent5a WT or Tent5a M1 or left uninduced for 48 h.

For assessment of HA-tagged Tent5a construct cellular distribution, samples were fixed with paraformaldehyde (2% in 1× PBS; Sigma), permeabilized for 10 min with Triton-X-100 (0.1%; Sigma) before blocking with bovine serum albumin (1%). Samples were then incubated at RT for 2 h, with either rabbit monoclonal antibody targeting the HA-tag (0.66 µg/mL; Cell Signaling Technology) or rabbit IgG isotype control (0.66 µg/mL; Abcam), before being washed and co-stained with Alexa Fluor 568 goat-anti rabbit IgG (1:500; Thermo Scientific) and Hoechst (1:2000; Thermo Scientific) for 30 min at RT.

For ER morphology assessment, cells were treated as described above but with digitonin permeabilization (12.5 µg/mL; Sigma) for 10 min, stained with a polyclonal rabbit anti-calreticulin antibody (1:300; Sigma), incubated overnight at 4 °C, and labeled with Cy5-conjugated goat anti-rabbit IgG secondary antibody (1:500; Thermo Scientific).

For colocalization studies, cells constitutively expressing the ER-localized ERroGFP construct were first reverse transfected with 2.5 pmol of scrambled siRNA or siRNAs against Fndc3a, Fndc3b, or an equal portion of both 24 h prior to doxycycline (10 ng/mL) treatment to induce expression of HA-tagged Tent5a WT for an additional 24 h. The HA-tag was labeled first with the primary rabbit monoclonal anti-HA-tag antibody (1:200; Cell Signaling Technology) for 2 h and labeled with a Cy5-conjugated goat anti-rabbit IgG antibody (1:500; Thermo Scientific). Samples were washed four times with 1× PBS supplemented with BSA (0.1%) prior to boosting the ER label using an Alexa Fluor 488-conjugated anti-GFP antibody (1:500; Thermo Scientific) and nuclei staining via Hoechst (1:2000).

To measure ER-mitochondria proximity, cells were first reverse-transfected with Lipofectamine^TM^ 2000 (1:1 ratio; Thermo Scientific) and 500 ng plasmid DNA encoding the FRET-based indicator of ER-mitochondria proximity (FEMP) sensor following the manufacturer’s protocol. Cells were either uninduced or induced with doxycycline (10 ng/mL) to express Tent5a WT or Tent5a M1 for 48 h prior to live cell imaging.

### Confocal microscopy

Images were collected on a Leica TCS SP8 using a 63×/1.4 NA oil HC PL APO CS2 objective. The following laser lines were used to excite the following fluorophores 405 nm (Hoechst, Cyan fluorescent protein, or the yellow fluorescent protein for FRET), 488 nm (Alexa Fluor 488, GFP), 561 nm (Alexa Fluor 568), and 633 nm (Cy5). Bandpass filters for each fluorophore were set for the HyD or PMT detectors, ≥10 nm away from excitation lines, and ranges set to minimize overlap with other fluorophores. Acquisition was performed with either sequential frames or line scans with separate channels per fluorophore. Images for HA-tag distribution and FEMP sensors were taken at a single z-plane, while Images for ER and mitochondrial morphology and colocalization analyses collected as z-stacks.

### Analysis

Image analysis was performed on a Fiji microscope with custom macros. Z-stack images were deconvolved using the DeconvolutionLab2 plugin with the Richardson–Lucy algorithm (*N* = 10 iterations)^85^. Manual cell selection defined the total cell area, and the total cytoplasmic area was identified as the subtraction between this region of interest (ROI) and Li-thresholded Hoechst signal. For ER footprint comparison, the Z-stack was collapsed as a maximum project and the calreticulin signal was Otsu-thresholded to identify the ER area. Colocalization analysis was performed using the “colocalization threshold” plugin by comparing the HA-tag channel against the eroGFP-labeled ER stain to generate cell-based Pearson correlation coefficients. Line profiles of arbitrary 16 µm-long paths across cells, where the fluorescence intensity was normalized against the signal maxima as previously described^86–88^.

Cellular FRET efficiency was calculated as the ratio of the background-corrected FRET to the background-corrected signal of the cyan fluorescent protein (acceptor) as previously described^56^.

### Luciferase assays

Approximately 5 × 10^4^ INS-1E TetR-Tent5a WT cells were reverse transfected with 200 ng each of both pRL-SV40-Renilla (Promega) and pGL410_INS421 (Addgene #49057) with 1:1 ratio of Lipofectamine^®^ 2000 (Thermo Scientific) in µClear^®^ CELLSTAR^®^ white wall 96-well plates. For glucose responses, cells were cultured concurrent with transfection in INS-1E media containing either 1, 11, or 25 mM glucose. For assessment during Tent5a WT overexpression, cells were either induced with 10 ng/mL doxycycline or left uninduced, concurrent with transfection. Twenty-four hours post-transfection, cells were lysed on-plate and luciferase assay was performed with the Dual-Luciferase^®^ Reporter Assay System (Promega) following the manufacturer’s protocol. Luminosity was measured on a Tecan M1000 Plex plate reader.

### Human islet microtissue assays

On day 16 of culture, AAV-GFP infected hIsMTs were prepared for imaging as previously described^67^. hIsMts were prepared by first washing twice with 1× PBS (containing Mg^2+^ and Ca^2+^), fixed for 15 min in 4% PFA before washing twice again with 1× PBS, and stored in PBS with 0.05% sodium azide prior to staining. hIsMTs were first permeabilized with 0.5% Triton-X-100 in PBS (subsequently without Mg^2+^ or Ca^2+^) before blocking with 5% normal donkey serum. hIsMTs were incubated overnight with primary antibody in antibody dilution buffer (5% donkey serum, 0.2% Triton-X-100 in PBS): rabbit anti-NKX6.1 (Abcam, ab221549) and sheep anti-ARX (R&D Systems AF7068-SP). hIsMTs were next washed with 0.2% Triton-X-100 in PBS and incubated with secondary antibody in a dilution buffer with DAPI: donkey anti-rabbit Alexa Fluor 568 (Thermo Scientific, A100042) and donkey anti-sheep Alexa Fluor 647 (Jackson ImmunoResearch, 713-605-147). After washing, hIsMTs were transferred into an Akura 384-well ImagePro plate (InSphero) and cleared using ScaleS4 solution for Z-stack fluorescence imaging (including GFP) every 3 µm on a Yokogawa CQ1 Benchtop High-Content Analysis System^89^. Image quantification was performed with CellPathFinder custom pipelines.

hIsMTs were subjected to sequential glucose-stimulated insulin secretion assays in 50 µL KRBH media containing first 2.8 mM followed by 16.7 mM glucose for 2 h each. Cellular insulin content was extracted from 40 µL of hIsMT lysates. Insulin ELISAs were performed on a per-MT basis (STELLUX® Chemi Human Insulin ELISA, Alpco, 80-INSHU-CH01).

Pools of 12 hIsMTs were washed with 1× PBS before being snap-frozen in liquid nitrogen and used for RNA isolation using the PicoPure RNA extraction kit (Thermo Scientific) with DNase treatment (Qiagen). RNA was subsequently used for reverse transcription and qPCR as described above.

### Cellular PDI activity assay

Cells were induced with doxycycline (10 ng/mL) or left uninduced for 48 h prior to harvesting and normalizing to 1 × 10^6^ cells per sample. Samples were then processed using the protein disulfide isomerase (PDI) activity fluorometric assay (Abcam) according to the manufacturer’s protocol^77^. Change in fluorescence measurement over 2 min used to report the activity of cellular PDI.

### Cellular ATP assay

Cellular ATP levels were measured as previously described^69^. Cells were induced with doxycycline (10 ng/mL) for the indicated incubation times prior to trypsinization and reseeding into 384-well plates at a density of 5 × 10^3^ cells per well. Cells were immediately processed with the Cell Titer-Glo 2.0 Reagent (Promega). ATP levels were calculated against a standard curve prepared with an ATP standard stock (Thermo Scientific) using the luminescence signal generated via the Cell Titer-Glo 2.0 Reagent as per the manufacturer’s protocol.

### Immunoprecipitation and proteomic analysis

Samples were prepared for immunoprecipitation and proteomic analysis as previously described^81^. Cells were either left uninduced or induced for 48 h to express Tent5a WT or Tent5a M1 after seeing 4 × 10^6^ cells each onto two 15-cm culture dishes per sample. Cells were washed off the culture plate with 1× PBS, pelleted by centrifugation, and resuspended in low salt lysis buffer (20 mM Tris-HCl, pH 7.4, 1% NP-40, 135 mM NaCl, 2 mM EDTA) containing protease and phosphatase inhibitor (Roche). Samples were normalized to 2 mg total protein in 700 µL and immunoprecipitated overnight at 4 °C with 30 µL magnetic Dynabeads (Thermo Scientific) conjugated to 0.5 µg rabbit monoclonal anti-HA-tag antibody (Cell Signaling Technology). The immunoprecipitated beads were washed once with low salt buffer before being further processed for proteomic analysis with the Functional Genomic Center Zürich. Peptides were digested on-bead with sequencing-grade trypsin and subjected to liquid chromatography-mass spectrometry (LC-MS) using label-free high-resolution accurate mass data acquisition on a nanoflow LC-MS system with data-dependent acquisition logic. Four biological samples for the Tent5a WT, Tent5a M1, and uninduced control groups were analyzed. Interaction between potential prey proteins and bait protein was evaluated with the SAINTexpress software, where interactions were scored based on the empirical fold-change and Bayesian FDR^90^. The raw mass spectrometry data has been deposited in the ProteomeXchange under the accession number PXD066929.

Comparison of Tent5a WT with either Tent5a-∆N and Tent5a-∆C was performed in the presence of 1 µM of the proteasome inhibitor MG-132 (Sigma) with doxycycline induction for 6 h, to maintain a higher expression of the Tent5a-∆N and -∆C constructs, which had lower expression levels compared to Tent5a WT. The input consisted of 6 × 10^6^ Tent5a WT (both induced and uninduced), 12 × 10^6^ Tent5a-∆C, and 16 × 10^6^ Tent5a-∆N expressing cells were used as inputs.

Immunoprecipitation experiments with PTBP1 were performed by coupling 1.67 µg mouse monoclonal anti-Ptbp1 antibody (Sigma) to 10 µL of Dynabeads for overnight immunoprecipitation with 2 × 10^6^ cells, uninduced or Tent5a WT-induced cells, or 4 × 10^6^ Tent5a-∆C induced cells.

### Western blotting

Cells were collected via centrifugation 100 × *g* for 3 min and washed with 1× PBS. Cell pellets were resuspended in RIPA buffer (10 mM Tris-HCl, pH 8.0, 1 mM EDTA, 0.5 mM EGTA, 1% Triton-X-100, 0.1% sodium deoxycholate, 0.1% SDS, 140 mM NaCl) supplemented with protease inhibitor cocktail and phosphatase inhibitor (Roche) according to the manufacturer’s instructions. Cells were lysed for 30 min on ice before the insoluble fraction was separated by centrifugation at 16,100 × *g* for 10 min at 4 °C. Protein concentrations were assessed via bicinchoninic acid (BCA) assay. Total protein lysate (20–40 µg) was mixed with Laemmli buffer, boiled for 5 min, and loaded into 10% SDS-PAGE gels for protein separation. Proteins were transferred to nitrocellulose membranes via semi-dry or wet transfer methods. Membranes were blocked in 5% milk/TBST or 5% BSA/TBST (in the case of antibodies against phospho-protein) for 1 h. Primary antibody incubation occurred overnight at 4 °C. After washing, membranes were exposed to horseradish peroxidase (HRP) conjugated anti-rabbit or anti-mouse IgG antibodies for 45 min at RT. After washing, membranes were exposed to chemiluminescence solution (100 mM Tris, pH 8.6, 250 µg/mL luminol, 110 µg/mL p-coumaric acid, 10% DMSO, 0.1% H_2_O_2_). Chemiluminescence signal was acquired on a LAS 4000 imager (Fujifilm). Uncropped scans of Western blots are available in Source data files. Antibodies used for protein detection are provided in Supplementary Data 9. Densitometry was performed using the built-in gel analysis tool on Fiji to calculate the area under the curve (AUC) of the band of interest bound within the same fixed-width lane profile. Values were reported as fold-changes in the AUC ratio of antigen band to housekeeping antigen band over the average for control replicates.

### Insulin processing western immunoblotting

To immunoblot insulin, the following modifications were applied as described previously^78^. Cells were either uninduced or induced for 48 h with doxycycline (10 ng/mL) to induce Tent5a WT or Tent5a M1. Cells were collected via trypsinization and normalized to 2 × 10^6^ cells per sample, which were washed with 1× PBS and resuspended in SDS lysis buffer (20 mM Tris-HCl, pH 7.5, 1% SDS) supplemented with protease inhibitor cocktail. Lysed cells were sonicated at a low intensity five times (30 s on and off each) with a Bioruptor^®^ Plus (Diagenode) and clarified via centrifugation. Protein was normalized to 50 µg in SDS lysis buffer and heated at 70 °C for 10 min with loading buffer (37.5 mM Tris-HCl, pH 7.5, 3% SDS, 1.5% beta-mercaptoethanol, 7.5% glycerol, trace Coomasie Blue G250). Lysates were run overnight at 4 °C on a gradient 16–10–4% polyacrylamide gels at 25 V in tricine-based anode buffer (100 mM Tris pH 8.25, 100 mM Tricine, 0.05% SDS) and cathode buffer (100 mM Tris pH 8.9, 22.5 mM HCl). Separated proteins were transferred to nitrocellulose membranes via wet transfer, blocked initially for 5 min at RT with 1% milk/1% BSA/TBST before being fixed for 15 min with 0.2% glutaraldehyde solution in 1× PBS supplemented with 0.1% Tween. Blocking was continued for 30 min before incubation with a rabbit polyclonal anti-insulin antibody (Cell Signaling Technology; 1:1000). The remaining steps followed the western immunoblotting protocol described above.

### Cellular fractionation

Cellular fractionation was performed as previously described^81^. INS-1E cells treated with doxycycline (10 ng/mL) to induce Tent5a WT or Tent5a M1 were harvested by trypsinization and normalized as 1 × 10^6^ cells per sample, where 3% of each of the three replicates was pooled to prepare whole cell lysates via RIPA-buffer extraction. The remaining cells were pelleted and incubated with 150 µL hypotonic lysis buffer (HLB; 10 mM HEPES, 1.5 mM MgCl_2_, 10 mM KCl, 1 mM DTT with protease inhibitor). After 30 min on ice, 6 µL of 10% NP-40 was added to the suspension, and samples were centrifuged at 16,100 × *g* for 35 s at 4 °C. The supernatant (cytosolic extract) was separated from the nuclei, which were washed once with HLB before being extracted with 30 µL nuclear lysis buffer (NLB; 10 mM HEPES, 100 mM KCl, 3 mM MgCl_2_, 0.1 mM EDTA, 1 mM DTT with protease inhibitor). After 30-min incubation on ice, 3 µL of 4 M ammonium sulfate was added, and samples were centrifuged at 16,100 × *g* for 15 min at 4 °C.

Whole cell lysate and cytosolic fractions were normalized to 10 µg, while nuclear fractions were normalized to 5 µg in their respective lysis buffers before being processed for Western blotting as described above.

### In vitro mRNA decay assay

mRNAs were first produced with *BsmI*-digested Ins1-Flag-A32 and Ins1-Flag-A159 plasmids as a template for in vitro transcription with the MEGAscript^TM^ T7 Transcription kit (Thermo Scientific) following the manufacturer’s protocol. mRNA was purified with LiCl precipitation followed by quality assessment and quantification with gel electrophoresis on the TapeStation system (Agilent). Cytoplasmic fractions of 5 × 10^5^ cells were isolated in 50 µL hypotonic lysis buffer without DTT (as described above) and further diluted 1:1000 (to a final volume of 400 µL) and spiked with 16 pmol of either *Ins1-FLAG-A*_*32*_ *or Ins1-FLAG-A*_*159*_. Samples were incubated over the course of 60 min at 37 °C, during which time, 8 µL (2%) of the reaction volume was transferred to 1 mL TRIzol at the indicated time points for RNA isolation as described above. Isolated RNA was resuspended in 20 µL, where 4 µL were used for cDNA synthesis, which was subsequently diluted 1:200 to assess mRNA abundance in the reaction via qPCR.

### Cell death assay

Adherent cells treated with DMSO, tunicamycin, or anisomycin were trypsinized and pooled into V-bottom wells of 96-well plates, where they were first stained with Helix Blue (1:500 in 1× PBS; BioLegend) for 5 min followed by staining for 15 min with Annexin V conjugated to APC (1:500; BioLegend) in Annexin V staining buffer (10 mM HEPES pH 7.5, 140 mM NaCl, 2.5 mM CaCl_2_). Cells were centrifuged at 129 × *g* for 2 min before being resuspended in Annexin V staining buffer and processed on a Fortessa flow cytometer (BD Bioscience) in high-throughput screening mode. FACS plots were analyzed on Flowjo, where the proportion of dead cells was marked as the percentage of high Annexin V signal cells.

### AlphaFold modeling

The complexes formed by Tent5a with Fndc3b in the presence of insulin mRNA transcripts and Ptbp1 were predicted using the online AlphaFold3 server (https://alphafoldserver.com). The models used to characterize the catalytic center of mouse Tent5a in complex with one adenosine triphosphate molecule and the 3′ end of *Ins2* poly(A) tail, and the interaction between mouse Tent5a and rat Fndc3b were derived from the same prediction, resulting in ipTM = 0.43 and pTM = 0.41. The prediction with mouse Tent5a in complex with rat Ins1 mRNA, Fndc3b, and Ptbp1 yielded a model with ipTM = 0.31 and pTM = 0.32.

### Statistics and reproducibility

Statistical parameters, including the exact *n* values, precision measures (mean ± SD), and statistical significance are reported in the figures and figure legends. Two-sided unpaired Student’s *t* test was applied for comparisons between two groups. ANOVA was used on comparisons that involved multiple groups. When comparing X–Y data, was assessed via nonparametric Spearman correlation with coefficients (*R*_s_) and *p*-values reported where appropriate. Proportional comparisons were assessed for statistical differences via binomial testing. *p* < 0.05 was considered significant. Line profiles of Northern blot lanes were fit to Gaussian models, and means were compared with extra sum-of-squares F-test to generate *p*-values. Half-life values were taken from the parameter k when data was fit to exponential growth curves, and means were compared with extra sum-of-squares F-test to generate *p*-values. Western blots of immunoprecipitation experiments are representative of at least two independent experiments. Samples were not systematically blinded before every experiment. Biochemical experiments performed in this study are representative of at least two independent experiments. All replicates (*n*) indicated in the figure legends are biological unless stated otherwise. No statistical methods were used to predetermine sample size. Sample sizes are similar to those generally employed in the field. Statistical analyses were performed using Prism 10 (Graphpad). Figures were prepared with Adobe Illustrator (Adobe).

### Reporting summary

Further information on research design is available in the Nature Portfolio Reporting Summary linked to this article.

## Supplementary information

Supplementary Information

Description of Additional Supplementary Files

Supplementary Tables 1–6

Supplementary Table 7

Supplementary Table 8

Supplementary Table 9

Reporting Summary

Transparent Peer Review File

## Source data

Source Data

## Data Availability

The long- and short-read RNAseq data generated in this study have been deposited in the NCBI Sequence Read Archive (SRA, www.ncbi.nlm.nih.gov/sra) and are accessible through the accession PRJNA1267696. The raw mass spectrometry data has been deposited in the ProteomeXchange under the accession number PXD066929. Source data are provided with this paper.

## Code availability

ImageJ macros for image analyses are provided in the following link: 10.5281/zenodo.19437873
