# Supplementary material for: Polyadenylation of insulin mRNA by Tent5a regulates pancreatic beta cells
Source: Nat Commun. 2026 May 20;17:7295. doi: 10.1038/s41467-026-72905-8 (PMC13402609; doi:10.1038/s41467-026-72905-8)
Supplement: Supplementary file 1 — Supplementary Information [file 41467_2026_72905_MOESM1_ESM.pdf]

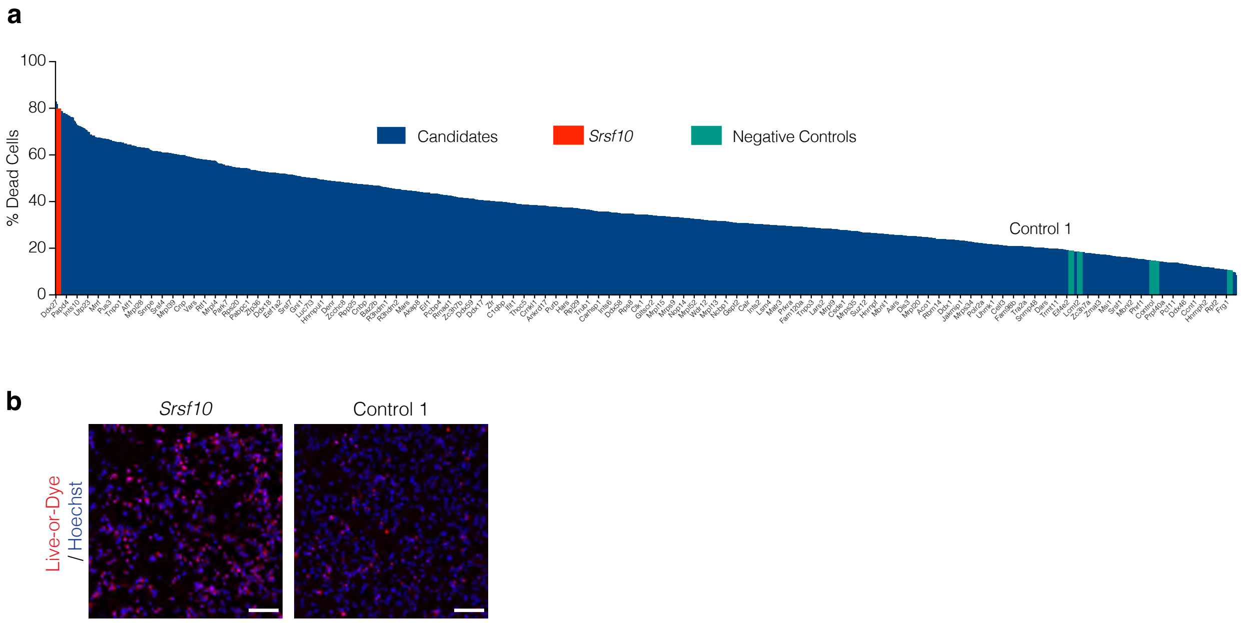


**Figure S1. RNAi screen identifies novel RBPS regulating beta cell function.** (**a**) Effect of RBP KDs on cell viability. Bars represent the average of biological replicates. (**b**) Representative images of Srsf10 KD and Control 1 highlighting Hoechst (*blue*) or viability stain, Live-or-Dye (*red*). Scale bar: 20 μm.

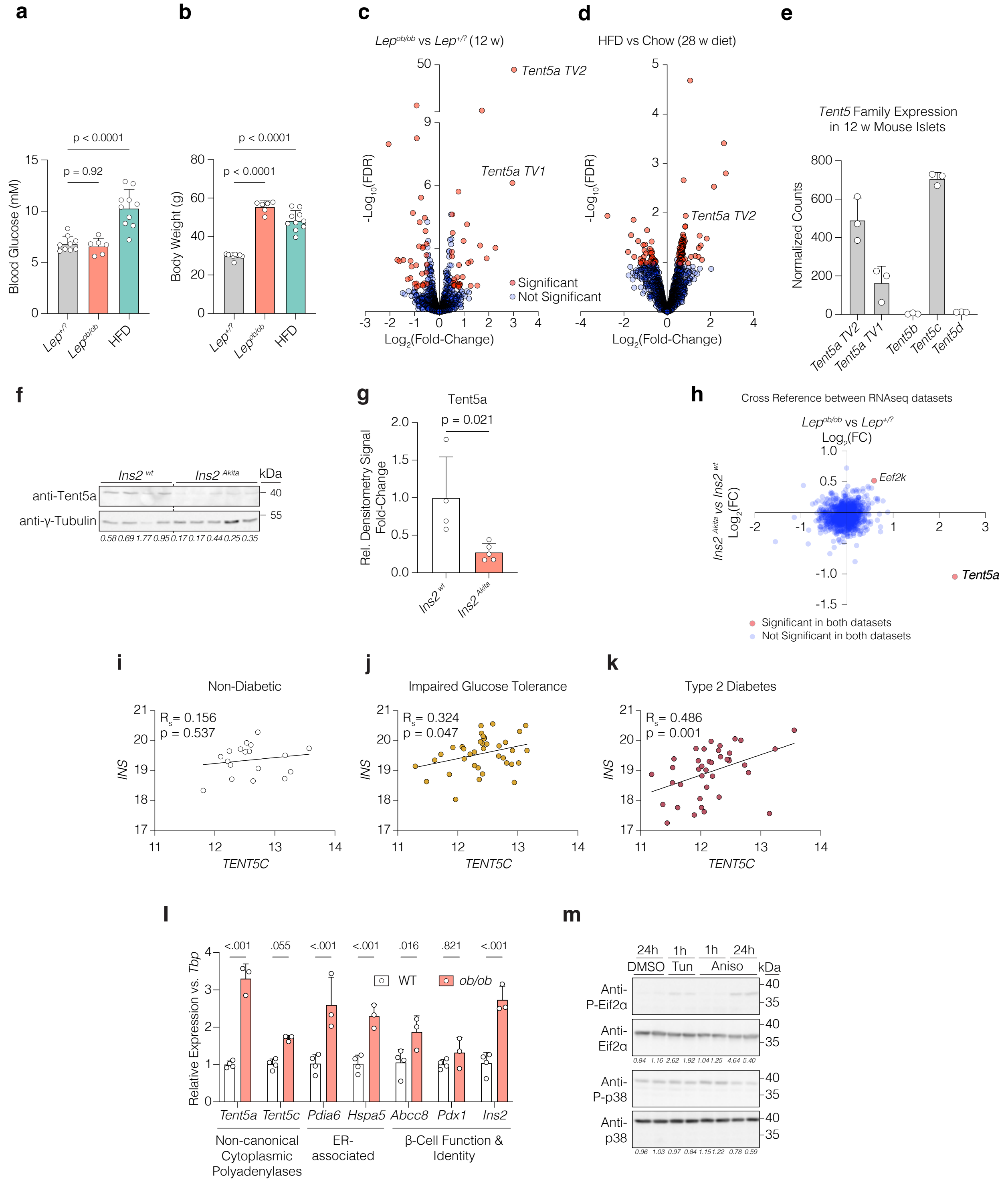


**Figure S2. Tent5a is a highly regulated RBP gene induced during beta cell compensation and translational stress.** (**a**) Blood glucose and (**b**) body weight of control (*Lep*$^{+/?}$), *Lep*$^{ob/ob}$ and HFD mice. (**c,d**) Differential expression analysis (DEA) of RBP genes from islet transcriptomes of (**c**) 12 week-old *Lep*$^{ob/ob}$ vs *Lep*$^{+/?}$ controls or (**d**) islets from C57Bl/6 mice maintained for 28 weeks on a 45% fat/17% sucrose (HFD) or chow diet. Significant (*red*) or non-significant (*blue*) transcripts were identified based on a $Log_2$(fold-change) cut-off of 0.5 and FDR value of 0.05. (**e**) *Tent5* expression levels from RNA-seq data of 12 week-old mouse pancreatic islets. n=3. (**f**) Western blotting of Tent5a and γ-tubulin and (**g**) densitometry of islets from 13 week-old *Ins2*$^{Akita}$ (n=5) and WT (Ins2 wt; n=4) mice. (**h**) RBP transcripts' RNA-seq-derived $Log_2$(FC) of 6 week-old *Lep*$^{ob/ob}$ (x-axis) and 3 week-old *Ins2*$^{Akita}$ against their respective wildtype controls. Presence (*red*) or absence (*blue*) of statistical significance in both datasets was determined from DEAs represented in Figs. 2a and 2d. (**i-k**) Correlation analyses representing expression levels of *INS* and *TENT5C* of individual human islet donors segregated based on clinical diagnoses of (**i**) non-diabetic (n=18), (**j**) impaired glucose tolerance (n=38), and (**k**) type 2 diabetes (n=39). Lines of best fit, Spearman correlation coefficients ($R_s$) and accompany p-values provided. (**l**) Expression analysis of *Tent5c*, ER-associated, and beta cell identity genes measured by qPCR from *Lep*$^{ob/ob}$ (n=3 mice) or *Lep*$^{+/?}$ control (n=4 mice) islets. (**m**) Western blot of INS-1E cells treated with tunicamycin or anisomycin for the indicated time and probed for phospho-, total Eif2α, or p38. n=3 samples for each condition. Data are presented as mean ± s.d. in a–b, e, g and l. One-Way ANOVA followed by Dunnett's multiple comparison testing used to analyze a and b. Two-sided unpaired t-test used to analyze data in g. Two-Way ANOVA with Šídák multiple comparison testing used to analyze l.

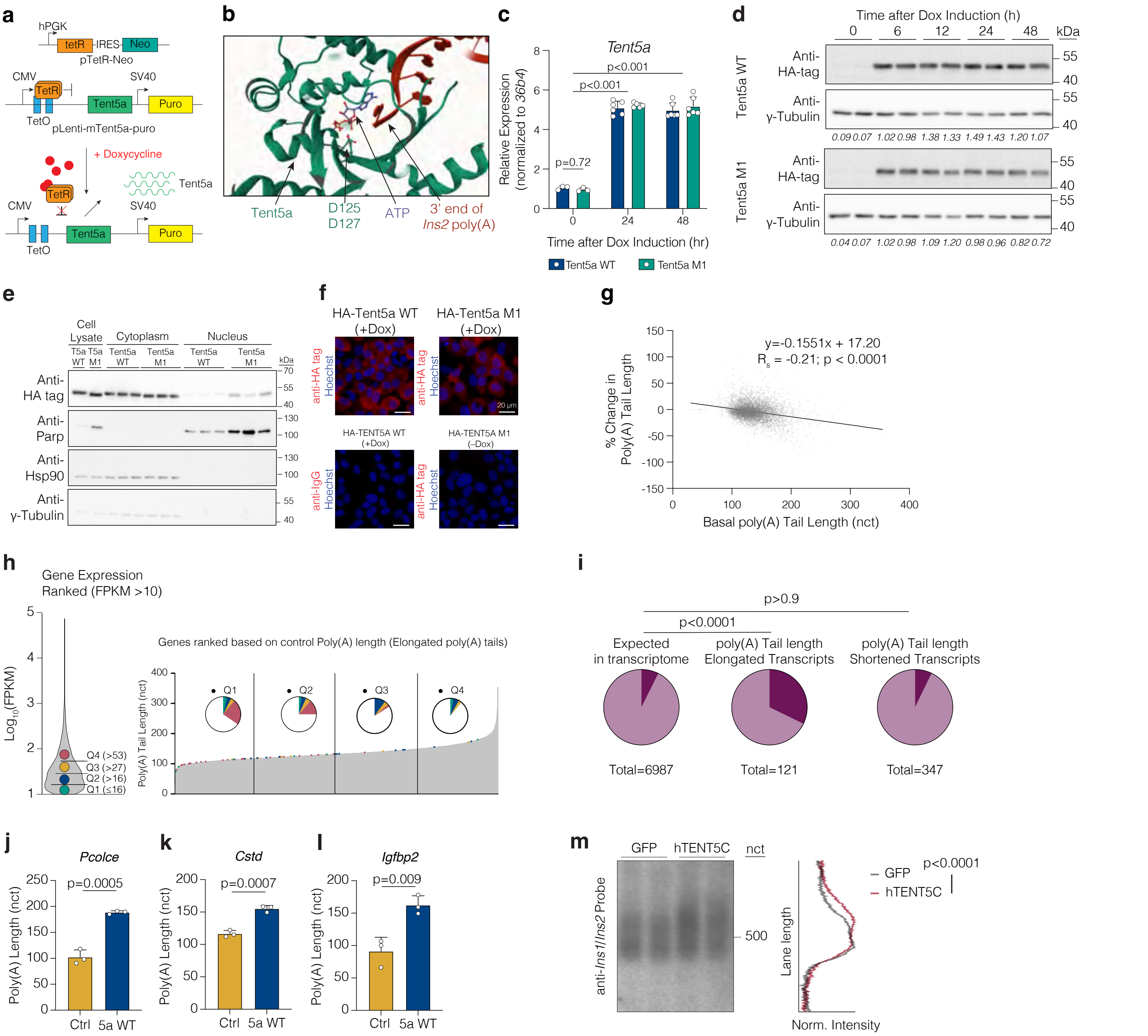


**Figure S3. Tent5a activity elongates poly(A) tails of Ins mRNA.** (**a**) Schematic of doxycycline-inducible expression of Tent5a in INS-1E cells. (**b**) AlphaFold3 model of Tent5a showing the catalytic center, including conserved residues in the wildtype TENT5A and ATP, and D125/127 (Gly in the catalytically inactive M1 mutant). (**c**) Relative Tent5a expression in Tent5a WT (*blue*) and M1 (*teal*) cell lines 24- or 48 h after doxycycline (Dox; 10 ng/mL) induction. n=6 for each condition and for time points 24 and 48 h. n=3 for each condition and time point 0 h. (**d**) HA-tagged Tent5a WT and Tent5a M1 protein induction upon dox-induction for indicated times. Fold-change, normalized to γ-Tubulin, of 6 h timepoint indicated below. n=2 for each timepoint. (**e**) Western blots showing expression of HA-tagged Tent5a WT and M1 protein in whole cell lysates (n=1 per line), cytoplasmic and nuclear fractions (n=3 each). Parp and Hsp90/γ-Tubulin blots served as controls for nuclear and cytoplasmic fractions, respectively. (**f**) Immunofluorescence images of HA-tag (Alexa 568; *red*) and Hoechst (*blue*) stained INS-1E cells expressing HA-Tent5a WT (*top left*) or HA-Tent5a M1 (*top right*). Negative controls include rabbit IgG control stain (*bottom left*) with dox induction or HA-tag staining in uninduced control cells (*bottom right*). Scale bar: 20 µm. (**g**) Relationship between percentage ploy(A) tail length change versus average poly(A) tail length in uninduced controls. Each point represents a FLAM-seq analyzed transcript. Equation for line of best fit, Spearman correlation coefficient ($R_s$) and accompanied p-value provided. (**h**) $Log_{10}$(average FPKM) distribution of uninduced INS-1E cells used to classify transcripts as low (*teal, blue*) and high (*yellow, red*). This color-code classification was projected onto transcripts ranked by their basal poly(A) tail length with significantly elongated poly(A) tails. Fractions of significantly elongated transcripts in each poly(A) tail length quartiles Q1–Q4 with low to high expression levels presented in pie charts. (**i**) Chart showing the fractions of transcripts identified to have the designation “SignalP-noTM” (*dark purple*) or not (*light purple*) in the genes analyzed by FLAM-Seq (*left*), compared to genes with significantly elongated (*center*) or shortened (*right*) transcripts. (**j–l**) Average poly(A) tail length measured in uninduced control and Tent5a WT-induced cells for (**j**) *Pcolce*, (**k**) *Cstd* and (**l**) *Igfbp2*. n=3. (**m**) Northern blot autoradiograph generated by using a $^{32}$P-labelled *Ins1/2* probe and total RNA harvested from cells transfected with plasmids encoding either human *TENT5C* or *GFP* (Control) for 48 h (n=2 for each condition). Molecular weight marker represents nucleotide length (nct). Lane profiles represent normalized signal intensity (x-axis) from top to bottom of GFP (*grey*), or hTENT5C (*red*) lanes. p-value (based on F-Test) derived from pair-wise comparison testing of means from approximated Gaussian distribution curves. Data are presented as mean ± s.d. in c, j–l. Binomial significance testing performed for proportion of “SignalP-noTM”-containing transcripts expected versus outcome in significantly elongated or shortened poly(A) tail transcripts in i. FLAM-seq analysis with One-way ANOVA used in j-l.

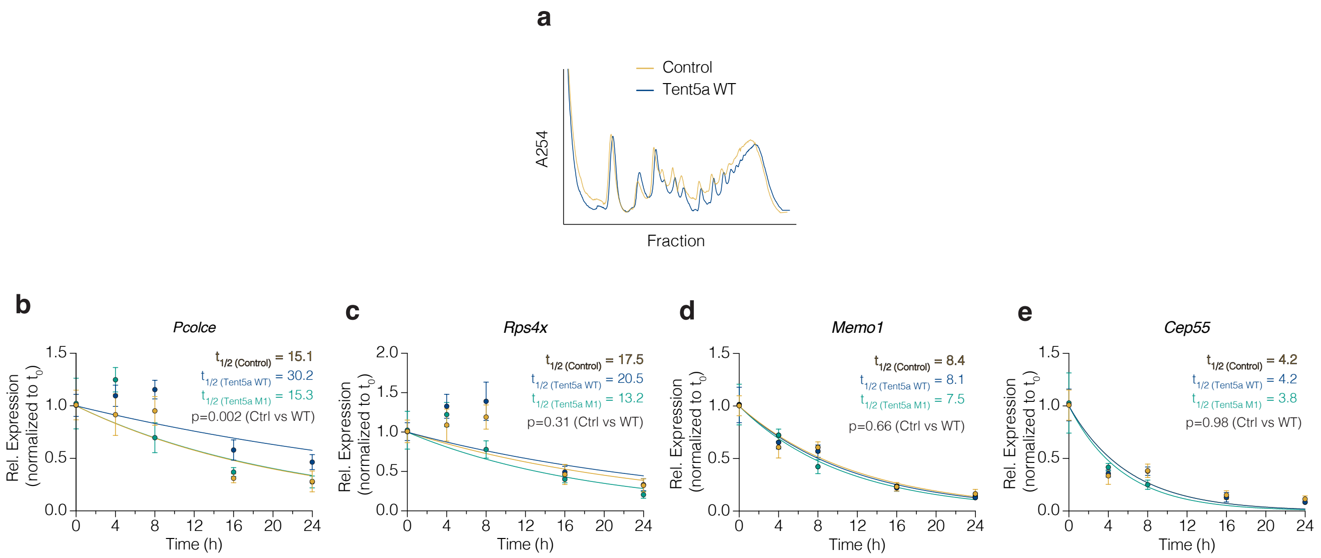


**Figure S4. Tent5a activity elongates poly(A) tails of Ins mRNA.** (**a**) Representative polysome profiles from lysates of control (uninduced, *yellow*), or Tent5a WT overexpressing INS-1E cells (*blue*). (**b-e**) Relative expression of (**b**) *Pcolce*, (**c**) *Rps4x*, (**d**) *Memo1* and (**e**) *Cep55* over the course of treatment with actinomycin D (5 μg/mL) of control (uninduced, *yellow*), Tent5a WT- (*blue*), or Tent5a M1 (*teal*) expressing cells. p-value (based on F-Test) indicates comparison of half-life ($t_{1/2}$) between exponential decay curves generated for control and Tent5a WT data points. n=6 replicates per time point per condition. Data are presented as mean ± s.d. in b–e.

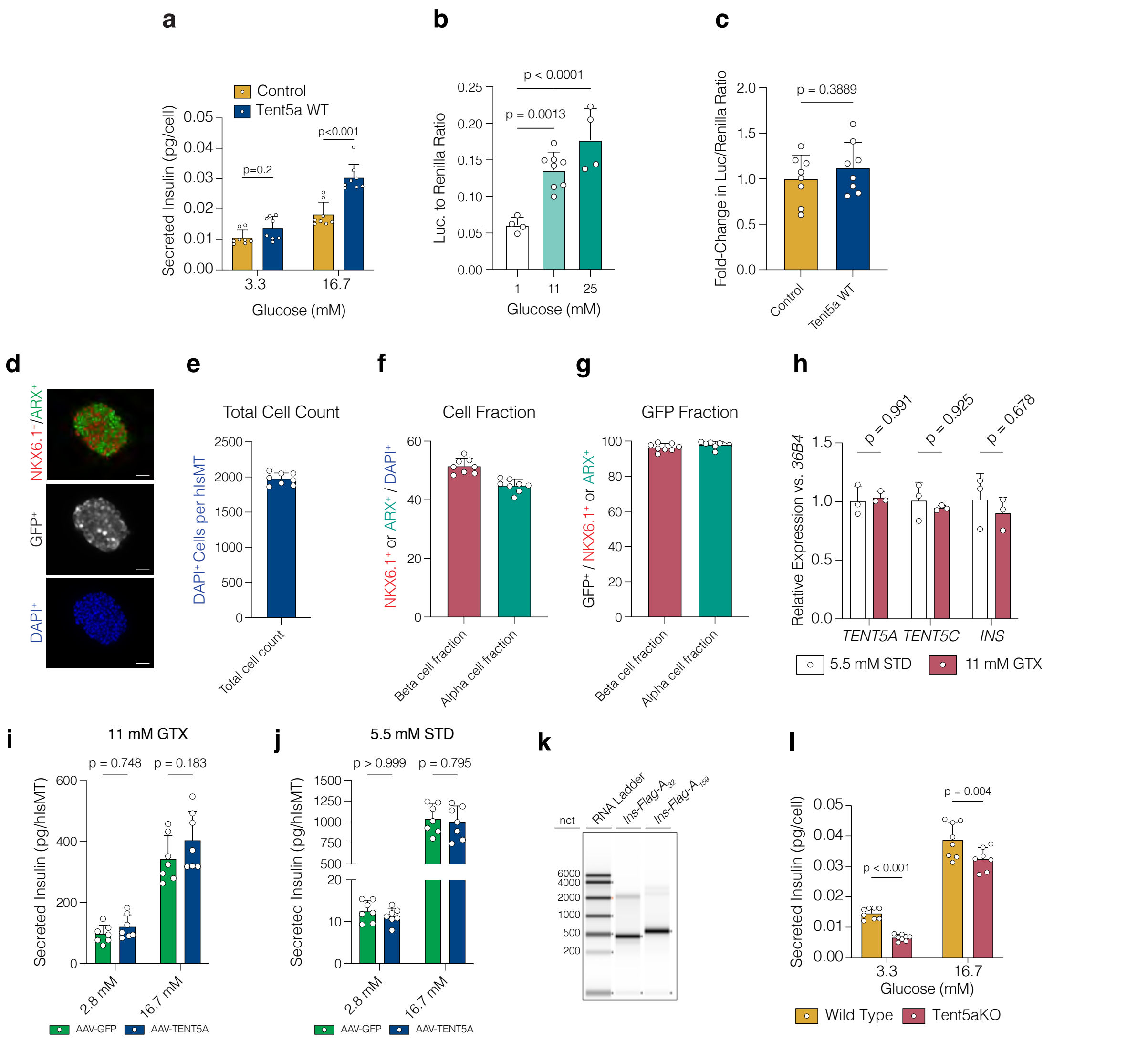


**Figure S5. Tent5a expression regulates cellular insulin content.** (**a**) Quantification of insulin secretion following a 30 min stimulation with 3.3 mM or 16.7 mM glucose and comparing uninduced vs dox-induced Tent5a WT INS-1E cells. n=8 for each group at each glucose condition. (**b**) Luciferase to Renilla Ratio measured from INS-1E cells transfected with plasmids encoding *Renilla* under an SV40 promoter and firefly *Luciferase* under the human *INS* promoter cultured in various glucose concentrations for 24 h. n=4 for 1 and 25 mM glucose, n=8 for 11 mM. (**c**) Fold-change in Luciferase-to-Renilla ratio from lysates of uninduced (Control, *yellow*) and Tent5a WT (*blue*) expressing cells transfected with plasmids encoding *Renilla* and human *INS* promoter-driven firefly *Luciferase*. (**d**) Representative immunofluorescence images showing stainings for NKX6.1 (*red*) and ARX (*green*), GFP (*grey*) and DAPI (*blue*) stains. Scale bar represents 50 µm. (**e, f**) Total cell count estimated from (**e**) DAPI signal, and (**f**) beta cell (*red*) and alpha cell (*green*) fractions identified by NKX6.1 or ARX signals, respectively. (**g**) Percentage GFP-positive beta and alpha cell populations. n=8 hIsMTs per quantification. (**h**) Relative expression of *TENT5A*, *5C* and *INS* in human islet micro-tissues cultured for 7 days in 5.5 mM glucose STD (*white*) or 11 mM glucose GTX (*red*) conditions. n=3 pools of 12 hIsMTs per gene per condition. (**i,j**) GSIS under (**i**) GTX or (**j**) STD culture conditions of GFP- (*green*) or TENT5A- (*blue*) overexpression. n=7 hIsMTs for each group. (**k**) Tape Station gel electrophoresis of in vitro transcribed *Ins-Flag-A*$_{32}$ or *Ins-Flag-A*$_{159}$ transcripts. (**l**) Quantification of insulin secretion following a 30 min stimulation with 3.3 mM or 16.7 mM glucose and comparing wildtype INS-1E and Tent5aKO cells. n=8 for each group at each glucose condition except Tent5aKO, 16.7 mM (n=7). Data represented as mean ± s.d. in a–c, e–j, and l. Two-Way ANOVA with Šídák multiple comparison testing used to analyze a, h, i, j and l. One-Way ANOVA followed by Dunnett's multiple comparison testing used to analyze b. Two-sided unpaired t-test used to analyze data in c.

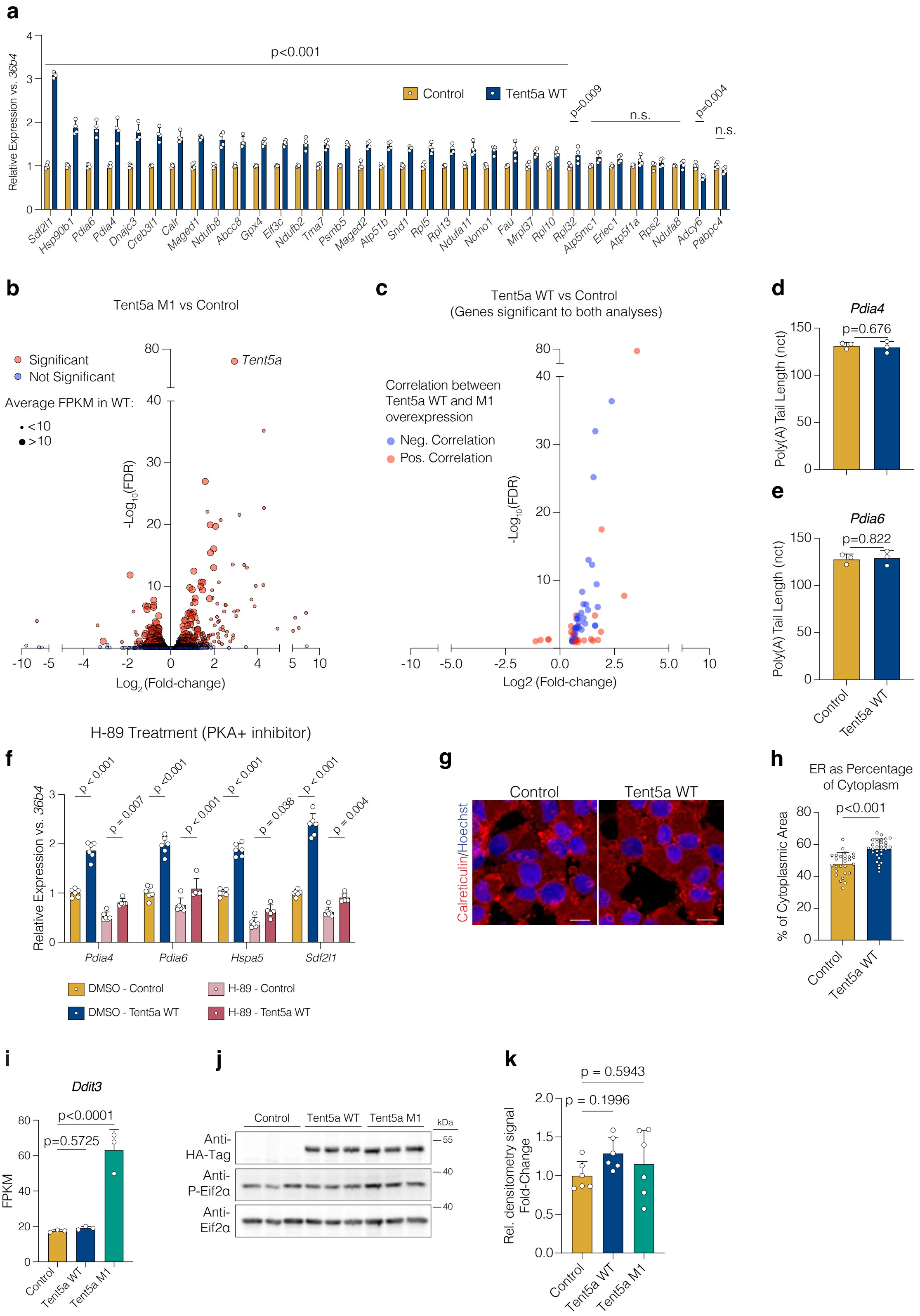


**Figure S6. Tent5a activity promotes increased ER function.** (**a**) qPCR validation of most regulated RNAseq-identified transcripts in uninduced and Tent5a WT-expressing INS-1E cells. n=4 replicates per condition for each gene. (**b**) Differential expression analysis (DEA) of INS-1E cells induced to express Tent5a M1 compared to uninduced control cells. Significant (*red*) or non-significant (*blue*) genes were defined by an FDR cut-off of 0.01 and $Log_2$(fold-change) ± 0.5. Genes with an average FPKM greater than 10 in control cells are represented as larger data points. (**c**) DEA of Tent5a WT vs uninduced control cells showing only significantly regulated genes common to both DEAs of Tent5a WT and Tent5a M1. Genes are color-coded to identify those with a positive (*red*) or negative (*blue*) correlation between the two analyses. (**d,e**) Average poly(A) tail length measured by FLAM-seq in uninduced and Tent5a WT expressing cells for *Pdia4* (**d**) and *Pdia6* (**e**). n=3. (**f**) Expression of ER chaperone protein transcripts in cells that were treated with DMSO or 20 μM H-89 in the presence or absence of 10 ng/mL doxycycline for 24 h. n=6. (**g**) Immunofluorescence staining of the ER with Calreticulin (*red*) and Hoechst (*blue*) in uninduced and Tent5a WT-expressing cells. Scale bar: 20 μm. (**h**) Cell-based quantification of calreticulin-stained ER as a percentage of the cytoplasmic area in uninduced control (n=28) and Tent5a-WT expressing cells (n=29). (**i**) *Ddit3* expression levels from RNA-seq of control (yellow) compared Tent5a WT (blue) or Tent5a M1 (teal) overexpressing cells. n=3. P-values derived from DEA. (**j**) Western blot and (**k**) densitometry of whole cell lysates of uninduced control, Tent5a WT or Tent5a M1 overexpressing samples probed for HA-tag, phosphor- and total Eif2α. n=3 and n=6 for each condition. Data are presented as mean ± s.d. in a, d–f, h–I and k. FLAM-seq analysis with One-way ANOVA used in d and e. Two-way ANOVA followed by Šídák multiple comparison testing used to analyze f. Two-suded unpaired t-test used to analyze data in h. One-way ANOVA followed by Dunnett's multiple comparison testing used to analyze k.

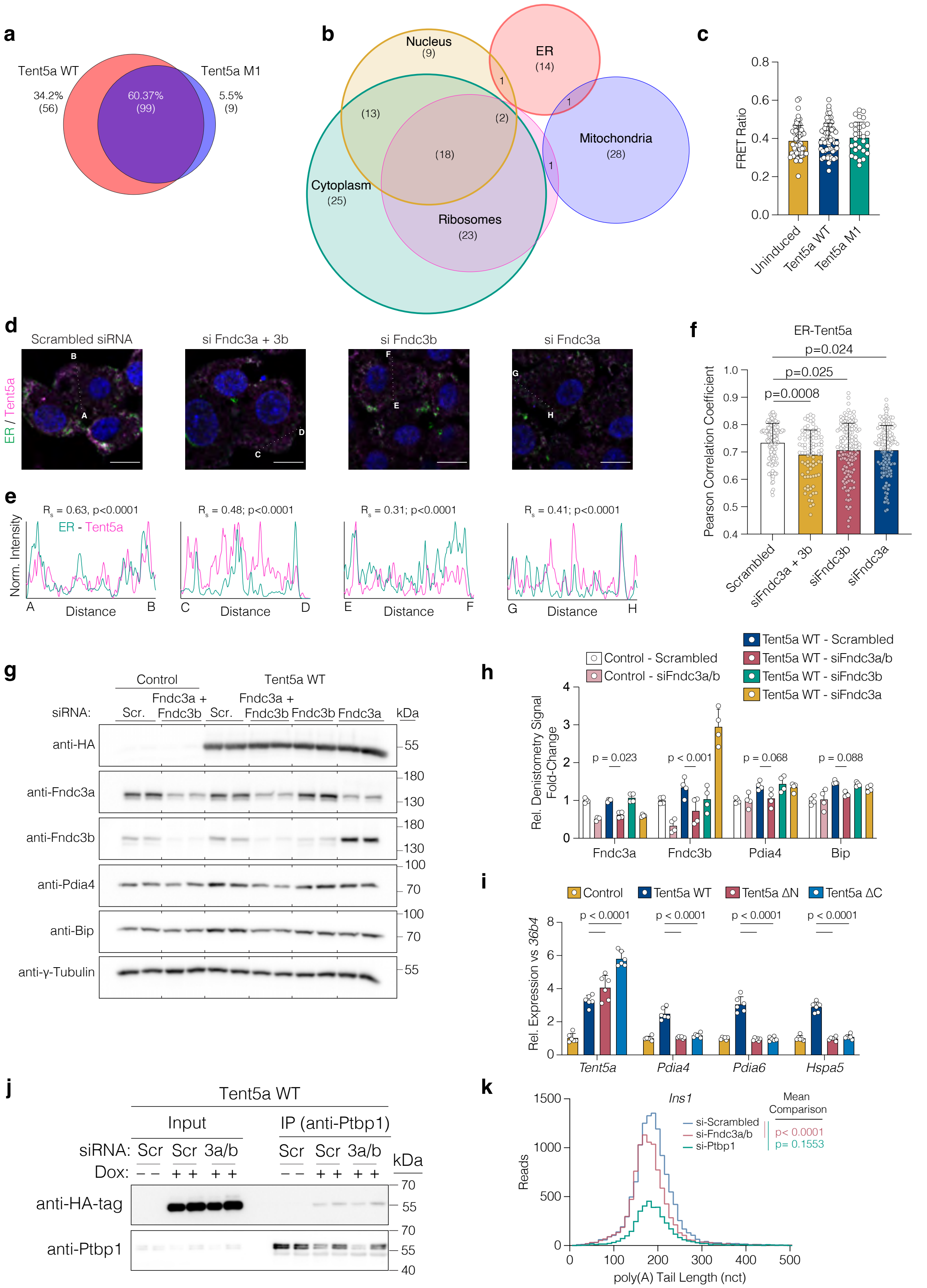


**Figure S7. Fndc3a/b-Tent5a association at the ER promotes polyadenylation.** (**a**) Percentage overlap in proteins identified in the interactomes of Tent5a WT (*red*) and Tent5a M1 (*blue*). (**b**) Subcellular localization of Tent5a WT interactome proteins. (**c**) FRET ratio measurements of the FRET ER-Mitochondrial Proximity (FEMP) sensor transfected in uninduced control Tent5a WT- or Tent5a M1-expressing cells. (**d**) Immunofluorescence images exhibiting the HA-tag of TENT5A WT (*magenta*), the ER via GFP-staining of the ER-tagged EroGFP construct (*green*) and Hoechst-stained nuclei (*blue*) in cells transfected with scrambled siRNA control, or siRNA against Fndc3a, Fndc3b, or both. Scale bar: 10 µm. (**e**) Plots of lines from points A to B, C to D, E to F and G to H for ER (*green*) and Tent5a-WT (*magenta*) color-coded to match image pseudo-coloring. (**f**) Pearson correlation coefficient between Tent5a and ER in cells treated with scrambled siRNA (n=134), or siRNAs targeting both Fndc3a and 3b (n=88), Fndc3b (n=149) or Fndc3a (n=152) only. (**g**) Western blots and (**h**) densitometry of indicated proteins from lysates of either uninduced or Tent5a WT overexpressing cells exposed to either scrambled siRNA controls or siRNAs targeting Fndc3a, Fndc3b, or both. Fold-change regulation of proteins normalized to γ-Tubulin of uninduced and scrambled siRNA controls. n=4 for each condition. (**i**) Expression of Tent5a and ER chaperone proteins in uninduced (*yellow*) or Tent5a WT (*dark blue*), Tent5a-ΔN (*red*) or Tent5a-ΔC (*light blue*). n=6 replicates for each condition measured for each gene. (**j**) Western blots for the HA-tag and Ptbp1 following immunoprecipitation of endogenous Ptbp1 in uninduced control cells Tent5a WT-expressing cells transfected with either scrambled or Fndc3a- and 3b- targeting siRNA. (**k**) Histogram of *Ins1* transcript poly(A) tail lengths measured during Tent5a WT overexpression with 48 h exposure to scrambled siRNA (*blue*), siRNA targeting Fndc3a/b (*red*) or Ptbp1 (*teal*). p-values (based on F-Test) compare the means of Gaussian fits of each data set against that of the si-scrambled control. Data are presented as mean ± s.d. in c, f, h and i. One-way ANOVA followed by Dunnett's multiple comparison testing used to analyze f. Two-Way ANOVA followed by Šídák multiple comparison testing used to analyze h and i.
