# Supplementary material for: Polyadenylation of insulin mRNA by Tent5a regulates pancreatic beta cells
Source: Nat Commun. 2026 May 20;17:7295. doi: 10.1038/s41467-026-72905-8 (PMC13402609; doi:10.1038/s41467-026-72905-8)
Supplement: Supplementary file 2 — Description of Additional Supplementary Files [file 41467_2026_72905_MOESM2_ESM.pdf]

Description of Additional supplementary files

Supplementary Data 1: Oligonucleotide sequences (5’- 3’) in siRNA pool that was used in the RBP RNAi screen.

Supplementary Data 2: Results and statistical significance of the glucose-stimulated insulin secretion in response to gene silencing of indicated transcripts (Gene). Two-sided unpaired t-test was applied for comparisons of siRNA treated vs control samples. Significant response identified as a fold-change of 1 and SSMD score of 1.

Supplementary Data 3: Insulin content measurements in transfected INS1 cells and statistical significance in response to gene silencing of indicated transcripts (Gene). Two-sided unpaired t-test was applied for comparisons of siRNA treated vs control samples.

Supplementary Data 4: Viability readout (%) of transfected INS1 cells in response to gene silencing of indicated transcripts (Gene).

Supplementary Data 5: Glibenclamide-stimulated insulin secretion and statistical significances of transfected INS1 cells in response to gene silencing of indicated transcripts (Gene). Two-sided unpaired t-test was applied for comparisons of siRNA treated vs control samples.

Supplementary Data 6: Glucose-stimulated ATP generation in high (16.7 mM) glucose upon RBP silencing of indicated transcripts (Gene). Two-sided unpaired t-test was applied for comparisons of siRNA treated vs control samples.

Supplementary Data 7: Protein identification of Tent5a immunoprecipitation experiment in INS1E cells using label free MS analysis.

Supplementary Data 8: The sequences of all primers used in this study.

Supplementary Data 9: List of all antibodies and respective dilutions for immunohistochemistry and Western blotting.
