# Supplementary material for: Polyadenylation of insulin mRNA by Tent5a regulates pancreatic beta cells
Source: Nat Commun. 2026 May 20;17:7295. doi: 10.1038/s41467-026-72905-8 (PMC13402609; doi:10.1038/s41467-026-72905-8)
Supplement: Supplementary file 7 — Reporting Summary [file 41467_2026_72905_MOESM7_ESM.pdf]

nature portfolio

Corresponding author(s): Markus Stoffel

Last updated by author(s): Mar 27, 2026

Reporting Summary

Nature Portfolio wishes to improve the reproducibility of the work that we publish. This form provides structure for consistency and transparency in reporting. For further information on Nature Portfolio policies, see our Editorial Policies and the Editorial Policy Checklist.

Statistics

For all statistical analyses, confirm that the following items are present in the figure legend, table legend, main text, or Methods section.

n/a	Confirmed	
☐	☒	The exact sample size (*n*) for each experimental group/condition, given as a discrete number and unit of measurement
☐	☒	A statement on whether measurements were taken from distinct samples or whether the same sample was measured repeatedly
☐	☒	The statistical test(s) used AND whether they are one- or two-sided<br>*Only common tests should be described solely by name; describe more complex techniques in the Methods section.*
☐	☒	A description of all covariates tested
☐	☒	A description of any assumptions or corrections, such as tests of normality and adjustment for multiple comparisons
☐	☒	A full description of the statistical parameters including central tendency (e.g. means) or other basic estimates (e.g. regression coefficient) AND variation (e.g. standard deviation) or associated estimates of uncertainty (e.g. confidence intervals)
☐	☒	For null hypothesis testing, the test statistic (e.g. *F*, *t*, *r*) with confidence intervals, effect sizes, degrees of freedom and *P* value noted<br>*Give P values as exact values whenever suitable.*
☒	☐	For Bayesian analysis, information on the choice of priors and Markov chain Monte Carlo settings
☒	☐	For hierarchical and complex designs, identification of the appropriate level for tests and full reporting of outcomes
☒	☐	Estimates of effect sizes (e.g. Cohen's *d*, Pearson's *r*), indicating how they were calculated

Our web collection on statistics for biologists contains articles on many of the points above.

Software and code

Policy information about availability of computer code

Data collection	No specific software was used
Data analysis	For short read RNA sequencing analysis, raw reads were cleaned and aligned to the rat genome on the SUSHI platform using the KallistoApp.<br>Differentially expressed genes were identified using the R package DESeq2.<br>Image quantification was performed using Fiji version 2.1.0.<br>Western Blot densitometry was performed using Fiji version 2.1.0.<br>Statistical analysis was performed using GraphPad Prism version 10.

For manuscripts utilizing custom algorithms or software that are central to the research but not yet described in published literature, software must be made available to editors and reviewers. We strongly encourage code deposition in a community repository (e.g. GitHub). See the Nature Portfolio guidelines for submitting code & software for further information.

Data

Policy information about availability of data

All manuscripts must include a data availability statement. This statement should provide the following information, where applicable:

- Accession codes, unique identifiers, or web links for publicly available datasets
- A description of any restrictions on data availability
- For clinical datasets or third party data, please ensure that the statement adheres to our policy

Long reads (Flam Seq) and Raw reads of RNA seq were deposited at NCBI Sequence Read Archive (SRA, www.ncbi.nlm.nih.gov/sra) and are accessible through the accessions PRJNA1267696 and PRJNA1267696, respectively.
The raw mass spectrometry data has been deposited in the ProteomeXchange under accession number PXD066929.

Field-specific reporting

Please select the one below that is the best fit for your research. If you are not sure, read the appropriate sections before making your selection.

☒ Life sciences ☐ Behavioural & social sciences ☐ Ecological, evolutionary & environmental sciences

For a reference copy of the document with all sections, see nature.com/documents/nr-reporting-summary-flat.pdf

Life sciences study design

All studies must disclose on these points even when the disclosure is negative.

Sample size	No statistical methods were used to calculate sample size. Sample sizes were determined based on previously published data using similar methods.
Data exclusions	No data were excluded from analyses.
Replication	In all animal experiments, one sample (n) represents one animal. In cell culture experiments, one sample (n) represents one well of a multi-well plate. In Western blots, samples were assayed in unicates and quantifications are representative of at least 2 experiments. In qPCR and hormone measurements, samples were assayed in duplicates and reported as the average per sample. All attempts at replication were successful.
Randomization	For most animal experiments, the breeding strategy was such that all mice in a litter were either controls or transgenic/knockouts; therefore, all mice in a litter were used for experiments. Multiple litters were combined for each cohort to achieve sufficient sample sizes.
Blinding	Investigators were blinded to group allocation during data collection, sample collection/processing, and analysis.

Reporting for specific materials, systems and methods

We require information from authors about some types of materials, experimental systems and methods used in many studies. Here, indicate whether each material, system or method listed is relevant to your study. If you are not sure if a list item applies to your research, read the appropriate section before selecting a response.

Materials & experimental systems

n/a	Involved in the study	
☐	☒	Antibodies
☐	☒	Eukaryotic cell lines
☒	☐	Palaeontology and archaeology
☐	☒	Animals and other organisms
☐	☒	Human research participants
☒	☐	Clinical data
☒	☐	Dual use research of concern

Methods

n/a	Involved in the study	
☒	☐	ChIP-seq
☒	☐	Flow cytometry
☒	☐	MRI-based neuroimaging

Antibodies

Antibodies used	anti-insulin (3014S, Cell Signaling), anti-HA tag (3724S, Cell Signaling), anti-GAPDH (14C10) (2118S, Cell Signaling), anti-γ-Tubulin (T6557, Sigma), anti-P-p38 Mapk (Thr180/Tyr182) (4511S, Cell Signaling), anti-p38 Mapk (8690S, Cell Signaling), anti-P-Eif2α (Ser51) (3398S, Cell Signaling), anti-Eif2α (5324S, Cell Signaling), anti-Parp (9542S, Cell Signaling), anti-Hsp90 (4874S, Cell Signaling), anti-Calreticulin (2891S, Cell Signaling), anti-Ddost (ProteinTech, 14916-1-AP), anti-Nucb2 (abcam ab224348), anti-Gnas (ProteinTech, 10150-2-AP), anti-Pdia4 (ProteinTech, 14712-1-AP), anti-Pdia6 (ProteinTech, 18233-1-AP), anti-GFP (Life Technologies, A21311), anti-Tent5a (Davids Biotechnologie, FYE), anti-Fndc3b (Novus Biological, NBP1-90495), anti-Fndc3a (Novus Biological, NBP1-88809), anti-Ptbp1 (Sigma, WH0005725M1), anti-NKX6.1 (abcam, ab221549), anti-ARX (R&D Systems, AF7068-SP) , goat anti-rabbit conjugated to Cy5 (A10523, Invitrogen), goat anti-mouse conjugated to Alexa 647 (115-605-003, Jackson ImmunoResearch), goat anti-mouse conjugated to HRP (401253 Sigma), and goat anti-rabbit conjugated to HRP (401393, Sigma).
Validation	Antibody validation is available through manufacturer's websites.<br><br>Rabbit anti-insulin (3014S, Cell Signaling): https://www.cellsignal.com/products/primary-antibodies/insulin-c27c9-rabbit-monoclonal-antibody/3014?srsltid=AfmBOopOTSHBdjr9ito9teRLOmIlxBVYgDguaJPrkWOMKTI5n3owk0kA<br>Rabbit anti-HA tag (3724S, Cell Signaling): https://www.cellsignal.com/products/3724/datasheet?images=1&protocol=0&srsltid=AfmBOoo_elqvRwv9S9MD_0cZ-YDb7rDGUCnOjilB2jsHqbHZZNIQ9LO6

Rabbit anti-GAPDH (14C10) (2118S, Cell Signaling): https://www.cellsignal.com/products/primary-antibodies/gapdh-14c10-rabbit-monoclonal-antibody/2118?srsltid=AfmBOooZgc1Fr6CcZ6z6qVZiZIcCuBZdH_bxEo9kup0S4a4g7nDbvfqa
Mouse anti-γ-Tubulin (T6557, Sigma): https://www.sigmaaldrich.com/CH/fr/product/sigma/t6557?utm_source=google&utm_medium=cpc&utm_campaign=18067744937&utm_content=168605485099&gad_source=1&gad_campaignid=18067744937&gbraid=0AAAAAD8kLQRA99voWjbvb3qvnaX5Ry5HB&gclid=EAIaIQobChMI9pO8s626kgMVbpODBx1rJyZuEAAYAyAAEgKE9PD_BwE
Rabbit anti-P-p38 Mapk (Thr180/Tyr182) (4511S, Cell Signaling): https://www.cellsignal.com/products/primary-antibodies/phospho-p38-mapk-thr180-tyr182-d3f9-rabbit-monoclonal-antibody/4511?srsltid=AfmBOopvmzpUttQ8U4sL567RAxFblvn315AUErulKOs1OAS-OSK3j4Dc
Rabbit anti-p38 Mapk (8690S, Cell Signaling): https://www.cellsignal.com/products/primary-antibodies/p38-mapk-d13e1-rabbit-monoclonal-antibody/8690?srsltid=AfmBOopcFD_TE8g49n1_3RK__U_N1iG2u2Ig7VITWxITo7J37JowS_9y
Rabbit anti-P-Eif2α (Ser51)(3398S, Cell Signaling): https://www.cellsignal.com/products/primary-antibodies/phospho-eif2-alpha-ser51-d9g8-rabbit-monoclonal-antibody/3398?srsltid=AfmBOorURiHEgIqdp0miS88CEeatN-iV4s_I1GOsRhTS7XzrzXngRNH5
Rabbit anti-Eif2α (5324S, Cell Signaling): https://www.cellsignal.com/products/primary-antibodies/eif2-alpha-d7d3-rabbit-monoclonal-antibody/5324?srsltid=AfmBOooXH166PcEkVsA9swW3Zau9a2zniGDkzn2Tf-8B9UsIurTBHYiG
Rabbit anti-Parp (9542S, Cell Signaling): https://www.cellsignal.com/products/primary-antibodies/parp-antibody/9542?srsltid=AfmBOopvL66oK5GBBJ91lfef3DZlsxqOVFbIYQsm2n7yG77I4QMlH0Zq
Rabbit anti-Hsp90 (4874S, Cell Signaling): https://www.cellsignal.com/products/primary-antibodies/hsp90-antibody/4874?srsltid=AfmBOop31_W0YtQq2-rAcBc3dRQdn6l9Bl1lXMWpQQ3KhRtKDKsGSvf3
Rabbit anti-Calreticulin (2891S, Cell Signaling): https://www.cellsignal.com/products/primary-antibodies/calreticulin-antibody/2891?srsltid=AfmBOoocZpWZ-OUJJeY0Cnig3HXGuO6hSg_zA16KDDUcUpoIkcgZ1ykt
Rabbit anti-Ddost (ProteinTech, 14916-1-AP): https://www.ptglab.com/Products/Pictures/pdf/14916-1-AP.pdf?srsltid=AfmBOoro0Uxd8DlF8PB1Uu1rmw-_MDMeGOU47GXoFhe4-KU_lhu38C2e
Rabbit anti-Nucb2 (abcam ab224348):
https://www.abcam.com/en-us/products/primary-antibodies/nucb2-antibody-ab224348
Rabbit anti-Gnas (ProteinTech, 10150-2-AP): https://www.ptglab.com/products/NESP55,GNAS-Antibody-10150-2-AP.htm?srsltid=AfmBOoq7b0ohUn0uZk978R_-wNn91_K9V3cKkBDNxKnaXRmma8EF0ZfF
Rabbit anti-Pdia4 (ProteinTech, 14712-1-AP):
https://www.ptglab.co.jp/products/PDIA4-Antibody-14712-1-AP.htm
Rabbit anti-Pdia6 (ProteinTech, 18233-1-AP): https://www.ptglab.com/de/products/PDIA6-Antibody-18233-1-AP.htm?srsltid=AfmBOopMnjgRtvx8Vo2tw9xTpw2ptoWmO0ARhNmcNp0erPgH9lzB7Xr2
Rabbit anti-GFP (Life Technologies, A21311): https://www.fishersci.de/shop/products/gfp-polyclonal-antibody-alexa-fluor-488/10797644?srsltid=AfmBOorjFnPldto7s9jWVXJ637fWk2QZaJ_RMcUhhV3yjuP6gYuFthHK
Rabbit anti-Fndc3b (Novus Biological, NBP1-90495): https://www.novusbio.com/products/fndc3b-antibody_nbp1-90495?srsltid=AfmBOopbEMp86l8Jy_jO9VIkNjCTcvxC7hc3j_bWAIsd3Km2Lz915se9
Rabbit anti-Fndc3a (Novus Biological, NBP1-88809): https://www.novusbio.com/products/fndc3a-antibody_nbp1-88809?srsltid=AfmBOoqszpxhaBgmQ1HEWCk26gTUvNF0FQim8oqYHiH90WIF6p7gd561
Mouse anti-Ptbp1 (Sigma, WH0005725M1): https://www.sigmaaldrich.com/CH/fr/product/sigma/wh0005725m1?srsltid=AfmBOopZCpuBISjozI_u1bCezAKsTVHrXCXjukywiVsHYYVPbh997p2T
Rabbit anti-NKX6.1 (abcam, ab221549):
https://www.abcam.com/en-us/products/primary-antibodies/nkx61-antibody-epr20405-ab221549
Sheep anti-ARX (R&D Systems, AF7068-SP): https://www.rndsystems.com/products/human-arx-antibody_af7068
Goat anti-rabbit conjugated to Cy5 (A10523, Invitrogen): https://www.thermofisher.com/antibody/product/Goat-anti-Rabbit-IgG-H-L-Cross-Adsorbed-Secondary-Antibody-Polyclonal/A10523
Goat anti-mouse conjugated to Alexa 647 (115-605-003, Jackson ImmunoResearch): https://www.thermofisher.com/antibody/secondary/query/goat+anti-mouse+Alexa+647/filter/species/Mouse?ef_id=EAIaIQobChMI0IHMmLC6kgMVl7KDBx2AzivUEAAYASAAEgKnxPD_BwE:G:s&s_kwcid=AL!3652!3!605801922710!p!!g!!goat%20anti%20mouse%20647!596889499!104138578459&cid=bid_pca_aus_r01_co_cp1359_pjt0000_bid00000_0se_gaw_nt_pur_con&gad_source=1&gad_campaignid=596889499&gbraid=0AAAAADxi_GQUvkslHrQ14y06_xeCVHjlP&gclid=EAIaIQobChMI0IHMmLC6kgMVl7KDBx2AzivUEAAYASAAEgKnxPD_BwE
Goat anti-mouse conjugated to HRP (401253 Sigma): https://www.sigmaaldrich.com/CH/fr/product/mm/401253?srsltid=AfmBOoqUSukGUbeMj_rRPrwwXCUi-mqECHH2HTy34yfdCJVUyBl1l-hC
Goat anti-rabbit conjugated to HRP (401393, Sigma): https://www.sigmaaldrich.com/CH/fr/product/sigma/a3687?utm_source=google&utm_medium=cpc&utm_campaign=21480226247&utm_content=164812878133&gad_source=1&gad_campaignid=21480226247&gbraid=0AAAAAD8kLQRm3nPxiK1ahZiRIMPYLj7ZO&gclid=EAIaIQobChMIs9DytrC6kgMVXJCDBx0hpCbyEAAYAiAAEgKARvD_BwE

Eukaryotic cell lines

Policy information about cell lines

Cell line source(s)	INS-1E cells. Gift from Boehringer Ingelheim.
Authentication	Cell lines were used as received without authentication.
Mycoplasma contamination	Cell lines were not tested for mycoplasma contamination.
Commonly misidentified lines (See ICLAC register)	No commonly misidentified cell lines were used in the study.

Animals and other organisms

Policy information about studies involving animals; ARRIVE guidelines recommended for reporting animal research

Laboratory animals	All mouse lines were established on a C57BL/6 genetic background. Lep ob/ob mice (B6 ob/ob Mice), Alita (C57BL/6-Ins2Akita/J), and wildtype mice (C57BL/6NJ) were purchased from The Jackson Laboratory. All mice were maintained on a C57BL/6 background. Both males and females between 4-35 weeks of age were used for experiments. Mice were housed in a pathogen-free animal facility at the Institute of Molecular Health Sciences at ETH Zürich, in a temperature-controlled room (22°C) with 55% humidity and with a 12-hour light/12-hour dark cycle (lights on from 06:00 to 18:00). Mice were fed a standard laboratory chow diet (Kliba Nafag 3437; containing 3 kcal/g metabolizable energy with 4.5% fat, 18.5% protein, and 38% carbohydrate [w/w]) or high-fat diet (HFD) (SAFE diets 260HF; containing 5.5 kcal/g metabolizable energy with 36% fat, 20% protein, 37% carbohydrate, and 18% sucrose [w/w]). Feeding with HFD began at 4-5 weeks of age.
Wild animals	No wild animals were used in the study.
Field-collected samples	No field collected samples were used in the study
Ethics oversight	All animal experiments were approved by the Kantonale Veterinäramt Zürich.

Note that full information on the approval of the study protocol must also be provided in the manuscript.

Human research participants

Policy information about studies involving human research participants

Population characteristics	n.a.
Recruitment	n.a.
Ethics oversight	Formal consent from human islet donors was not required because samples were discarded islets from de-identified humans.

Note that full information on the approval of the study protocol must also be provided in the manuscript.
