# Supplementary material for: Polyadenylation of insulin mRNA by Tent5a regulates pancreatic beta cells
Source: Nat Commun. 2026 May 20;17:7295. doi: 10.1038/s41467-026-72905-8 (PMC13402609; doi:10.1038/s41467-026-72905-8)
Supplement: Supplementary file 8 — Transparent Peer Review File [file 41467_2026_72905_MOESM8_ESM.pdf]

nature portfolio

Peer Review File

Polyadenylation of Insulin mRNA by Tent5a regulates pancreatic beta cells

Corresponding Author: Professor Markus Stoffel

This file contains all reviewer reports in order by version, followed by all author rebuttals in order by version.

Version 0:

Reviewer comments:

Reviewer #1

(Remarks to the Author)
The study by Silva et al. identifies a role of TENT5A non-canonical polyA polymerase in regulation of insulin production in mouse pancreatic cell line model. The authors perform a screen of RNA binding proteins looking for factors relevant to insulin production. This alone is a remarkable work, and should be a usefull resource for other researchers. Next, using two pancreatic disease models they identify Tent5A as the RNA binding protein with most altered abundance. Since Tent proteins have signatures of non-cannonical polyA polymerases the authors evaluate both pA-tail lengths and mRNA abundance to find that alterations in Tent5A levels are connected to changes in abundance and adenylation of mRNAs involved in insulin biogenesis, which they confirm on a protein level. Finally, authors show taht TENT5A interacts with FNDC3B; a protein associated with the ER. They show that abolishing Tent5A association with FNDC3B results in alleviation of increased insulin production observed upon Tent5A overexpression.

Altogether, the study seems a comprehensie and engaging work, and I would like to see it published in Nature Communications. However, my overall impression is that authors have too hastingly prepared the submission, so that some aspects of the publication cannot be appreciated, whereas others are outright defective (in terms of presentation), potentially leading to readers mistrust in results. This is based on following observations:

- No PRIDE submission of raw data is listed in the manuscript.

- No submission of RNA-seg and FLAM-seq raw data is prepared. Material and methods state simply 'accession number pending'.

- Figures S3 and S4 are missing from the supplementary figure file! Instead Figs 3 and 4 are repeated. This precludes the evaluation of large segments of the main text.

****

Major general, and conceptual remarks:

General formal remark. Starting from fig. 3 the authors are a bit dismissive about the cell line type in which they perform their experimesnts. the M&M section states several mouse and human cell lines, so it is important to inform the reader what the model organism is. In this context it would be helpful to improve the nomenclature of genes, as most readers are accustumed to Tent5A designating a mouse protein and TENT5A a human one. For example such inconsistencies are in Fig.2a and 2b and other places.

To validate the proposed recruitment of TENT5A via PTBP1, did the authors try to show that in conditions of PTBP1 silencing target mRNAs do not associate with TENT5A; eg. using RIP-qPCR or simply looking at pA-tail lengths the same manner as they did for FNDC3A (Fig. 5h)? Alternatively, was the pirimidine rich segment of INS1 RNA ever deleted and shown to impact pA-tails (even as transfected constructs)? Perhaps it's worth to consider performing those experiemnts? Do other TENT5A targets also possess such motifs?

A model scheme would be perfect to fully understand the proposed working model.

****

Major remarks specific to data:

Figure 2a and 2c -> the opposite change in Tent5a expression 'B6-0b/ob vs B6-ob/+' compared to 'Akita vs WT' is one of the criteria of selecting the protein for further studies. The reader would like to appreciate if more such proteins were selected in the screen. Perhaps to this end a scatterplot comparing the significant RPBs fold change (eg. on x-axis 'B6-0b/ob vs B6-ob/+' compared to 'Akita vs WT' on y-axis) would be helpful. Was the decrease in Tent5A protein levels also confirmed by western blot? Shouldn't it be shown together with the western on panel 2b?

Figure 2j -> quote lines 190-191 'Furthermore, anisomycin increased phosphorylation of p38 MAPK at Thr180/Tyr182, which is consistent with activation of p38 MAPK during ribosome collision (Fig. 2j) 33' -> looking at the western blot I cannot see any change in phospho-p38 levels. Western is not very quantitative, so the densitrometry data holds the conclusion together. However, to convince the reader I would ask for 6 more replicates and some statistics. Same applies to Fig 4m and 4p. Importantly, the densitrometry protocol is not provided in material and methods. Please include it in the revised version.

Fig. 3j -> the error bars seem off. Some are not centered around data points.

Fig. 4k and related text -> text quote 'in addition, Tent5a WT induced an increase in cellular ATP levels consistent with enhanced mitochondrial ATP production (Fig. 4k) 52-54'. Quote is inconsitent with the content of the figure panel (legend quote: '(k) Western blots depicting the protein levels of DDOST, NUCB2 and GNAS in uninduced compared Tent5a WT or Tent5a M1 overexpressing cells. n=3.'). Nontheless, how is ATP level relevant to a simple enzyme-substrate (TENT5A-mRNA) recruitment mechanism? ATP is abundant , and an increase in ATP levels might just be a means to support incresed protein production and secretion.

Fig. 5a - Unless I missed it somewhere in the files, it would be helpfull if the authors provided an excell file summing up the hits obtained in mass spectrometry analysis together with a list of significantly enriched proteins.

Fig. 5j -> IP using PTBP1 as bait, please show the bait protein. It is impossible to evaluate the result unless the reader sees equal amounts of bait protein.

Figure S5 - IF data. If the dependency of TENT5A localization to ER on Fndc3a was assessed only by inspecting single images, and using the lines drawn arbitrarily then the conclusions put forward seem not convicing. Please use an appropriate protocol to quantify several replicates and provide statistical evaluation of the data. In any case, even if colocalization is not fully abolished, does this really imact the mechanistic model?

Figure S5g - essentially same remark as Fig.5j, additionally the legend quote (line 1254-1259) does not reflect the content of the figure: '(g) Western blot for the HA-tag following immunoprecipitation of PTBP1 in uninduced control cells or those induced with doxycycline to express Tent5a WT transfected with either scrambled or Fndc3a and 3b targeting siRNA. Data are presented as mean ± s.d. in a, d and f. One-way ANOVA followed by Dunnett's Multiple comparison testing used to analyze d. Two-Way ANOVA followed by Šídák multiple comparison testing used to analyze f.' -> The panel shows a HA-tag western blot. It is not clear what the HA-tagged protein is; was it TENT5A?. Furthermore the bait PTBP1 protein is not shown. Please clarify this panel and its legend.

Supplementary Table S11 seems damaged?

****

Minor:

lines 366-367 repetition: 'Our systematic screens suggest that RBP mainly are mainly positive regulators (...)'

Naming the result section as 'main' seems unorthodox?

Fig. 5j -> anit-PTBP1, should be 'anti-'

Reviewer #2

(Remarks to the Author)
This manuscript presents an intriguing investigation into the mechanisms underlying beta cell compensation in response to metabolic stress. Through a comprehensive RNAi screen of RNA-binding proteins, the authors identify Tent5a as a critical regulator of insulin production and secretion in pancreatic beta cells. The work demonstrates that Tent5a expression is upregulated during beta cell compensation in obesity models and functions by elongating poly(A) tails of insulin mRNAs,

thereby enhancing their stability. The authors further elucidate a mechanistic model whereby Tent5a is tethered to the endoplasmic reticulum via FNDC3 proteins, with PTBP1 facilitating the interaction with insulin mRNA.

The study tackles an important molecular mechanism that improves our understanding of post-transcriptional regulation in beta cell function and potential pathways for therapeutic intervention in diabetes. The multi-faceted experimental approach combining RNAi screening, mouse models, RNA sequencing, and detailed molecular analyses provides an interesting multi-dimensional view of the previously unknown regulatory mechanism of insulin mRNAs. While the findings are significant, some aspects of the molecular mechanism and interactions would benefit from additional experimental support to fully substantiate the authors' conclusions under physiological conditions.

Major Points

1. Overexpression vs. Endogenous Function: An important consideration is whether the overexpressed TENT5A accurately represents endogenous function. The difference between reported nuclear localization in other cell types and the observed cytosolic localization in this study might benefit from further exploration. Additional experiments comparing endogenous and overexpressed TENT5A would strengthen the conclusions.
2. Evidence for Direct Mechanisms: The current data and computational predictions provide valuable insights into potential mechanistic relationships. Additional evidence demonstrating that TENT5A directly mediates the observed effects through its polyadenylation activity, rather than through indirect pathways affecting transcription, export, or deadenylation, would enhance the mechanistic aspects of the study.
3. Data Visualization and Statistical Interpretation: Some figures could benefit from alternative visualization approaches and additional statistical analyses, particularly when examining distributions of poly(A) tail lengths and establishing relationships between poly(A) changes and transcript abundance.
4. Protein-Protein and Protein-RNA Interactions: The proposed model involving TENT5A, FNDC3, and PTBP1 presents an intriguing mechanism. Further experimental evidence for direct interactions, including approaches that complement the overexpression systems, would strengthen this aspect of the work.

Specific Comments

1. Supplementary Materials: It appears that Figures S3-S5 may have been replaced with Figures 3-5 in the submission, which limits evaluation of some supplementary data.
2. Expression Quantification (Line 210): Quantifying TENT5A overexpression relative to endogenous levels would provide helpful context for interpreting the physiological relevance of the observations.
3. Structural Prediction Considerations (Lines 214-215): The manuscript would benefit from acknowledging limitations of AlphaFold3 for RNA-protein docking predictions. Reference 34 provides good support regarding the D125/D127 residues.
4. Subcellular Localization (Line 220): The difference between the predominantly cytosolic localization observed in this study and the nuclear localization reported in ProteinAtlas for other cell types would be worth addressing (relates to Major Point 1).
5. WT vs. M1 Comparison (Line 226): The analysis comparing WT and M1 effects could be expanded. The observation that M1 shows effects in the same direction as WT (Fig. 3h), albeit weaker, is interesting since catalytically inactive mutants often act as dominant negative factor of endogenous proteins. This represents an opportunity for additional mechanistic insight.
6. Distribution Analysis (Lines 234-235): When interpreting poly(A) length changes, considering statistical regression toward the mean may be valuable. A scatter plot comparing control and WT distributions might provide additional insights beyond the current presentation.
7. Selection Criteria (Figure 3d): The basis for selecting the displayed secreted and non-secreted proteins would be helpful to include to provide context for interpretation.
8. Visualization Approach (Figure 3g): For highly expressed mRNAs like Ins1 and Ins2, a histogram with regular intervals might demonstrate the effects more clearly than the current kernel density plot.
9. mRNA Stability Analysis (Figures 3j-k): The fluctuations at 4h and 8h after Actinomycin D treatment are notable. Considering the potential limitations of Actinomycin D for measuring long half-life mRNAs (>24h), including possible cellular effects that could influence the results, would provide additional context.
10. Data Relationship Visualization (Lines 276-278): A scatter plot showing poly(A) length changes versus RNA abundance changes could further illustrate the relationship between TENT5A activity and transcriptomic changes. This might also provide insights into the observed negative correlations.
11. Enrichment Data (Figure 4l): Including enrichment levels alongside p-values would enhance the interpretability of the pathway analysis.
12. Comparative Presentation (Line 306-307): Additional explanations and including both WT and M1 data in Figure 4p would facilitate comparison.
13. Validation Approach (Figures 5a-g): Complementing the current approach with validation using endogenous TENT5A pull-down could provide additional confidence that the overexpression system reflects native interactions.
14. Interaction Evidence (Figures 5d-l): The evidence for interactions among TENT5A, PTBP1, and FNDC3 could be strengthened with approaches complementary to overexpression, such as examining poly(A) length changes in Ins1's PTBP1 binding site mutant or using in situ proximity labeling techniques.
15. Localization Patterns (Figure S5b): Exploring whether the observed localization pattern reflects cell-type specific variation, tag influence, or other factors through comparison with endogenous TENT5A in the same cells would be informative.

Reviewer #3

(Remarks to the Author)
The study by Silvia et al. investigates the role of RNA-binding proteins in beta-cell function and identifies Tent5a as a crucial regulator of insulin mRNA stability through non-canonical polyadenylation, an understudied mechanism in beta-cell compensation. The authors present functional siRNA screening and FLAM-seq experiments that provide a strong foundation for their study. Notably, they establish a novel and well-rationalized connection between ribosome collisions, p38 MAPK signaling, and Tent5a activation. The manuscript is structured logically, progressing from screening and validation to mechanistic insights and functional implications. The findings also align with prior research on RNA-binding proteins in beta-cell function and provide mechanistic insights into beta-cell adaptation during compensation.
While the manuscript is compelling, it requires some work to enhance clarity, mechanistic depth, and especially translational relevance:
1. While the study shows FNDC3B is crucial for ER localization and polyadenylation, the exact functional consequence of PTBP1 interaction remains unclear. Does PTBP1 only localize insulin mRNA to the ER, or does it actively modulate polyadenylation efficiency?
2. What regulates Tent5a expression and stability? Given that polyadenylation increases insulin mRNA stability, does this create a self-regulatory loop affecting Tent5a expression?
3. The study focuses primarily on beta-cell compensation in mouse models, and it will be important to show how these findings translate to the progressive failure observed in T2D patients. Please plan human islet studies.
4. The study would benefit from a more direct human data analysis (e.g., from published RNA-seq/single-cell-seq datasets)
5. How does overexpression of TENT5A in decompensated islets suggest a viable therapeutic intervention?
6. Please add a final clinical relevance section in the Discussion to discuss potential translational approaches (e.g., pharmacological activation of TENT5A).
7. The role of PTBP1 in insulin mRNA regulation has been previously linked to stability control rather than localization. How does this model integrate PTBP1’s known function with its interaction with TENT5A?
8. Please add a final schematic/working model showing how TENT5A, FNDC3B, and PTBP1 cooperate to regulate insulin mRNA polyadenylation, potential feedback loops, and crosstalk with canonical polyadenylation.
9. The RNAi screen description in the results section contains redundant methodological details that can be removed. It is best suited to be expanded in the Methods section.

Version 1:

Reviewer comments:

Reviewer #1

(Remarks to the Author)

The work by Silva et al establish TENT5A as a regulator of insulin mRNA polyA-tail lengths. The work also provides results of a RPB screen directed at listing factors essential for regulation of insulin production. Overall this is an interesing work, and it has improved upon revision.

Figure 1 and associated text - The screen of RPB protein function in insulin and glucose level regulation is valuable in itself. While the figures highlight the detection of Tent5a in various steps of the screen the text does not mention it at all, which is very confusing.

Figure 5j - bended 'lorem' text appears on one of the bars on the graph.

Figure 6h - please show a longer exposure of anti-HA blot. There is hardly any signal in the elution for WT TENT5A. Also the coIP does not contain a negative control (no anti-PTBP1 antibody on TENT5A-HA, so its hard to judge the background signal for TENT5-A-HA). There is anothe such blot in the supplement.

Figure S6g - control of FNDC3a/b knock-down in whole cell lysates. The blots displayed hardly show signal in control cells. To my teste its because the siRNAs against FNDC3a/b are not very efficienct. It happens, and in such cases quantifying the blot shown along with additional replicates (authors likely have many, so probably no need to pipet) ensures that siRNAs really do work. Consequently, it is very hard to judge the efficiency of the FNDC3a/b knock-down and as consequence evaluate the validity of the ensuing results.

Figure 6c and 6i - no basic statistics are given for AF3 modeling (position error, pTM and ipTM). I re-run the human and mouse protein with AF3 and the obtained models look slightly different. FNDC3B/Fndc3b are modeled as an elongated structure, rather than ring shaped. The ipTM = 0.51 pTM = 0.35 for mouse and ipTM = 0.53 pTM = 0.37 for human, so the overall structure is not super solid though the interace between both proteins seems to be plausible. Indeed in the human model TENT5A is placed in the same position in all models, which is encouraging. I wonder if some additional modelling was performed to achieve the ring shaped structure of FNDC3B? If so it should be described in material and methods. Also I could not find any reference to the proceedure in M&M.

Just on a personal level the manuscript itself is packed with data, and authors go from one result to another scarcely cocluding or interpreting results. Nor do they give motivation for performing the next analyses. I find the text hard to follow, and think that with extra editorial work it could be better appreciated, which should increase its citation.

Reviewer #2

(Remarks to the Author)
The authors have substantially strengthened the manuscript and now present a coherent, well-supported case for the central claims. A few points were addressed indirectly, likely reflecting antibody limitations and experimental complexity. In particular, endogenous protein localization and direct protein–protein interactions were not sufficiently demonstrated, and metabolic-labeling alternative strategies to prolonged shutdown of transcription by actinomycin D treatment were not performed. However, the newly added functional and quantitative data reduce their need and support the overall conclusions. Given the breadth of new analyses, the evidence seems to be sufficient for publication. I recommend softening the expressions in text somewhat that implies detailed molecular mechanism or endogenous complex formation. I support publication, provided the remaining comments from other reviewers are resolved.

Reviewer #4

(Remarks to the Author)
Comments for Author
Upon editorial request, I have reviewed this manuscript and primarily focused on the authors' reply to original reviewer 3's comments. The authors have substantially revised the manuscript and addressed the majority of the concerns raised in the initial review. The new data and clarifications strengthen both the mechanistic framework and the translational context of the study. Below I provide detailed comments on each point and a few remaining suggestions that could further improve clarity and impact.

Major points.
1. The new data (page 14, Fig. S6j) showing that PTBP1 knockdown does not affect Tent5a-dependent insulin mRNA poly(A) tail length, whereas FNDC3B remains required, is helpful. It supports the conclusion that PTBP1's stabilizing role is largely independent of poly(A) tail elongation. 1) I would recommend that the authors explicitly state in the Results/Discussion that:
PTBP1 is not required for Tent5a-mediated polyadenylation, and that its stabilizing effect is likely mediated via an alternative mechanism (e.g. 3′UTR binding and protection from decay), which remains to be fully elucidated.

2. The authors examined Tent5a and Tent5c poly(A) tail lengths in the FLAM-seq data and did not observe significant changes upon Tent5a overexpression. This is a reasonable approach to test for a simple auto-regulatory loop via tail extension. I appreciate the link back to the SignalP-TM enrichment analysis, which is consistent with Tent5a/C preferentially targeting transcripts encoding secreted/ER-targeted proteins. I would only suggest that the authors ensure that this negative result is briefly and clearly described in the main text (not exclusively in the Supplement), as this directly answers an explicit mechanistic question.

3. The new experiments in "healthy" human islet microtissues (hIsMTs) cultured under glucotoxic conditions and infected with AAV–TENT5A are a strong addition. The observation that a ~2-fold increase in TENT5A expression enhances insulin content under stress, but not in standard culture conditions, is particularly compelling and directly addresses the translational concern. It would be helpful to clearly state (e.g. in the Results) whether the increase in insulin content is accompanied by changes in GSIS and how these changes compare between basal and stimulated states.

4. The entire mechanistic framework presented in the manuscript relies on INS-1E cell-based perturbations and ex vivo islet assays. While these experiments are valuable, they remain insufficient to establish the physiological relevance of Tent5a in β-cell function and compensation. Given that the authors propose Tent5a as a key regulator of insulin mRNA stability and β-cell adaptive capacity, in vivo loss-of-function evidence is essential. I would expect genetic validation, ideally through a β-cell–specific Tent5a knockout model (βTent5aKO), to substantiate the central claims of the study.

Version 2:

Reviewer comments:

Reviewer #1

(Remarks to the Author)
During the second round of revision, Silva et al. have addressed most of my concerns:

Q1. The information regarding TENT5A has now been appropriately included in the section describing the screen (Fig. 1 data).

Q2. The “lorem” text has been removed from Fig. 5j.

Q3. The coIP-WB in Fig. 6h has been improved. However, as a minor editorial comment, the labels remain slightly misaligned and should be corrected for clarity.

Q4. The blot demonstrating the efficiency of siRNAs against FNDC3a/b in Fig. S6g has been improved.

Q5. The AlphaFold 3 model statistics are still not included in the main text or in the relevant figure panel. Additionally, the promised dedicated section in the Methods chapter describing the AlphaFold 3 analysis could not be identified. This information remains necessary for proper evaluation of the structural modeling.

Thus Silva et al. have addressed most of my questions. I recommend acceptance on condition that the Methods sections is revised to include a clear description of AlphaFold 3 modeling.

Reviewer #4

(Remarks to the Author)
The authors have clarified the relationship between PTBP1 and Tent5a-mediated polyadenylation, and the additional experiments using human islet microtissues strengthen the translational relevance of the study. The revised model describing the roles of Tent5a, FNDC3B, and PTBP1 is also helpful for understanding the proposed mechanism.

While genetic in vivo validation (e.g., β-cell–specific Tent5a knockout models) would provide a definitive test of the proposed physiological role of Tent5a in β-cell compensation, the current study already provides substantial mechanistic and functional evidence across multiple experimental systems.

Overall, the revisions satisfactorily address the major concerns raised previous reviewer 3, and the manuscript now provides a clearer mechanistic framework linking cytoplasmic polyadenylation to beta-cell compensation.

Open Access This Peer Review File is licensed under a Creative Commons Attribution 4.0 International License, which permits use, sharing, adaptation, distribution and reproduction in any medium or format, as long as you give appropriate credit to the original author(s) and the source, provide a link to the Creative Commons license, and indicate if changes were made.
In cases where reviewers are anonymous, credit should be given to 'Anonymous Referee' and the source.
The images or other third party material in this Peer Review File are included in the article’s Creative Commons license, unless indicated otherwise in a credit line to the material. If material is not included in the article’s Creative Commons license and your intended use is not permitted by statutory regulation or exceeds the permitted use, you will need to obtain permission directly from the copyright holder.
To view a copy of this license, visit https://creativecommons.org/licenses/by/4.0/

Point-to-Point Response to reviewers' comments
We thank the reviewers for taking the time to read and comment on our manuscript. We have been able to address all of the reviewers' concerns by performing many more experiments, which has led to an improved manuscript. Please find below our response to the comments (in blue). Please note that Figures for Reviewers only, figure legends and references are at the end of our point-to-point response.

Reviewer #1 (Remarks to the Author):

The study by Silva et al. identifies a role of TENT5A non-canonical polyA polymerase in regulation of insulin production in mouse pancreatic cell line model. The authors perform a screen of RNA binding proteins looking for factors relevant to insulin production. This alone is a remarkable work, and should be a usefull resource for other researchers. Next, using two pancreatic disease models they identify Tent5A as the RNA binding protein with most altered abundance. Since Tent proteins have signatures of non-cannonical polyA polymerases the authors evaluate both pA-tail lengths and mRNA abundance to find that alterations in Tent5A levels are connected to changes in abundance and adenylation of mRNAs involved in insulin biogenesis, which they confirm on a protein level. Finally, authors show taht TENT5A interacts with FNDC3B; a protein associated with the ER. They show that abolishing Tent5A association with FNDC3B results in alleviation of increased insulin production observed upon Tent5A overexpression.

Altogether, the study seems a comprehensie and engaging work, and I would like to see it published in Nature Communications. However, my overall impression is that authors have too hastingly prepared the submission, so that some aspects of the publication cannot be appreciated, whereas others are outright defective (in terms of presentation), potentially leading to readers mistrust in results. This is based on following observations:

- No PRIDE submission of raw data is listed in the manuscript.
The proteomic data has been submitted and is available through the PRIDE Accession no. PXD066929. The dataset has been published as of August 2025. The specific link is:
PRIDE: https://www.ebi.ac.uk/pride/archive/projects/PXD066929

- No submission of RNA-seq and FLAM-seq raw data is prepared. Material and methods state simply 'accession number pending'.
Both RNA-seq and FLAM-seq raw data have been released and are available for the reviewer. SRA Accession numbers for both RNA-seq and FLAM-seq is PRJNA1267696. The datasets have been published as of August 2025. The specific links is:
SRA: https://www.ncbi.nlm.nih.gov/bioproject/PRJNA1267696

- Figures S3 and S4 are missing from the supplementary figure file! Instead Figs 3 and 4 are repeated. This precludes the evaluation of large segments of the main text.
We apologize that there was an error in labeling the file, leading to a manuscript submission with missing Supplementary Figures S3 and S4. The revised manuscript has the complete set of Figures (which are now **Figure S3** and **S5**).

****

Major general, and conceptual remarks:

General formal remark. Starting from fig. 3 the authors are a bit dismissive about the cell line type in which they perform their experimesnts. the M&M section states several mouse and human cell lines, so it is important to inform the reader what the model organism is. In this context it would be helpful to improve the nomenclature of genes, as most readers are accustumed to Tent5A designating a mouse protein and TENT5A a human one. For example such inconsistencies are in Fig.2a and 2b and other places.

We are clearly indicating in the text as well as in the figure legends if experiments were performed in a rodent cell line (INS-1E), human cell line (EndoC), mouse pancreatic islets or human microtissues. Furthermore, to better clarify the cell identity used for Tent5a WT, or Tent5a M1 over-expression, we introduce these terms explicitly on page 8, where we first describe the generation of the doxycycline-inducible INS-1E cells.

We have modified all gene and protein names to match the conventional formatting style. Rat and mouse gene and RNA transcript names are italicized and in proper case (ie. *Ins1*), while human ones are in upper case (ie. *INS*, *TENT5A*). Protein names are non-italicized and in either proper case (rodent, ie. Pdia6, Tent5a) or upper case (human, PDIA4, TENT5C).

To validate the proposed recruitment of TENT5A via PTBP1, did the authors try to show that in conditions of PTBP1 silencing target mRNAs do not associate with TENT5A; eg. using RIP-qPCR or simply looking at pA-tail lengths the same manner as they did for FNDC3A (Fig. 5h)? Alternatively, was the pirimidine rich segment of INS1 RNA ever deleted and shown to impact pA-tails (even as transfected constructs)? Perhaps it's worth to consider performing those experiemnts? Do other TENT5A targets also possess such motifs?

We attempted to perform CLIP experiments to capture the direct interaction between Tent5a and its target mRNAs. Unfortunately, both techniques did not yield sufficient cross links, most likely because the lack of RNA-binding domain in Tent5a and a weak and transient association of the poly(A) tail with Tent5a, which makes UV exposure insufficient to crosslink adenosine to protein (the most common nucleosides in RNA-protein crosslinking are uridine and guanosine, and to a lesser extend cytidine (Feng *et al.*, 2024, Knorlein *et al.*, 2022).

Similarly, we achieved poor yield of RNA with immunoprecipitation of Tent5a, which again likely reflects the fact that Tent5a is not a canonical RNA binding protein with a defined binding domain, and interactions of the poly(A) tail may be weak and transient.

In addition, we performed FLAM-seq with *Ptbp1* silencing and Tent5a WT over-expression, which revealed that knockdown of Ptbp1 did indeed decrease *Ins1* transcript levels, thereby confirming previous works on Ptbp1 (Knoch *et al.*, 2014, Knoch *et al.*, 2004), however the poly(A) tail length did not shift. These results suggest that Ptbp1, while it may associate weakly with Tent5a, it is essential for maintaining mRNA abundance, however its function is independent of Tent5a activity.

A model scheme would be perfect to fully understand the proposed working model.

A model has been added to the revised manuscript and is shown in a new **Fig. 6j**.

****

Major remarks specific to data:

Figure 2a and 2c -> the opposite change in Tent5a expression 'B6-0b/ob vs B6-ob/+' compared to 'Akita vs WT' is one of the criteria of selecting the protein for further studies. The reader would like to

appreciate if more such proteins were selected in the screen. Perhaps to this end a scatterplot comparing the significant RPBs fold change (eg. on x-axis 'B6-0b/ob vs B6-ob/+' compared to 'Akita vs WT' on y-axis) would be helpful. Was the decrease in Tent5A protein levels also confirmed by western blot? Shouldn't it be shown together with the western on panel 2b?
We have included the scatter plot the reviewer suggested (see new **Fig. S2h**) to highlight the unique position Tent5a holds in being the only candidate with opposite and significant changes in these two differential expression analyses.
By Western blot, we identified a reduction in Tent5a protein in islets of Akita mice, consistent with the findings from RNAseq analysis. This data is provided as a new **Fig. S2f,g**.

Figure 2j -> quote lines 190-191 'Furthermore, anisomycin increased phosphorylation of p38 MAPK at Thr180/Tyr182, which is consistent with activation of p38 MAPK during ribosome collision (Fig. 2j) 33' -> looking at the western blot I cannot see any change in phospho-p38 levels. Western is not very quantitative, so the densitrometry data holds the conclusion together. However, to convince the reader I would ask for 6 more replicates and some statistics. Same applies to Fig 4m and 4p. Importantly, the densitrometry protocol is not provided in material and methods. Please include it in the revised version.
The experiment presented in **Fig. 2j** was repeated and the densitometry quantification is presented in **Fig. 2k**.

Furthermore, the blots presented in new **Fig. 5i,l** (formerly in Figure 4), now have accompanying densitometry figures (**Fig. 5j** and **5m**, respectively) to better illustrate the changes in protein abundance that we observe through Tent5a WT expression.
Finally, the methods section pertaining to Western Immunoblotting has an additional paragraph detailing the densitometry approach (see page 33, under the heading “Western blotting”).

Fig. 3j -> the error bars seem off. Some are not centered around data points.
We regret the confusion the reviewers encountered with these figures. Originally, the error bars were unidirectional and uniformly black. The overlap in error bars with data points created the illusion that they were uncentered. To correct this visual distortion, we have color-matched the now bidirectional error bars with the datapoints and lines of best fit for all figures arising from Actinomycin D experiments. This applied to **Fig 3j,k** as well as **Fig S3o-r** and the new panels in **Fig 4k,l**.

Fig. 4k and related text -> text quote 'in addition, Tent5a WT induced an increase in cellular ATP levels consistent with enhanced mitochondrial ATP production (Fig. 4k) 52-54'. Quote is inconsitent with the content of the figure panel (legend quote: '(k) Western blots depicting the protein levels of DDOST, NUCB2 and GNAS in uninduced compared Tent5a WT or Tent5a M1 overexpressing cells. n=3.'). Nontheless, how is ATP level relevant to a simple enzyme-substrate (TENT5A-mRNA) recruitment mechanism? ATP is abundant , and an increase in ATP levels might just be a means to support incresed protein production and secretion.
The data corresponding to the text in the Results section is now presented in **Fig. 5o**. The Figure legend now also matches this designation. We thank the reviewer for identifying this discrepancy.

Indeed, we agree with the reviewer that it is unlikely that the increased ATP needed by Tent5a to perform its enzymatic activity on its targets is the only demand the cell is trying to meet. Rather, the increased insulin production (protein translation is the most energy-requiring cellular process [see Buttgereit and Brand, 1995, Rolfe and Brown, 1997, Boland *et al.*, 2017]), and other ATP-dependent enzymatic processes (ie. protein disulfide isomerase activity) may place a greater pressure on the

beta cell to initiate compensatory mechanisms such as the upregulation of mitochondrial genes and ultimately raise the cellular ATP content, as we observed.

Fig. 5a - Unless I missed it somewhere in the files, it would be helpfull if the authors provided an excell file summing up the hits obtained in mass spectrometry analysis together with a list of significantly enriched proteins.
This data is provided in **Table S10**, presented as an excel sheet containing two sheets, one for the hits associated with Tent5a WT and the other Tent5a M1.

Fig. 5j -> IP using PTBP1 as bait, please show the bait protein. It is impossible to evaluate the result unless the reader sees equal amounts of bait protein.
Fig. 6h and **Fig. S6g** have been updated to include the Western blot of the bait, Ptbp1.

Figure S5 - IF data. If the dependency of TENT5A localization to ER on Fndc3a was assessed only by inspecting single images, and using the lines drawn arbitrarily then the conclusions put forward seem not convicing. Please use an appropriate protocol to quantify several replicates and provide statistical evaluation of the data. In any case, even if colocalization is not fully abolished, does this really imact the mechanistic model?
The Tent5a association to the ER was based on colocalization analysis of over 80 cells from 9-10 images per condition (**Fig. S6d**) in addition to immunoprecipitation experiments with Fndc3 proteins. The line plots were generated to provide an additional interpretation of the images provided, not to draw further conclusions, much like in other publications in *Nature Communications* featuring cellular imaging data (Fig. 1g-I in M'Saad and Bewersdorf, 2020, Fig. 4c in Kwon *et al.*, 2015 and Fig 3f in Tran *et al.*, 2022). Here, the intensities of the line plot were normalized and statistical analysis to measure the Spearman correlation coefficient was applied to better compare the degree of correlation as was previously performed (see Plissonnier *et al.*, 2016, Yamada *et al.*, 2011, Filippi *et al.*, 2017). The methods section has been updated to include this protocol (See bottom of Page 30).

Together, we provide firm evidence for the proposed model using an unbiased immunoprecipitation experiment followed by proteomic analysis (**Fig.6a**), validation experiments (**Fig. 6b**), AlphaFold3 predictions (**Fig. 6c**) and subsequent experiments with domain truncation constructs (Fig.**6d**, **6e**, **S6h**), and genetic experiments showing that the interaction between Tent5a with Fndc3 is responsible for the polyadenylation activity (**Fig. 6f**, **6g**, **S6g**, new **Fig. S6j**).

Figure S5g - essentially same remark as Fig.5j, additionally the legend quote (line 1254-1259) does not reflect the content of the figure: '(g) Western blot for the HA-tag following immunoprecipitation of PTBP1 in uninduced control cells or those induced with doxycycline to express Tent5a WT transfected with either scrambled or Fndc3a and 3b targeting siRNA. Data are presented as mean ± s.d. in a, d and f. One-way ANOVA followed by Dunnett's Multiple comparison testing used to analyze d. Two-Way ANOVA followed by Šídák multiple comparison testing used to analyze f.' -> The panel shows a HA-tag western blot. It is not clear what the HA-tagged protein is; was it TENT5A?. Furthermore the bait PTBP1 protein is not shown. Please clarify this panel and its legend.
The antibodies recognize the HA-tagged Tent5a. The legends for **Fig. 6** and **S6** have been modified for improved clarity (Pages 45-46). Throughout the paper, the HA-tag has only been associated with Tent5a WT or its variants (M1 catalytically inactive mutant, or truncated forms designated ΔN or ΔC). The bait in this case targets Ptbp1 (as indicated in the second line of the panel: IP: Ptbp1).

Supplementary Table S11 seems damaged?
Table S11 was fixed and is now presented as a PDF.

****

Minor:

lines 366-367 repetition: 'Our systematic screens suggest that RBP mainly are mainly positive regulators (...)'
This was corrected.

Naming the result section as 'main' seems unorthodox?
We have changed the heading from "Main" to "Introduction". The Results sections has and will continue to be identified as "Results".

Fig. 5j -> anit-PTBP1, should be 'anti-'
This was corrected.

Reviewer #2 (Remarks to the Author):

This manuscript presents an intriguing investigation into the mechanisms underlying beta cell compensation in response to metabolic stress. Through a comprehensive RNAi screen of RNA-binding proteins, the authors identify Tent5a as a critical regulator of insulin production and secretion in pancreatic beta cells. The work demonstrates that Tent5a expression is upregulated during beta cell compensation in obesity models and functions by elongating poly(A) tails of insulin mRNAs, thereby enhancing their stability. The authors further elucidate a mechanistic model whereby Tent5a is tethered to the endoplasmic reticulum via FNDC3 proteins, with PTBP1 facilitating the interaction with insulin mRNA.

The study tackles an important molecular mechanism that improves our understanding of post-transcriptional regulation in beta cell function and potential pathways for therapeutic intervention in diabetes. The multi-faceted experimental approach combining RNAi screening, mouse models, RNA sequencing, and detailed molecular analyses provides an interesting multi-dimensional view of the previously unknown regulatory mechanism of insulin mRNAs. While the findings are significant, some aspects of the molecular mechanism and interactions would benefit from additional experimental support to fully substantiate the authors' conclusions under physiological conditions.

Major Points

1. Overexpression vs. Endogenous Function: An important consideration is whether the overexpressed TENT5A accurately represents endogenous function. The difference between reported nuclear localization in other cell types and the observed cytosolic localization in this study might benefit from further exploration. Additional experiments comparing endogenous and overexpressed TENT5A would strengthen the conclusions.
The reviewer's questions about the role of Tent5a in the nucleus vs cytoplasm raises an interesting topic. We hope that the correct inclusion of **Fig. S3** (specifically **S3e** and **S3f**) demonstrating a predominantly cytoplasmic localization for Tent5a, demonstrating Tent5a functions as a non-canonical cytoplasmic polyadenylase.

To address the role of endogenous Tent5a, we developed a loss-of-function CRISPR model. The results show that depletion of Tent5a leads to the opposite phenotype than the overexpression data. These findings demonstrate that cytoplasmic polyadenylation of Ins transcripts is not only relevant in activating conditions (i. metabolic stress) but contributes to the maintenance of basal insulin mRNA stability and expression, The new data are shown in **Fig. 4g-m** and **Fig. S4k,l** and described in the abstract and result section starting on pages 10,11).

2. Evidence for Direct Mechanisms: The current data and computational predictions provide valuable insights into potential mechanistic relationships. Additional evidence demonstrating that TENT5A directly mediates the observed effects through its polyadenylation activity, rather than through indirect pathways affecting transcription, export, or deadenylation, would enhance the mechanistic aspects of the study.
To address this point, we have performed additional experiments to probe whether Tent5a expression impacts the transcriptional activity of insulin gene (via luciferase promoter analysis; **Fig. S4b,c**).

To test whether Tent5a over-expression impacts processes like nuclear mRNA export, we looked at the regulation of genes associated with these processes in the RNAseq dataset of Tent5a WT over-expression. For nuclear export, we analyzed nuclear export receptors (*Xpot, Xpo1, -6, -7, -4, -5, Cse1l*), Trex complex members (*Thoc1, -2, -3, -5, -6, -7, Ddx39b, Alyref*), mRNA nuclear export machinery (*Nxf1/2, Nxt1*), Ran GTPase cycle components (*Ran , Rangap1, Ranbp1/2*), nuclear pore complex nucleoporins (*Nup35, -50, -54, -58, -62, -93, -98, -153, -188, -205, -214*) and tRNA and rRNA export factors *(Elac2, Rcc1, -1l, Nmd3, Pa2g4*). Of all these genes, only *Ddx39b* was modestly regulated (Log_2FC of -0.37, p-value = 0.01, FDR = 0.13). Lastly, the fact that Tent5a lacks a nuclear import sequence, makes it unlikely to have an impact there.

To explore if Tent5a has an effect on mRNA deadenylation we analyzed genes associated with deadenylation, including members of the Ccr4-Not complex (*Cnot1, -2, -3, -4, -6, -6l, -7, 8, -9, -10, -11*), Pan2-Pan3 complex members (*Pan2* and *Pan3*), Parn-type deadenylases (*Parn, Noct, Angel1, -2*) and other mRNA decay-linked deadenylases (*Toe1, Mtpap, Zc3h14*). None of these exhibited statistically significant regulation. The complete differential gene expression analysis results are provided in **Table S9**.

We also investigated if Tent5a may affect insulin gene transcription and thereby contribute to *increased* mRNA levels. We transfected a luciferase reporter driven by the human *INS* promoter in INS-1E cells that was found to be glucose responsive as expected (see new **Fig. S4b**). Using this construct, we measured no difference in luciferase activity between uninduced cells (Control) and those overexpressing wildtype TENT5A (see new **Fig. S4c**). These data, together with the cytoplasmic localization, make it unlikely that Tent5a directly promotes insulin gene transcription.
Together, we believe that we provide compelling biochemical, genetic and structural evidence that the main observed effects relating to insulin are due to Tent5a's polyadenylation activity. These results are now further strengthened by additional experiments on *INS* promoter activity and cells with loss-of-Tent5a function. model would suggest that the main observed effects we observed related to insulin are due to Tent5a's polyadenylation activity.

3. Data Visualization and Statistical Interpretation: Some figures could benefit from alternative visualization approaches and additional statistical analyses, particularly when examining distributions of poly(A) tail lengths and establishing relationships between poly(A) changes and transcript abundance.

We thank the reviewer for this suggestion and have made modifications to **Fig. 3g** (which informed our decision to present new data in **Fig. 4j** and **S6h**) and introduced new visualizations of data (**Fig. S2h** and **S3g**) that will help readers understand the data better.

4. Protein-Protein and Protein-RNA Interactions: The proposed model involving TENT5A, FNDC3, and PTBP1 presents an intriguing mechanism. Further experimental evidence for direct interactions, including approaches that complement the overexpression systems, would strengthen this aspect of the work.
We aimed to address the importance of Ptbp1 using FLAM-seq analysis along with Fndc3a/b knockdown. These analyses confirmed the importance of Fndc3 proteins in enabling Tent5a activity to elongate the poly(A) tail of *Ins1*.
In contrast while we observed a notable drop in Ins1 transcript abundance when we silenced *Ptbp1*, a similar shift in poly(A) tail length was not identified. These results, while validating the importance of Ptbp1 in the maintenance of insulin transcript, suggests that its association with Tent5a does not impact poly(A) tail length dynamics.

Specific Comments

1. Supplementary Materials: It appears that Figures S3-S5 may have been replaced with Figures 3-5 in the submission, which limits evaluation of some supplementary data.
The supplemental figures have been properly included in this submission.

2. Expression Quantification (Line 210): Quantifying TENT5A overexpression relative to endogenous levels would provide helpful context for interpreting the physiological relevance of the observations.
We have quantified Tent5a over-expression relative to endogenous levels in the over-expression system and have presented this as a **new Fig. S3c**.

3. Structural Prediction Considerations (Lines 214-215): The manuscript would benefit from acknowledging limitations of AlphaFold3 for RNA-protein docking predictions. Reference 34 provides good support regarding the D125/D127 residues.
We agree and acknowledge that AlphaFold 3 lag behind in terms of reliability for RNA structures and, in particular, protein-RNA complexes, especially for non-canonical interactions such as unusual base pairs. This is now mentioned in the Results section (Page 14). Rather, we viewed the AlphaFold 3 predictions as a tool for hypothesis generation.
Lines 215 – 218 have been updated to better emphasize the predictive analysis presented in Kuchta *et al.*, 2016 and state that AlphaFold3 supported this prediction. The data presented in **Fig. 3**, **4** and **5** validate the prediction.

4. Subcellular Localization (Line 220): The difference between the predominantly cytosolic localization observed in this study and the nuclear localization reported in ProteinAtlas for other cell types would be worth addressing (relates to Major Point 1).
The ProteinAtlas images for the Tent5a entry were performed with an antibody (HPA051067) that could not detect protein by Western blot, therefore the data should be viewed with caution. Furthermore, the images of the kidney with antibody (HPA067140) show a predominantly cytosolic localization, which is in line with the current literature of Tent5 proteins (see Gewartowska *et al.*, 2021, Fucci *et al.*, 2020).

We are showing in a new **Fig. S3f** the subcellular localization of Tent5a in pancreatic beta cells, demonstrating that Tent5a is predominantly located in the cytoplasm. In addition, we performed biochemical fractionation studies (see new **Fig. S3e**) that are consistent with these results.

5. WT vs. M1 Comparison (Line 226): The analysis comparing WT and M1 effects could be expanded. The observation that M1 shows effects in the same direction as WT (Fig. 3h), albeit weaker, is interesting since catalytically inactive mutants often act as dominant negative factor of endogenous proteins. This represents an opportunity for additional mechanistic insight.
We used FLAM-seq to test this hypothesis where we compared the poly(A) tail length profile for *Ins1* in one sample each of Tent5a M1- and WT- over-expression against one sample of uninduced INS-1E cells (**Figure 1 for reviewers only**). This analysis showed that *Ins1* from Tent5a M1 matched more with the Uninduced sample both in terms of the abundance (y-axis) but also the poly(A) tail length distribution (x-axis). Further analysis of the Northern blot revealed that a 1° clockwise rotation of the Northern blot aligns the center of the bands associated with Tent5a M1 with the Control (**Figure 2 for reviewers only**). This finding is consistent both with this FLAMseq analysis and the additional data we present in the study related to insulin mRNA stability (**Fig 3j,k**), abundance (**Fig. 3m, n**) and protein abundance (**Fig. 4a-c**). The M1 mutant does retains the third critical E201 residue unmutated, which was also identified by Kuchta *et al.*, 2016 as a critically important residue for catalytic activity. However, our work suggests that D125 and D127 are essential for polyadenylation.

6. Distribution Analysis (Lines 234-235): When interpreting poly(A) length changes, considering statistical regression toward the mean may be valuable. A scatter plot comparing control and WT distributions might provide additional insights beyond the current presentation.
We plotted the relationship between the basal poly(A) tail length and the % change in poly(A) tail length due to Tent5a WT induction for all transcripts identified by FLAM-seq and present it in **Fig. S3g**. The negative correlation matches the data we presented in **Fig. 3d**.

7. Selection Criteria (Figure 3d): The basis for selecting the displayed secreted and non-secreted proteins would be helpful to include to provide context for interpretation.
A section has been added to the materials and methods under "Data Analysis" for "Full Length mRNA Sequencing" (Page 26) to better clarify how transcripts associated with secretion were selected.

8. Visualization Approach (Figure 3g): For highly expressed mRNAs like Ins1 and Ins2, a histogram with regular intervals might demonstrate the effects more clearly than the current kernel density plot.
A new Fig. 3g is shown in the revised manuscript to reflect suggestion of the reviewer. Furthermore, new data in **Fig. 4h** and **Fig. S6j** have been presented in a similar manner.

9. mRNA Stability Analysis (Figures 3j-k): The fluctuations at 4h and 8h after Actinomycin D treatment are notable. Considering the potential limitations of Actinomycin D for measuring long half-life mRNAs (>24h), including possible cellular effects that could influence the results, would provide additional context.
When we treat INS-1E cells with actinomycin D or DMSO for 24 hours and assess cell death by Annexin V staining and FACS, we see that there is a trend towards more apoptotic cells, but only averaging ~3% (Actinomycin D) compared to 2% for DMSO (see **Figure 3 for reviewers only**). However, beyond this time, we suspect cytotoxicity will confound the effect we would aim to observe as it relates to mRNA stability. In **Fig. 3j** and **3k** along with **S3o** and **S3p**, we measured large

fluctuations at 4h and 8h between 1.0 and 1.5 relative expression levels. However, this pattern cannot be observed in transcripts with shorter half-lifes like *Memo1* and *Cep55* (**Fig. S3q** and **S3r**). We believe the patters at these early time points are due to the longer half-lives of these particular transcripts. The fact that the fluctuations with Actinonmycin D treatment appear to affect samples associated with Tent5a WT more is somewhat consistent with our original understanding how (canonical) cytoplasmic polyadenylation was originally identified to play a critical role in a narrow window of embryogenesis where transcriptional regulation is unavailable for gene regulation (see McGrew *et al.*, 1989, Hake and Richter, 1994, Paris and Richter, 1990). However, this is purely speculative and may warrant further study in other contexts, such as embryogenesis.

10. Data Relationship Visualization (Lines 276-278): A scatter plot showing poly(A) length changes versus RNA abundance changes could further illustrate the relationship between TENT5A activity and transcriptomic changes. This might also provide insights into the observed negative correlations.
We plotted the relationship suggested by the reviewer where RNA transcript abundance in uninduced INS-1E cells from RNAseq was plotted against the percentage change in poly(A) tail length due to Tent5a WT expression (**see Figure 4 for reviewers only**):

Given the extremely high levels of *Ins1* and *Ins2* transcripts (highlighted in red), the relationship plotted does not intuitively follow the distribution of transcripts in this relationship. We believe **Fig. S3h** illustrates how more abundant transcripts are more heavily represented among transcripts with elongated poly(A) tails as this plot also highlights that these transcripts often also have shorter basal poly(A) tail lengths.

11. Enrichment Data (Figure 4l): Including enrichment levels alongside p-values would enhance the interpretability of the pathway analysis.
In **Table S9**, we have created an additional sheet that contains the complete information regarding the Biological Processes Up-regulated genes over-representation analysis to complement **Fig. 5k**.

12. Comparative Presentation (Line 306-307): Additional explanations and including both WT and M1 data in Figure 4p would facilitate comparison.
The data investigating ER stress is now presented in **Fig. S5i-j**. Following the advice of the reviewer, we have compared the effect of Tent5a WT and M1 expression on phosphorylation of Eif2alpha and found no statistically significant change.

13. Validation Approach (Figures 5a-g): Complementing the current approach with validation using endogenous TENT5A pull-down could provide additional confidence that the overexpression system reflects native interactions.
Unfortunately, our custom-made TENT5A antibody was not compatible with rat lysates and therefore we were unable to perform this experiment. However, the interaction between Tent5a with Fndc3 is consistent with earlier-reported interactions between Tent5c and Fndc3 Fucci *et al.*, 2020.

14. Interaction Evidence (Figures 5d-l): The evidence for interactions among TENT5A, PTBP1, and FNDC3 could be strengthened with approaches complementary to overexpression, such as examining poly(A) length changes in Ins1's PTBP1 binding site mutant or using in situ proximity labeling techniques.

We performed FLAM-Seq to investigate whether Ptbp1 is required for Tent5a's effect on poly(A) tail length elongation. These analyses demonstrated that Ptbp1 knockdown did not adversely impact the poly(A) tail length of *Ins1*, and therefore, it may not be an essential interaction partner for Tent5a. These data are now described on page 14, and shown in a **new Fig. S6j**.

15. Localization Patterns (Figure S5b): Exploring whether the observed localization pattern reflects cell-type specific variation, tag influence, or other factors through comparison with endogenous TENT5A in the same cells would be informative.
Unfortunately, we could not perform a broader survey of Tent5a sub-cellular localization in different cell types, as our custom-made and commercial antibodies produced non-specific stainings. With respect to the tag influence, we also generated a C-terminus V5-tagged form of Tent5a which exhibit the same localization pattern as the N-terminus HA-tagged (**Fig 5 for reviewers only**).

Reviewer #3 (Remarks to the Author):

The study by Silvia et al. investigates the role of RNA-binding proteins in beta-cell function and identifies Tent5a as a crucial regulator of insulin mRNA stability through non-canonical polyadenylation, an understudied mechanism in beta-cell compensation. The authors present functional siRNA screening and FLAM-seq experiments that provide a strong foundation for their study. Notably, they establish a novel and well-rationalized connection between ribosome collisions, p38 MAPK signaling, and Tent5a activation. The manuscript is structured logically, progressing from screening and validation to mechanistic insights and functional implications. The findings also align with prior research on RNA-binding proteins in beta-cell function and provide mechanistic insights into beta-cell adaptation during compensation.
While the manuscript is compelling, it requires some work to enhance clarity, mechanistic depth, and especially translational relevance:

1. While the study shows FNDC3B is crucial for ER localization and polyadenylation, the exact functional consequence of PTBP1 interaction remains unclear. Does PTBP1 only localize insulin mRNA to the ER, or does it actively modulate polyadenylation efficiency?
In the revised manuscript we show that PTBP1 is not required for Tent5a activity, while FNDC3B is. These data suggest that PTBP1's stabilizing action on Ins transcripts in independent of poly(A) tail length. These data are now described on **page 14,** and shown in a **new Fig. S6j**.

2. What regulates Tent5a expression and stability? Given that polyadenylation increases insulin mRNA stability, does this create a self-regulatory loop affecting Tent5a expression?
We checked the poly(A) tail lengths of both *Tent5a* and *Tent5c* transcripts in our FLAMseq datasets, but neither experienced a statistically significant change upon Tent5a WT expression (**see Figures 6 and 7 for reviewers only**).
These results suggest that Tent5a does not auto-regulate its expression. This is consistent with Tent5a and Tent5c not being secreted proteins or transcripts identified with the "SignalP-TM" designation, which were more likely to be targets of Tent5a for poly(A) tail elongation (**Fig. S3i**)

3. The study focuses primarily on beta-cell compensation in mouse models, and it will be important to show how these findings translate to the progressive failure observed in T2D patients. Please plan human islet studies.

To address this question, we over-expressed TENT5A in "healthy" human islet cells re-aggregated as uniform-sized microtissues (**Fig. 4d)**. These MTs were cultured in glucotoxic conditions that partially recapitulates the progressive failure experienced by T2D patients. We assessed beta cell function by measuring insulin content and production in these tissues and found that even a modest ≈2-fold overexpression of TENT5A by AAV infection increased insulin content during stress conditions compared to control AAV infected cells. These data are now shown in **new Fig. 4d–g, and S4d–j**.

4. The study would benefit from a more direct human data analysis (e.g., from published RNA-seq/single-cell-seq datasets)
We present human RNAseq data from islets (**Fig. S2i-k**) re-analyzed from published datasets (Wigger *et al.*, 2021). In an independent analysis of published single cell sequencing data of human beta cells, where we identified genes that correlated with high or low expression levels of TENT5s (Lawlor *et al.*, 2017). Whereas no correlation was observed with TENT5A, we identified a correlation between INS and TENT5C expression, consistent with the whole human islet data (**Figure 8 For reviewers only**).

5. How does overexpression of TENT5A in decompensated islets suggest a viable therapeutic intervention?
We observed a statistically significant increase in insulin content in decompensated human islet micro-tissues with a modest 2-fold increase in *TENT5A* expression. The observation in stressed, but not "healthy" MTs demonstrates that when the highly-regulated insulin production process is impaired, cytoplasmic polyadenylation via TENT5A could be recruited to ameliorate beta cell function, potentially opening the possibility for therapeutic intervention that either increases enzymatic activity of TENT5 proteins, increase their expression or recruitment to the Fndc3 proteins at the ER.

6. Please add a final clinical relevance section in the Discussion to discuss potential translational approaches (e.g., pharmacological activation of TENT5A).
A clinical relevance section was added in the Discussion to discuss potential translational approaches of Tent5A activators and inhibitors (see page 18).

7. The role of PTBP1 in insulin mRNA regulation has been previously linked to stability control rather than localization. How does this model integrate PTBP1's known function with its interaction with TENT5A?
Our data would suggest that indeed Ptbp1's role in insulin mRNA regulation is tied to stability and mRNA abundance through a mechanism that is independent of polyadenylation. Please note that PTBP1 was only studied in the context of Tent5A activation, however, Tent5a independent roles are not the focus of this paper and beyond the scope of this study.

8. Please add a final schematic/working model showing how TENT5A, FNDC3B, and PTBP1 cooperate to regulate insulin mRNA polyadenylation, potential feedback loops, and crosstalk with canonical polyadenylation.
This model has been added. (**see new Fig. 6j**)

9. The RNAi screen description in the results section contains redundant methodological details that can be removed. It is best suited to be expanded in the Methods section.

The redundant methodological details have been removed, and the general description of the RNAi screen is summarized on pages 4,5.

Figures for Reviewers only

1

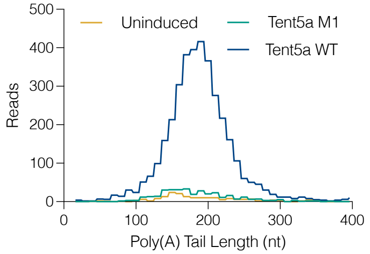

2

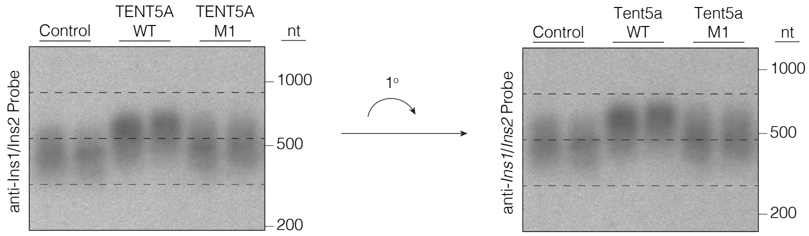

3

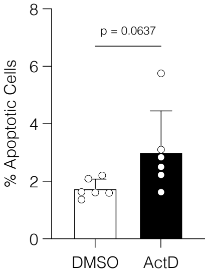

4

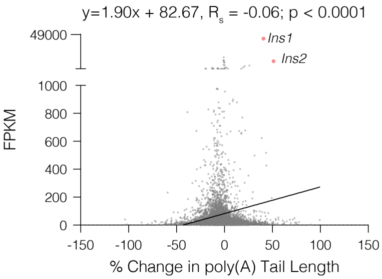

5

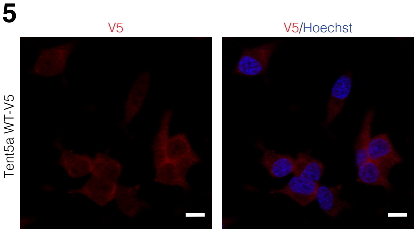

6

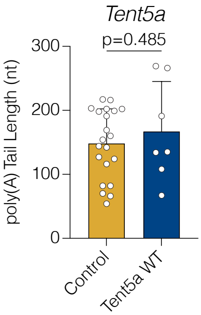

7

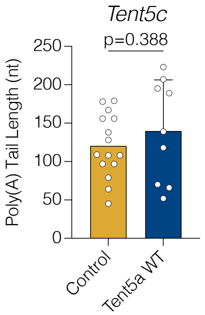

8

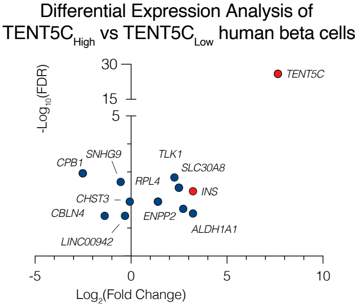

Figures for reviewers only. (**1**) Histogram of poly(A) tail length distributions of *Ins1* transcripts from uninduced Control (yellow), Tent5a WT (blue) or Tent5a M1 (teal) samples derived from FLAM-seq analysis. (**2**) Northern blot autoradiograph generated by a ^{32}P-labelled Ins1/2 probe presented in the first submission (left) and now presented in Fig. 3h of Silva *et al*. after a 1° clockwise rotation. (**3**) Percentage of apoptotic INS-1E cells stained with APC-Annexin V and Sytox Blue after 24 h treatment with either DMSO or Actinomycin D (ActD; 5 µg/mL). n=6 for each condition. (**4**) Relationship between mRNA abundance (represented as average FPKM values derived from RNAseq analysis of Uninduced Control INS-1E cells; y-axis) against the average percentage change in poly(A) tail length with Tent5a WT over-expression (derived from FLAM-seq analysis; x-axis). Each dot represents a different transcript and *Ins1* and *Ins2* are highlighted in red. Line of best fit with accompany equation, Spearman correlation coefficient and associated p-value included. (**5**) Representative immunofluorescence image of V5 (red) and Hoechst (blue) staining in Tent5a WT-V5 construct-expressing INS1E cells. Scale bar: 10 µm. (**6**,**7**) poly(A) tail lengths derived from FLAM-seq analysis of uninduced control and Tent5a WT over-expressing INS-1E cells of individual transcripts assigned to (**6**) *Tent5a* (n= 21 and 7 transcripts) and (**7**) *Tent5c* (n= 15 and 9 transcripts). (**8**) Differential Expression Analysis of cell populations with high vs low *TENT5C* expression. Transcripts with FDR values <0.05 shown against their Log_2 (fold change) between the two groups. Highlighted in red are *INS* and *TENT5C*. Data are presented as mean ± s.d. in 3, 6 and 7. Unpaired t-test used to analyze data in 3, 6 and 7.

References:

Boland, BB, Rhodes, CJ, . . . Grimsby, JS. 2017. *Mol Metab,* 6**,** 958-973.
Buttgereit, F & Brand, MD. 1995. *Biochemical Journal,* 312**,** 163-167.
Feng, H, Lu, XJ, . . . Zhang, C. 2024. *Nat Commun,* 15**,** 2279.
Filippi, BM, Abraham, MA, . . . Lam, TKT. 2017. *Cell Rep,* 18**,** 2301-2309.
Fucci, C, Resnati, M, . . . Milan, E. 2020. *Cell Rep,* 32**,** 108162.
Gewartowska, O, Aranaz-Novaliches, G, . . . Dziembowski, A. 2021. *Cell Rep,* 35**,** 109015.
Hake, LE & Richter, JD. 1994. *Cell,* 79**,** 617-627.
Knoch, KP, Bergert, H, . . . Solimena, M. 2004. *Nat Cell Biol,* 6**,** 207-14.
Knoch, KP, Nath-Sain, S, . . . Solimena, M. 2014. *Mol Metab,* 3**,** 518-30.
Knorlein, A, Sarnowski, CP, . . . Hall, J. 2022. *Nat Commun,* 13**,** 2719.
Kuchta, K, Muszewska, A, . . . Ginalski, K. 2016. *Nucleic Acids Res,* 44**,** 3534-48.
Kwon, J, Hwang, J, . . . Kim, SK. 2015. *Sci Rep,* 5**,** 17804.
Lawlor, N, George, J, . . . Stitzel, ML. 2017. *Genome Res,* 27**,** 208-222.
M'saad, O & Bewersdorf, J. 2020. *Nat Commun,* 11**,** 3850.
Mcgrew, LL, Dworkin-Rastl, E, . . . Richter, JD. 1989. *Genes & development,* 3**,** 803-815.
Paris, J & Richter, JD. 1990. *Molecular and Cellular Biology,* 10**,** 5634-5645.
Plissonnier, ML, Lahlali, T, . . . Parent, R. 2016. *PLoS Biol,* 14**,** e1002421.
Rolfe, DF & Brown, GC. 1997. *Physiological Reviews,* 77**,** 731-758.
Tran, TD, Ali, MA, . . . Luallen, RJ. 2022. *Nat Commun,* 13**,** 693.
Wigger, L, Barovic, M, . . . Solimena, M. 2021. *Nat Metab,* 3**,** 1017-1031.
Yamada, J, Nakanishi, H, . . . Jinno, S. 2011. *Neuroscience,* 182**,** 1-10.

Point by point response to the Reviewers' comments

We would like to thank all reviewers to take the time to read our revised manuscript and for their constructive comments. We have addressed all points that the reviewers raised, which, we believe, has improved the manuscript. Please find our specific response to the comments below:

Reviewer #1 (Remarks to the Author):

The work by Silva et al establish TENT5A as a regulator of insulin mRNA polyA-tail lengths. The work also provides results of a RPB screen directed at listing factors essential for regulation of insulin production. Overall this is an interesing work, and it has improved upon revision.

Figure 1 and associated text - The screen of RPB protein function in insulin and glucose level regulation is valuable in itself. While the figures highlight the detection of Tent5a in various steps of the screen the text does not mention it at all, which is very confusing.
We agree - Tent5a is now mentioned in the section where the RNAi screen is described (page 5, lines 145-148).

Figure 5j - bended 'lorem' text appears on one of the bars on the graph.
The placeholder text in Figure 5j was deleted.

Figure 6h - please show a longer exposure of anti-HA blot. There is hardly any signal in the elution for WT TENT5A. Also the coIP does not contain a negative control (no anti-PTBP1 antibody on TENT5A-HA, so its hard to judge the background signal for TENT5-A-HA). There is anothe such blot in the supplement.
We are now showing a new experiment with a longer exposure of the anti-HA in a revised Figure 6h. In the new blot, the signal in the elution for WT TENT5A is much stronger. The new experiment also contains duplicate lanes of a negative control (lanes 7&8), demonstrating that there is no protein binding to uncoupled beads.

Figure S6g - control of FNDC3a/b knock-down in whole cell lysates. The blots displayed hardly show signal in control cells. To my teste its because the siRNAs against FNDC3a/b are not very efficienct. It happens, and in such cases quantifying the blot shown along with additional replicates (authors likely have many, so probably no need to pipet) ensures that siRNAs really do work. Consequently, it is very hard to judge the efficiency of the FNDC3a/b knock-down and as consequence evaluate the validity of the ensuing results.
We have replaced the anti-Fndc3a and anti-Fndc3b with longer exposures to better assess the knockdown efficiency. We have also quantified the blot shown in Figure S6g and included additional replicates now shown as a new Figure S6h. Fndc3b knockdown alone was the least efficient. We also noted that silencing of Fndc3a resulted in significant compensation of Fndc3b. The same compensation was not observed when Fndc3b is knocked down. The quantification is shown in a new Figure S6h.

Figure 6c and 6i - no basic statistics are given for AF3 modeling (position error, pTM and ipTM). I re-run the human and mouse protein with AF3 and the obtained models look slightly different. FNDC3B/Fndc3b are modeled as an elongated structure, rather than ring shaped. The ipTM = 0.51 pTM = 0.35 for mouse and ipTM = 0.53 pTM = 0.37 for human, so the overall structure is not super solid though the interace between both proteins seems to be plausible. Indeed in the human model TENT5A is placed in the same position in all models, which is encouraging. I wonder if some additional modelling was performed to achieve the ring shaped structure of FNDC3B? If so it should be described in material and methods. Also I could not find any reference to the proceedure in M&M.
In response to the comments of reviewer 1, we have now added a materials & methods section detailing the context in which the AlphaFold models were generated, including the statistical parameters ipTM and pTM. Indeed, the prediction used to characterize the interaction with mouse Tent5a with rat Fndc3b included rat Ins2 mRNA and a single ATP molecule. In the five different models generated from this prediction, Fndc3b is consistently depicted as a ring rather than the elongated structure we also observe when modeling Tent5a with Fndc3b alone. The ring structure is similar to what we observe when rat Ptbp1 and Ins1 mRNA are included in the model (as depicted in Figure 6). It is possible that the structural prediction of Fndc3b is influenced by the presence of the mRNA. We also recognize the limitations to this prediction given that the ER itself cannot yet be included as a parameter while, in reality, it may exert a greater influence on Fndc3b structure. Nevertheless, we agree with the reviewer that it is encouraging that these *in silico* predictions and the biochemical assays appear to align with our proposed model.

Just on a personal level the manuscript itself is packed with data, and authors go from one result to another scarcely cocluding or interpreting results. Nor do they give motivation for performing the next analyses. I find the text hard to follow, and think that with extra editorial work it could be better appreciated, which should increase its citation.
We have gone through the manuscript with paying special attention if the motivation for experiments were described and if conclusions were made. Every paragraph of the result section is introduced with an introductory motivation sentence and a summarizing and concluding sentence.

Reviewer #2 (Remarks to the Author):

The authors have substantially strengthened the manuscript and now present a coherent, well-supported case for the central claims. A few points were addressed indirectly, likely reflecting antibody limitations and experimental complexity. In particular, endogenous protein localization and direct protein–protein interactions were not sufficiently demonstrated, and metabolic-labeling alternative strategies to prolonged shutdown of transcription by actinomycin D treatment were not performed. However, the newly added functional and quantitative data reduce their need and

support the overall conclusions. Given the breadth of new analyses, the evidence seems to be sufficient for publication. I recommend softening the expressions in text somewhat that implies detailed molecular mechanism or endogenous complex formation. I support publication, provided the remaining comments from other reviewers are resolved.
We thank the reviewer for taking the time to review our manuscript and for the constructive comments. In the revised manuscript we have softened the language describing the proposed model of the FNDC3B/TENT5A/ PTBP1/insulin mRNA complex.

Reviewer #4 (Remarks to the Author):

Comments for Author
Upon editorial request, I have reviewed this manuscript and primarily focused on the authors' reply to original reviewer 3's comments. The authors have substantially revised the manuscript and addressed the majority of the concerns raised in the initial review. The new data and clarifications strengthen both the mechanistic framework and the translational context of the study. Below I provide detailed comments on each point and a few remaining suggestions that could further improve clarity and impact.

Major points.
1. The new data (page 14, Fig. S6j) showing that PTBP1 knockdown does not affect Tent5a-dependent insulin mRNA poly(A) tail length, whereas FNDC3B remains required, is helpful. It supports the conclusion that PTBP1's stabilizing role is largely independent of poly(A) tail elongation. 1) I would recommend that the authors explicitly state in the Results/Discussion that:
PTBP1 is not required for Tent5a-mediated polyadenylation, and that its stabilizing effect is likely mediated via an alternative mechanism (e.g. 3′UTR binding and protection from decay), which remains to be fully elucidated.
We now explicitly state in the revised manuscript that PTBP1 is not required for Tent5a-mediated polyadenylation and that its stabilizing effect is likely mediated via an alternative mechanism (see page 14/15, lines 431-434).

2. The authors examined Tent5a and Tent5c poly(A) tail lengths in the FLAM-seq data and did not observe significant changes upon Tent5a overexpression. This is a reasonable approach to test for a simple auto-regulatory loop via tail extension. I appreciate the link back to the SignalP-TM enrichment analysis, which is consistent with Tent5a/C preferentially targeting transcripts encoding secreted/ER-targeted proteins. I would only suggest that the authors ensure that this negative result is briefly and clearly described in the main text (not exclusively in the Supplement), as this directly answers an explicit mechanistic question.
The data showing that Tent5a and Tent5c poly(A) tail lengths do not change upon Tent5a overexpression was shown in Figures 6,7 for 'Reviewers only'. In response to the suggestion of Reviewer 4, these data are now shown as new Figures 3i and 3j, and the results are described in the main text on page 9, lines 266–270.

3. The new experiments in "healthy" human islet microtissues (hIsMTs) cultured under glucotoxic conditions and infected with AAV–TENT5A are a strong addition.

The observation that a ~2-fold increase in TENT5A expression enhances insulin content under stress, but not in standard culture conditions, is particularly compelling and directly addresses the translational concern. It would be helpful to clearly state (e.g. in the Results) whether the increase in insulin content is accompanied by changes in GSIS and how these changes compare between basal and stimulated states.
We are now clearly stating in the Result section that the increase in insulin content is accompanied by a slight increase in GSIS (not reaching significance) under high glucose stimulation and that this is only seen in metabolically stressed islet microtissues (see pages 10/11, lines 309 – 316).

4. The entire mechanistic framework presented in the manuscript relies on INS-1E cell-based perturbations and ex vivo islet assays. While these experiments are valuable, they remain insufficient to establish the physiological relevance of Tent5a in β-cell function and compensation. Given that the authors propose Tent5a as a key regulator of insulin mRNA stability and β-cell adaptive capacity, in vivo loss-of-function evidence is essential. I would expect genetic validation, ideally through a β-cell–specific Tent5a knockout model (βTent5aKO), to substantiate the central claims of the study.
The referee might have missed that our manuscript includes a Tent5a knockout model in form of CRISPR-generated Tent5aKO β-cells. The data shown in Figures 4h, I, j, and n show the opposite phenotype of Tent5a overexpression, namely markedly reduced Insulin mRNA levels and insulin content. There are only global Tent5a knockout mice available, which have a complex phenotype including osteogenesis imperfecta, reductions in collagen synthesis and serum albumin levels, skeletal abnormalities, kyphosis, and reduced body weight compared to wildtype littermates. While neither exocrine nor endocrine pancreas was studied in these mice, they would not be a good model for characterizing β-cell-specific defects. However, the generation and characterization of conditional β-cell-specific Tent5a KO mice would take an extra 1-2 years and therefore out of scope for the current study.

Response to REVIEWERS' COMMENTS

Reviewer #1 (Remarks to the Author):

During the second round of revision, Silva et al. have addressed most of my concerns:

Q1. The information regarding TENT5A has now been appropriately included in the section describing the screen (Fig. 1 data).

Q2. The "lorem" text has been removed from Fig. 5j.

Q3. The coIP-WB in Fig. 6h has been improved. However, as a minor editorial comment, the labels remain slightly misaligned and should be corrected for clarity.

Q4. The blot demonstrating the efficiency of siRNAs against FNDC3a/b in Fig. S6g has been improved.

Q5. The AlphaFold 3 model statistics are still not included in the main text or in the relevant figure panel. Additionally, the promised dedicated section in the Methods chapter describing the AlphaFold 3 analysis could not be identified. This information remains necessary for proper evaluation of the structural modeling.
We thank the reviewer for this observation and apologize for our oversight. This methods section has now been included in the manuscript on (Page 36, Lines 1335–1343)

Thus Silva et al. have addressed most of my questions. I recommend acceptance on condition that the Methods sections is revised to include a clear description of AlphaFold 3 modeling.

Reviewer #4 (Remarks to the Author):

The authors have clarified the relationship between PTBP1 and Tent5a-mediated polyadenylation, and the additional experiments using human islet microtissues strengthen the translational relevance of the study. The revised model describing the roles of Tent5a, FNDC3B, and PTBP1 is also helpful for understanding the proposed mechanism.

While genetic in vivo validation (e.g., β-cell–specific Tent5a knockout models) would provide a definitive test of the proposed physiological role of Tent5a in β-cell compensation, the current study already provides substantial mechanistic and functional evidence across multiple experimental systems.

Overall, the revisions satisfactorily address the major concerns raised previous reviewer

3, and the manuscript now provides a clearer mechanistic framework linking cytoplasmic polyadenylation to beta-cell compensation.

We thank the reviewers for reading our manuscript and for their constructive comments.
